# Towards a Computable Data Corpus of Temporal Correlations between Drug Administration and Lab Value Changes

**DOI:** 10.1371/journal.pone.0136131

**Published:** 2015-08-24

**Authors:** Axel Newe, Stefan Wimmer, Antje Neubert, Linda Becker, Hans-Ulrich Prokosch, Matthias W. Beckmann, Rainer Fietkau, Christian Forster, Markus Friedrich Neurath, Georg Schett, Thomas Ganslandt

**Affiliations:** 1 Chair of Medical Informatics, Friedrich-Alexander-University Erlangen-Nuremberg, Erlangen, Germany; 2 Department of Paediatrics and Adolescent Medicine, University Hospital Erlangen, Erlangen, Germany; 3 Institute of Psychology, Friedrich-Alexander-University Erlangen-Nuremberg, Erlangen, Germany; 4 Department of Gynecology and Obstetrics, University Hospital Erlangen, Erlangen, Germany; 5 Department of Radiation Therapy, University Hospital Erlangen, Erlangen, Germany; 6 Department of Internal Medicine 4, University Hospital Erlangen, Erlangen, Germany; 7 Department of Internal Medicine 1, University Hospital Erlangen, Erlangen, Germany; 8 Department of Internal Medicine 3, University Hospital Erlangen, Erlangen, Germany; 9 Medical Centre for Information and Communication Technology, University Hospital Erlangen, Erlangen, Germany; University of Chicago, UNITED STATES

## Abstract

**Background:**

The analysis of electronic health records for an automated detection of adverse drug reactions is an approach to solve the problems that arise from traditional methods like spontaneous reporting or manual chart review. Algorithms addressing this task should be modeled on the criteria for a standardized case causality assessment defined by the World Health Organization. One of these criteria is the temporal relationship between drug intake and the occurrence of a reaction or a laboratory test abnormality. Appropriate data that would allow for developing or validating related algorithms is not publicly available, though.

**Methods:**

In order to provide such data, retrospective routine data of drug administrations and temporally corresponding laboratory observations from a university clinic were extracted, transformed and evaluated by experts in terms of a reasonable time relationship between drug administration and lab value alteration.

**Result:**

The result is a data corpus of 400 episodes of normalized laboratory parameter values in temporal context with drug administrations. Each episode has been manually classified whether it contains data that might indicate a temporal correlation between the drug administration and the change of the lab value course, whether such a change is not observable or whether a decision between those two options is not possible due to the data. In addition, each episode has been assigned a concordance value which indicates how difficult it is to assess. This is the first open data corpus of a computable ground truth of temporal correlations between drug administration and lab value alterations.

**Discussion:**

The main purpose of this data corpus is the provision of data for further research and the provision of a ground truth which allows for comparing the outcome of other assessments of this data with the outcome of assessments made by human experts. It can serve as a contribution towards systematic, computerized ADR detection in retrospective data. With this lab value curve data as a basis, algorithms for detecting temporal relationships can be developed, and with the classification made by human experts, these algorithms can immediately be validated. Due to the normalization of the lab value data, it allows for a generic approach rather than for specific or solitary drug/lab value combinations.

## Introduction

It is a well-known fact that all drugs have both benefits and the potential for harm. Even under circumstances of flawless application (i.e. correct indication, dose, consideration of contraindications etc.), a drug can still have unwanted and possibly harming effects [**1**]. These harming effects are a known common cause of iatrogenic morbidity and are among the common causes of death in many countries [**1,2**].

Pharmacovigilance is the systematic and continual monitoring of drugs with the aim to discover, assess, understand and prevent related adverse effects and to take appropriate measures to minimize related risks [**1**]. The most commonly used method for the detection of adverse drug reactions (ADRs) is mandatory spontaneous reporting, although this practice is limited by several systematic shortcomings (e.g. under-reporting & bias) [**3**]. While an estimated third of hospital admissions is accompanied by ADRs, up to 90 percent of ADRs remain undetected in hospital settings [**4**]. The manual chart review–the gold standard in pharmacoepidemiology–is in contrast rather time consuming and expensive [**3**].

The analysis of electronic health records (EHRs) for detecting ADRs is (in comparison to mandatory spontaneous reporting and manual chart review) a relatively new approach [**5**]. One of the most common features of EHRs adopted by (U.S.) hospitals is an electronic laboratory reporting system [**6**] and laboratory data have been identified as suitable parameters for the detection of ADRs [**3,7,8**]. Reference work that provides information about known influence of drug intake on laboratory test results is amply available [**9,10,11**].

The World Health Organization (WHO) in collaboration with the Uppsala Monitoring Centre (UMC) defined a set of criteria for a standardized case causality assessment of ADRs [**12**]. In order to detect ADRs by means of EHR data analysis, relevant algorithms should be modeled on these criteria as archetypes.

One of these criteria is the temporal relationship between drug intake and the occurrence of a reaction or a laboratory test abnormality. It is obvious that some kind of time relationship between drug intake and an ADR must exist. However, the kind of this relationship can range from an immediately anaphylactic reaction to congenital defects that become evident months after intake (whereof the thalidomide tragedy of the 1960s [**13**] is the probably best known example). These two extremes are not the main focus of EHR analysis: immediate reactions are usually obvious and very long term reactions are most likely very hard to detect by means of EHRs. Short term reactions, however, are well suited to be unveiled by EHR analysis–especially those of a single hospital stay, because it is likely that much of the necessary information and data are available.

An algorithm that is aimed to detect temporal correlations between drug intake and lab test abnormalities within such a single hospital stay needs a ground truth on which it can be validated. This article presents a first version of such a ground truth data corpus that is based on real-world data and that can be used for the development and for the validation of algorithms for the detection of temporal correlations between drug intake and lab value alterations.

## Methods

### Ethics

Both the Ethics Committee of the Friedrich-Alexander-University Erlangen-Nuremberg and the Data Protection Officer of the University Clinic of Erlangen were consulted before the study.

Based on the description of the procedure as detailed below, the Ethics Committee concluded and confirmed in writing that no formal approval was required as regards the participation of human subjects and the usage of the underlying data in general.

The Data Protection Officer approved in writing the usage and publication of the underlying anonymized data.

### Raw Data Collection & Preparation

An anonymous excerpt of the i2b2 research data warehouse [**14**] of the University Clinic of Erlangen, Germany, was generated, comprising the following retrospective data:

- All administrations of Filgrastim (ATC: L03AA02) from six different departments (Oncology, Gynecology, Radiotherapeutics, Gastroenterology, Immunology, Nephrology), reduced to the pure calendar date. Dose information was not exported.
- All lab values of Alkaline Phosphatase, Aspartate Transaminase, Alanine Transaminase, Gamma-Glutamyl Transpeptidase, Uric Acid, Urea, Creatinine, Creatine Kinase, Lactate Dehydrogenase, Potassium, Sodium, Myoglobin, Hemoglobin, Leucocytes Count, Granulocytes Count and Thrombocytes Count for patients with the administrations listed above.

The lab parameters were selected based on their commonness and their likeliness to be influenced by Filgrastim. Two groups of ADR likeliness were included, distinguished by their expected frequency (according to the standard categories of ADR frequency as recommended by the Council for International Organizations of Medical Sciences, CIOMS [**15**], and based on the data of the knowledge base described in [**3**]):

1. lab parameters with an expected ADR frequency of “very common” (i.e. ADR frequency ≥ 1/10) and
2. lab parameters with an expected ADR frequency of “rare” (i.e. ADR frequency ≥ 1/10^4^ and < 1/10^3^).

In addition to that, lab parameters with an *expected* effect (i.e. the drug is intended to have an influence on the parameter) were also included.

The choice of Filgrastim was based on three considerations:

1. the intended effect of Filgrastim is to stimulate the proliferation and differentiation of granulocytes, thus probably having direct influence on two of the selected lab parameters (Leucocytes Count, Granulocytes Count),
2. possible ADRs and the affected lab parameters are known from [**3**] and
3. its administration is well documented.

A total of 4332 cases with Filgrastim administrations were extracted from the i2b2. The first step after data extraction was to find solitary episodes of drug administrations. This was achieved by searching for continuous administrations with administration-free intervals of at least 14 days before the first administration and administration-free intervals of at least 14 days after the last administration, respectively. A maximum of one administration-free day was allowed during an episode. Although a minimum administration length was not defined explicitly, it arose implicitly from the lab value selection criteria (5 days, see below).

In a second step, the temporally corresponding lab values were identified. Only drug administration episodes with at least five lab value observations before, during, and after the actual episode were considered for further processing. The lab value observations before and after the administration episode had to be recorded within seven days. During this process, Alanine Transaminase, Myoglobin and Thrombocytes Count dropped out of the list of lab parameters, because no matching episodes were found.

The lab values were then normalized so that the respective patient specific reference interval fell within the range of [0..1] (i.e., the patient specific lower border value maps to 0 and the patient specific upper border value maps to 1), using the following formula:

$$LV_{n}=\frac{LV_{a}-BV_{l}}{BV_{u}-BV_{l}}$$ (1)

where LV_n_ is the normalized lab value, LV_a_ is the actual (absolute) lab value, BV_l_ is the patient specific lower border value and BV_u_ is the patient specific upper border value.

An example: let the patient specific reference range of Alkaline Phosphatase be 30–120 U/L (BV_l_ = 30 U/L; BV_u_ = 120 U/L). In this case, absolute values map as follows: 15 U/L → -0.16; **30 U/L → 0.0**; 100 U/L → 0.78; **120 U/L → 1.0**; 220 U/L → 2.11. By application of this normalization, only the absolute values were changed while the shape of curves and the relative values were kept (especially in comparison to the reference interval). Since the normal (“reference”) values of laboratory parameters are patient-specific (depending on sex, age, etc.) and also laboratory-specific (depending on the exact method used for determining the value), the precise figures used for conversion are not listed here.

The purpose of this normalization was to make impossible any inference to the underlying lab value while keeping the shape of curves, the relative values and the reference interval information.

**Fig 1:**
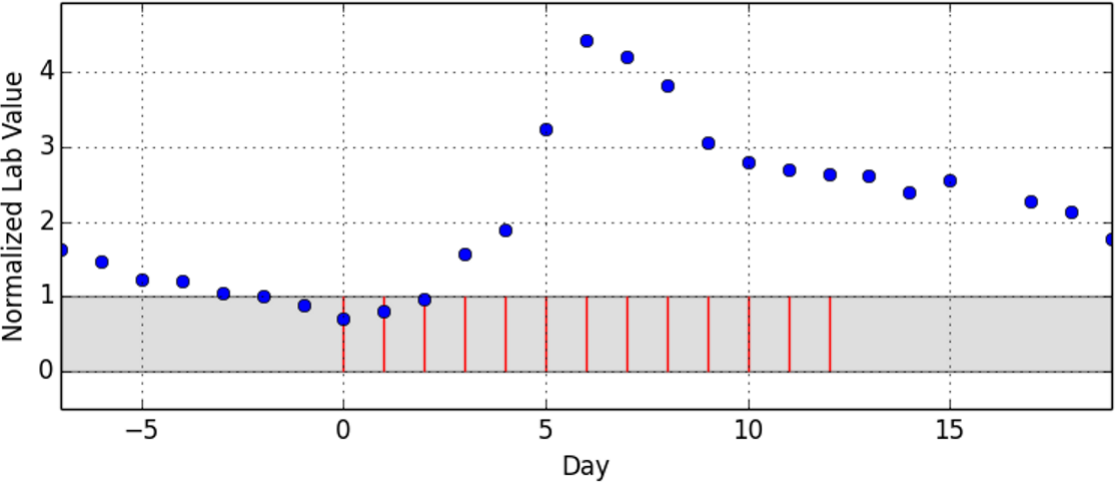
Example of extracted drug administration days (red bars) with corresponding normalized lab values (blue dots). A complete episode consist of a starting administration-free interval of seven days with at least five lab value observations, a period of continuous administration (max. one administration-free day) with at least five lab value observations and finally another administration-free interval of seven days with at least five lab value observations.

As the last step of data preparation, the drug administration dates were reassigned to a scale of relative days with day 0 being the first day of the administration episode. Fig 1 shows an exemplary episode that does not allow any more inference to the underlying drug, the underlying laboratory parameter or any patient-specific information. A total of 902 episodes of drug administrations with temporarily corresponding lab value observations were distilled out of the 4332 initial cases.

Finally, a random selection of 400 out of these 902 episodes was sampled to constitute the basis for the data corpus. A detailed breakdown can be found in Table 1.

**Table 1:** Distribution and characteristics of lab parameters in the raw data and in the data corpus.

Lab parameter	Expected ADR frequency	Quantity^1^		Number of assessments^2^			Intricacy distribution^2^		
		in raw data	in data corpus	temporal correlation	no change	no assessment	low	medium	high
Alanine Transaminase	very common	no episodes^3^	-	-	-	-	-	-	-
Alkaline Phosphatase	very common	58 (6.4%)	27 (6.8%)	17 (63.0%)	9 (33.3%)	1 (3.7%)	13 (48.1%)	7 (25.9%)	7 (25.9%)
Aspartate Transaminase	very common	77 (8.5%)	36 (9.0%)	14 (38.9%)	16 (44.4%)	6 (16.7%)	14 (38.9%)	6 (16.7%)	16 (44.4%)
Creatine Kinase	very common	35 (3.9%)	18 (4.5%)	10 (55.6%)	8 (44.4%)	0 (0.0%)	14 (77.8%)	1 (5.6%)	3 (16.7%)
Creatinine	rare	157 (17.4%)	70 (17.5%)	8 (11.4%)	56 (80.0%)	6 (8.6%)	41 (58.6%)	12 (17.1%)	17 (24.3%)
Gamma-Glutamyl Transpeptidase	very common	77 (8.5%)	35 (8.8%)	19 (54.3%)	7 (20.0%)	9 (25.7%)	8 (22.9%)	3 (8.6%)	24 (68.6%)
Granulocytes Count	expected	3 (0.3%)	1 (0.3%)	0 (0.0%)	0 (0.0%)	1 (100.0%)	0 (0.0%)	1 (100.0%)	0 (0.0%)
Hemoglobin	rare	4 (0.4%)	1 (0.3%)	0 (0.0%)	1 (100.0%)	0 (0.0%)	1 (100.0%)	0 (0.0%)	0 (0.0%)
Lactate Dehydrogenase	very common	75 (8.3%)	43 (10.8%)	26 (60.5%)	12 (27.9%)	5 (11.6%)	16 (37.2%)	10 (23.3%)	17 (39.5%)
Leucocytes Count	expected	78 (8.7%)	31 (7.8%)	16 (51.6%)	14 (45.2%)	1 (3.2%)	16 (51.6%)	9 (29.0%)	6 (19.4%)
Myoglobin	rare	no episodes^3^	-	-	-	-	-	-	-
Potassium	rare	145 (16.1%)	59 (14.8%)	9 (15.3%)	40 (67.8%)	10 (16.9%)	28 (47.5%)	13 (22.0%)	18 (30.5%)
Sodium	rare	139 (15.4%)	53 (13.3%)	3 (5.7%)	49 (92.5%)	1 (1.9%)	36 (67.9%)	7 (13.2%)	10 (18.9%)
Thrombocytes Count	very common	no episodes^3^	-	-	-	-	-	-	-
Urea	very common	24 (2.7%)	11 (2.8%)	5 (45.5%)	3 (27.3%)	3 (27.3%)	3 (27.3%)	2 (18.2%)	6 (54.5%)
Uric Acid	very common	30 (3.3%)	15 (3.8%)	6 (40.0%)	5 (33.3%)	4 (26.7%)	5 (33.3%)	5 (33.3%)	5 (33.3%)
All	very common	376 (41.7%)	185 (46.3%)	97 (52.4%)	60 (32.4%)	28 (15.1%)	73 (39.5%)	34 (18.4%)	78 (42.2%)
All	rare	445 (49.3%)	183 (45.8%)	20 (10.9%)	146 (79.8%)	17 (9.3%)	106 (57.9%)	32 (17.5%)	45 (24.6%)

^1^ Percent values are relative to the total number of datasets in the raw data (902) / data corpus (400).

^2^ Percent values are relative to the frequency in the data corpus.

^3^ No episodes with at least five observations before, during, and after the drug administration were found.

### Data Assessment

**Fig 2:**
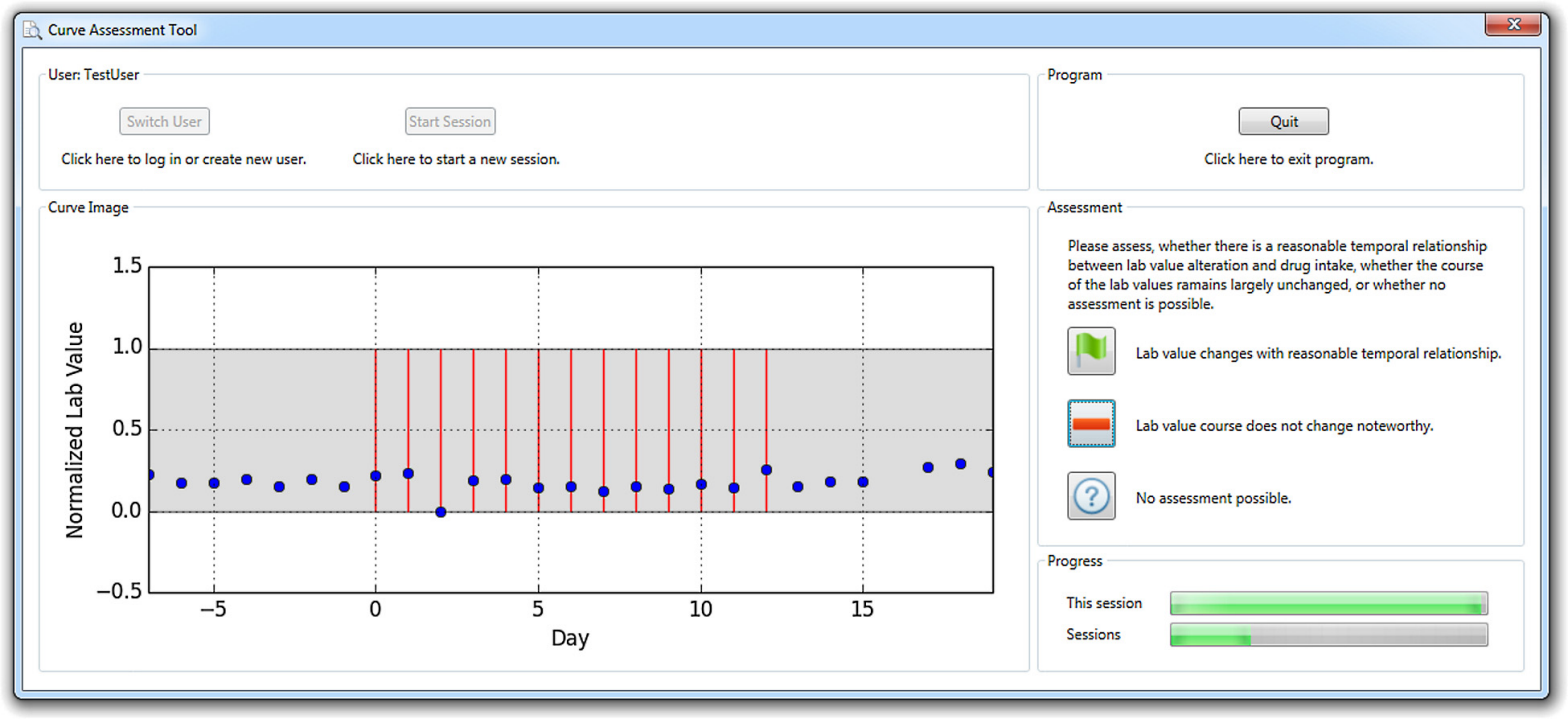
Screenshot of the software tool used for the assessment of the data.

An expert group of three physicians, four pharmacists, three mathematicians and one physicist (seven male & four female, age 35.4y ± 8.2y) assessed the data. The randomly selected 400 data episodes described before were exported to curve plots in lossless Portable Network Graphics (PNG) file format and graphically presented to each of the experts in random order by means of a dedicated software tool (“Curve Assessment Tool”, CAT; Fig 2). For each episode, the study participants were asked to classify the curve graph in terms of a reasonable time relationship between drug administration and lab value alteration into one out of three categories of a nominal scale (the terms in brackets are the abbreviated classifications used for further reference):

- “Lab value changes with reasonable temporal relationship.”(temporal correlation)
- “Lab value course does not change noteworthy.” (no change)
- “No assessment possible.”(no assessment)

The 400 curves were processed twice. Each of the two rounds was divided into four sessions of 100 curves to minimize fatigue effects. The first round was intended to make the assessors familiar with the tool and to give them a feel for the task and the curves. The whole process was then repeated in a second round to actually collect the assessment data (the results of the first familiarization round were disregarded). This second round was performed after a pause of at least three nights after the last session of the first round. By means of this break, reminiscence effects should be minimized while keeping alive the training for the general methodology.

The presentation of the lab value curves (Figs 1 and 2) resembled the Relative Multigraph used by Torsvik et.al. [**16**], but rather than using a partially logarithmic scale for the ordinate axis, a completely linear scale was used. The reference interval was highlighted and the events of drug administrations were presented as vertical bars. Therefore, the graphs contained no information as regards the underlying drug or the observed lab value. This enabled focusing on the pure temporal relationship and should eliminate possible expertise-related bias.

In order to test the feasibility of the procedure, a pre-test was performed before the actual assessment. The pre-test group consisted of two mathematicians and two physicists (one male & one female each, age 29.5y ± 5.2y). Participants of this profession were selected under assumption that they were familiar with the reading and interpretation of data curves.

Before using the software, all participants were informed about the purpose of the study and about the usage of the data. The software did not allow starting the data collection before a participant had explicitly declared consent. No incentives were offered.

### Data Classification

Each curve was finally classified according to the majority of the rater’s votes. Possible standoffs would have been classified as “no assessment”, but this case did not occur. Since one of the main goals of this study was to create a data corpus that reflects real-world data, curves with low inter-rater concordance were not filtered out. Instead, in order to reflect the incertitude, a “concordance value” was calculated by dividing the number of assessors that agreed to the majority call by the total number of assessors (11).

In order to facilitate the following description of the results and the discussion, a rule-based “intricacy” classification will be used, based on the variance of the rater’s votes:

- “low” for curves with nearly total concordance (i.e., 10 or all 11 raters agreed),
- “medium” for curves with high concordance (8 or 9 raters agreed),
- “high” for curves with lower concordance (7 or less raters agreed).

## Results

### Ground Truth Data Corpus

Of the 400 curve plots, 220 (55.0%) were finally classified as “Lab value course does not change noteworthy” (no change), 133 (33.25%) were classified as “Lab value changes with reasonable temporal relationship” (temporal correlation), and 47 (11.75%) were classified as “No assessment possible” (no assessment).

The “low” intricacy classification applies to 195 of the 400 curves (48.75%). Another 76 (19.0%) belong to the “medium” intricacy group and the remaining 129 (32.25%) are “high” intricacy curves.

The inter-rater agreement, determined by calculating Krippendorff’s α [**17**], was α = 0.453 for the whole data corpus. However, this figure is of very limited value (see Dicussion section) and only presented for the sake of completeness.

For a more detailed breakdown see Table 1 and Table 2. The average length of the drug administration episodes is (9.81 ± 3.96) days and the average length of the overall data episodes (i.e. including the administration-free intervals before and after the administration episode) is (23.36 ± 4.14) days.

**Table 2:** Breakdown of classifications for correlation and intricacy for the Ground Truth Data Corpus (absolute numbers, percent values referred to the total number of curves and in brackets percent values referred to the number of curves of the corresponding intricacy).

Intricacy	No Change		Temporal Correlation		No Assessment		All	
n/a	220	55.00%	133	33.25%	47	11.75%	400	100%
low	138	34.50% (70.77%)	57	14.25% (29.23%)	0	0% (0%)	195	48.75%
medium	41	10.25% (53.95%)	29	7.25% (38.16%)	6	1.50% (7.89%)	76	19.0%
high	41	10.25% (31.78%)	47	11.75% (36.43%)	41	10.25% (31.78%)	129	32.25%

### Availability of Data and Software

The Ground Truth Data Corpus is available in Extensible Markup Language (XML) format as S1 File. This data corpus contains the relative days of drug application, the normalized lab value data with the relative days of their observation and the temporal correlation assessment results (including the majority call and its concordance) for each of the 400 episodes.

The CAT software application described above is available as S2 File. The version published with this article allows for creating different user profiles and for executing the complete assessment procedure as executed during the assessment stage, but it does not require to declare consent with the terms of data collection and data usage as in the version originally utilized. The mandatory pause of 3 days between the two rounds has also been removed in this published version. Since the CAT presents predefined curve plots rather than generating them itself, S2 File also contains all these plots of the assessed curves in PNG format.

In S3 File, the original, anonymized results of the assessment for each curve are available together with a software application (see below) which allows for comparing them with the classification results.

### Reutilization Tool

**Fig 3:**
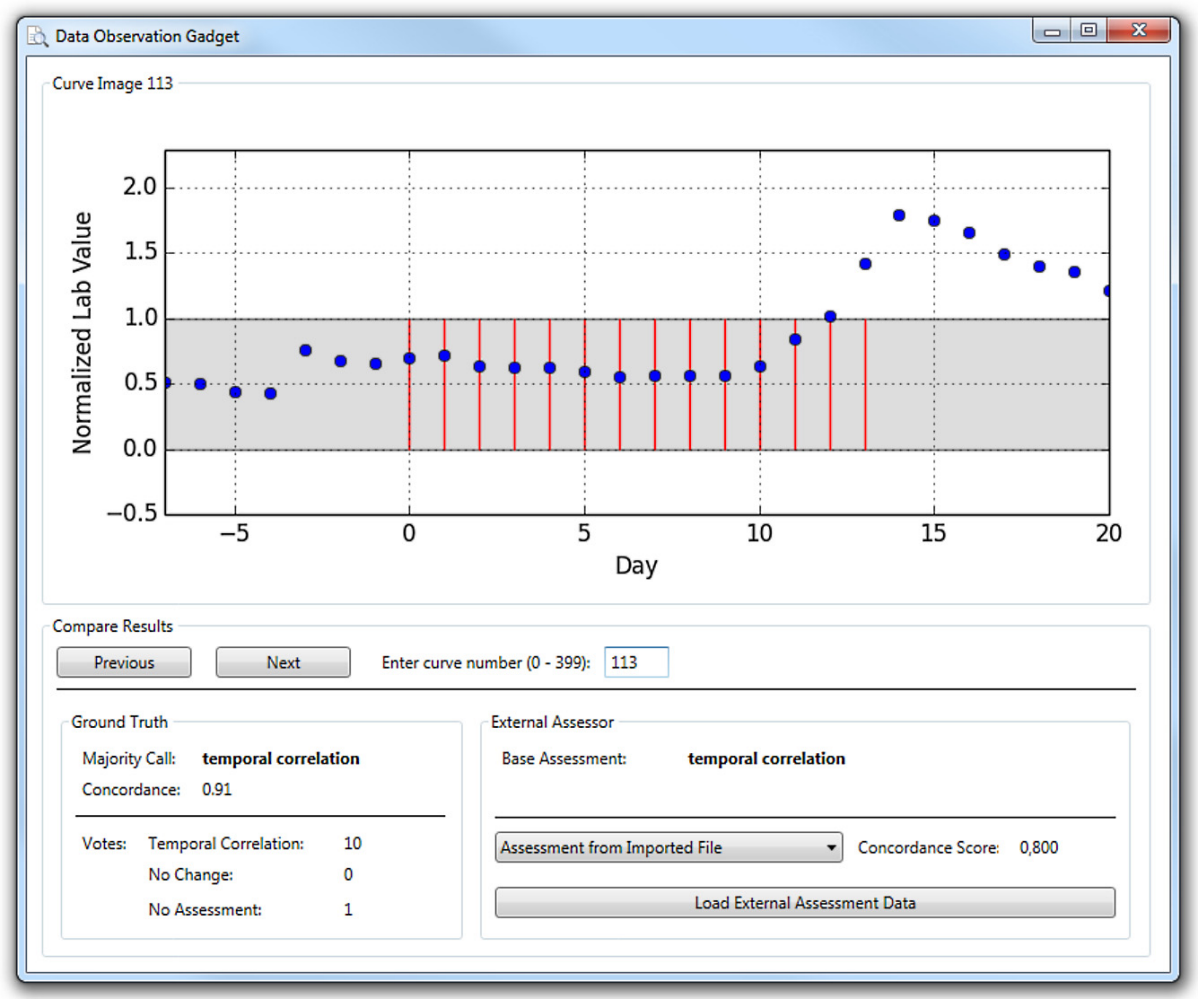
Screenshot of the reutilization tool that allows for comparing other assessment results with the ground truth classification.

Besides the pure Ground Truth Data Corpus, an additional software application (Data Observation Gadget, DOG; Fig 3) for a facile reutilization of this data is available for the Windows/.NET operating environment (S3 File). This tool can be used for two purposes.

First, it allows for a simple review of the Ground Truth Data Corpus. All 400 curves can be browsed and displayed along with the classification and the detailed assessment results.

Second, it allows for importing external assessment data and for comparing this imported data with the Ground Truth classification, including a basic statistical evaluation by means of the Concordance Score (S_C_) described in the Discussion section.

Two different file formats can be imported into the DOG using the “Load External Assessment Data” button. Any complete result data file produced by the CAT is suitable for import. The original results of the pre-test and the actual assessment are included as an example (file “data_external_original_assessment.xml” within S3 File). Using the CAT and the DOG, any interested user can perform her or his own assessment session and compare the personal results to the Ground Truth classification.

**Fig 4:**
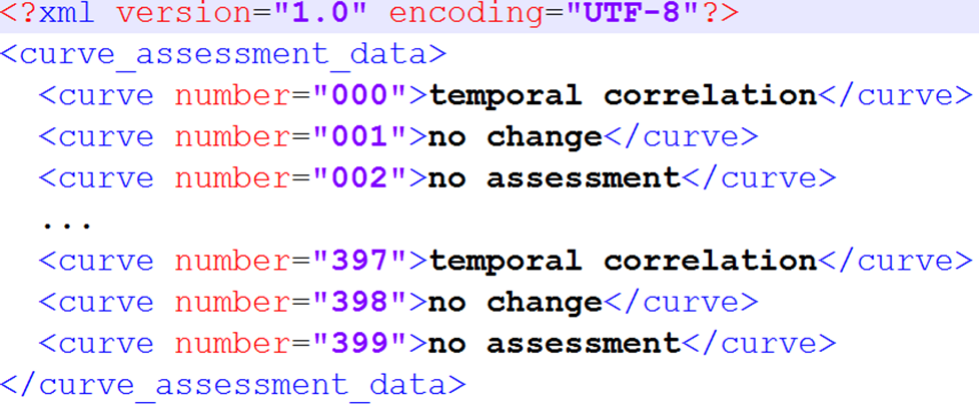
XML structure needed for loading external data into the DOG reutilization tool.

Instead of results from the CAT, any other XML file following the structure described in Fig 4 can be imported into the DOG as well. This allows for comparing the results of any external software with the Ground Truth classification. S3 File contains an example in this format (file “data_external_simple.xml”).

The source code of the DOG is available as open source (S4 File) under CC-BY-SA 4.0 license (https://creativecommons.org/licenses/by-sa/4.0/). Therefore it can be used as a basis for an extension to additional functionality, e.g. a more comprehensive statistical evaluation.

## Discussion

### Results

The presented Ground Truth Data Corpus contains a collection of 400 episodes of normalized laboratory parameter values in temporal context with drug administrations. Each episode has been classified by means of a nominal scale whether it contains data that might indicate a temporal correlation between the drug administration and the change of the lab value course, whether such a change is not observable or whether human experts cannot definitely decide between those two options due to the data. In addition, each episode has been assigned a concordance value which indicates how difficult it is to assess. This reflects the fact that even human experts are subject to ambiguity when faced with a challenging lab value curve. In order to achieve a high reliability of the classifications, the lab value curves were assessed by a total of 11 experts.

One-third of the episodes (33.25%) was classified into the “temporal correlation” category, i.e., the human experts assessed that there was a temporal correlation between the drug administration and the change of a lab value course. This might seem to be an exaggerated quota, but it must be kept in mind that the underlying lab parameters were not only selected by their likeliness to be influenced by *adverse* drug effects, but also by their likeliness to be affected by *intended* drug effects. On the other hand, this figure is interestingly close to the finding by Classen et. al. [**4**], who figured out that one-third of hospital admissions is accompanied by ADRs.

For those lab values with an expected ADR frequency of “very common”, the share of “temporal correlation” is even higher (52.4%), but this is in line with the expectation (i.e. >10%). On the other hand, those lab values with an ADR frequency expected to be “rare” show more temporal correlations than expected (10.9% vs. <0.1%). Considering only results with low and medium intricacy, the figures are similar: 58.9% of the “very common” lab values and 8.0% of the “rare” lab values were classified into the “temporal correlation” category. A chi-squared test shows that the number of temporal correlations in these intricacy groups is significantly higher for the “very common” lab values (63 out of 107) than for the “rare” lab values (11 out of 138): χ^2^(df = 1, N = 245) = 74.09, p < .001.

Even if the ~10% temporal correlations that were found for the curves with expected “rare” ADR rate were assumed to be purely coincidental and if these 10% were therefore simply subtracted from the findings, it is still interesting, that a share of ~40% “temporal correlation” classifications remains for curves with an expected “very common” ADR rate.

However, it must be kept in mind that a temporal correlation does not equal a causal correlation and that it can never be the sole source of evidence for an ADR. Therefore, many of the temporal correlations might be caused by other confounders (especially when derived from data that has been collected from treatment in a university hospital).

The “Leucocytes Count” lab parameter was initially selected due to its expected response to Filgrastim. Although more than a half of the leucocytes curves were assessed as “temporal correlation” (51.6%), this share is far from 100%. Besides the fact that each patient’s response is different, the most probable explanation for this observation is, that the drug was administered in doses that were intended to adjust the leucocytes count to the regular values or in small doses that did not affect the curves in a way that a doubtless temporal correlation could be identified.

**Fig 5:**
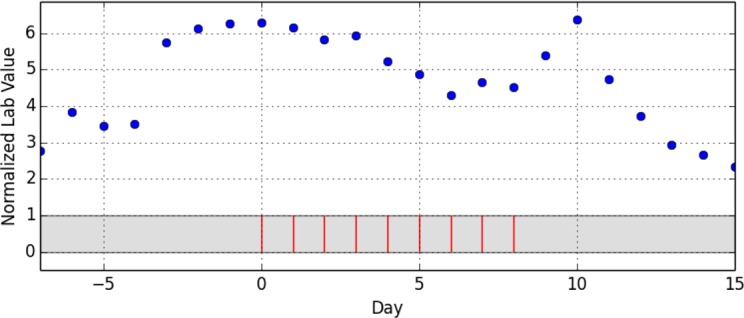
Example for a curve with “no assessment” and high intricacy (curve #129).

It is noticeable, although not amazing, that the number curves that were finally classified as “no assessment possible”rises together with the level of intricacy: while not a single curve was classified into this category concordantly by all experts, 1.5% of the curves with medium intricacy fall into this category as well as 10.25% of the curves with high intricacy. However, it is also noticeable, that only 31.78% (41/129) of the curves with high intricacy fall into the “no assessment” category (see Fig 5 for an example). Therefore, a high level of intricacy does not mean that a curve cannot be assessed at all. Another interesting observation is that the group of curves with high intricacy is the only group where the number of curves that were assessed to express a possible temporal correlation almost equals the number of curves that were assessed to show no sign of temporal correlation (45 vs. 43 curves).

### Purpose & Usage

The main purpose of this data corpus is the provision of data for further research and the provision of a ground truth which allows for comparing the outcome of other assessments of the data with the outcome of assessments made by human experts.

Besides the pure open access availability of such a data collection, which–to our best knowledge–has never been published before, it can serve as a contribution towards systematic, computerized ADR detection in retrospective data. Therefore, it contributes to similar efforts like the “SIDER2” database of phenotypic effects of drugs [**18**], the “OFFSIDES” database of off-label side effects [**19**] and the “TWOSIDES” database of drug-drug interactions [**19**].

With the lab value curve data as a basis, algorithms can be developed, and with the corresponding classification made by human experts, these algorithms can immediately be validated. Due to the normalization of the lab value data, it allows for a generic approach rather than for specific or solitary drug/lab value combinations.

However, about one-third (33.25%) of the assessments was assigned to the high intricacy group (as defined above). In this group, 5–7 out of 11 raters agreed to the majority call. Having three result classes, a high rate of chance agreement is likely for this group, thus limiting the quality of the call and making the classification somewhat arbitrary. Therefore, it should be considered carefully, whether this share of the data corpus is suitable for a desired usage.

### Applicability of Statistical Standard Measures

A quality measure of data that is based on human assessment is the agreement among independent observers. A number of statistical measures are established (Scott’s π [**20**], Cohen’s κ [**21**], Fleiss’ κ [**22**], Krippendorff’s α [**23,24**]) and Hayes et. al. [**25**] discussed the most popular ones, finally recommending Krippendorff’s α as the best suited for nominally scaled data. Therefore, α was calculated for the data assessment, yielding a rather low value of α = 0.453 for the overall data corpus. However, all these measures (including Krippendorff’s α) have corrections for chance agreement and this is a major pitfall, since these corrections presume that all items that were assessed are subject to the same level of difficulty as regards assessing them. The presented data corpus, on the contrary, is derived from real-world data and not constructed or filtered to contain only ideal data. Therefore, it contains elements with different levels of difficulty which impedes the application of standard statistical measures. Nearly half of the data (48.75%) consists of curves with low intricacy and another fifth (19%) was classified as medium intricacy, which means that at least 8 of the 11 raters were concordant for these curves (Table 2).

### Statistical Evaluation Means

In order to provide a useful means for comparing any other (external, e.g. algorithmic) assessment of the Ground Truth data corpus with the assessment made by human experts as proposed above, a simple numerical value is desirable. As discussed above, standard statistical measures for comparing assessments of nominally scaled data are not suitable in this case, since they do not consider item difficulty.

Therefore, we recommend using a defined Concordance Score (S_C_) which is a weighted percent measure [**25**] and considers the difficulty by weighting the false assessments:

$$S_{C}=\frac{\sum_{i=1}^{400}m_{i}*c_{i}}{\sum_{i=1}^{400}c_{i}}$$ (2)

where m_i_ is 1 for a matched assessment, m_i_ is 0 for a mismatched assessment, c_i_ is the concordance value of the curve’s majority call and S_C_ is the Concordance Score. Using this score instead of a statistical standard measure reflects the difficulty of the curves and takes into account that even human experts can be at odds with certain curve progressions. Therefore, it is well suited to compare the outcome of a single external assessment (e.g. by an algorithm) with the classification as determined by human experts, thus validating the results of the external assessment.

### Limitations

The first limitation to be mentioned is the selection of only one single drug (Filgrastim) for generating the data. This can possibly cast doubt on the transferability as regards other drugs, but since the curves generated by the normalization process have very different shapes and characteristics, a monoculture is not recognizable and therefore this limitation can be regarded as not too severe. However, further research should be pursued.

The overall number of 400 curves might be considered rather low, especially since not a single curve with low intricacy was categorized as “no assessment”. However, this magnitude was a reasonable tradeoff between an acceptable number of curves and the effort for the study participants. Future research to add new data is desirable here, too.

The timeframe of the episodes in this data corpus only considers one week before and after the period of drug administration. Therefore, any changes occurring later than one week after the end of the drug administration are not modeled by this data.

Considering only drug administration episodes with at least five lab value observations before, during, and after the actual drug administration might also limit the reuse of this data corpus. However, the minimum of five observations in each phase was selected as a tradeoff and with the computability of the data corpus in mind. Real world episodes might often consist of fever data points, but these types of episodes are also likely to be out of scope of computational processing and algorithms. By reducing the lab value observation time points to a daily scale, any change within a day could not be observed. This was out of scope of this study, though, and in addition, a more granular observation might raise data protection issues.

Furthermore, the presented data corpus in its current version disregards drug dose information and any other possible confounders, like (for example) applied therapies, additional drugs, findings and diagnoses, which are necessary to assess the causality between a drug administration and an ADR. However, it does not raise a claim to be comprehensive. It focuses purely on the temporal correlation which is only one component of causality assessment.

Although the lab values were normalized in order to make them unrecognizable, it cannot be guaranteed that the medical professionals who were in charge to assess them had absolutely no guess which lab value could be underlying. This is due to the fact that not every lab parameter can drop below the lower border and not every lab parameter can e.g. rise tenfold.

The curves that were included into this data corpus were selected randomly out of 902 solitary episodes of drug administrations with corresponding lab value observations. A less strict filtering of the initial 4332 cases of drug administrations might have led to a broader basis. However–in order to focus purely on the temporal relationship, it was necessary to find isolated episodes. Administration-free intervals of one week before and one week after the drug administration were considered to be the minimum to guarantee this isolation. In order to be able to assess the possible temporal correlation between the drug administration and the lab value course, a dense sampling of lab value observations was inevitable. This policy led to a drop-out of many cases with unsteady or infrequent lab value recordings.

At this point it should finally be mentioned that the WHO criteria for a standardized case causality assessment of ADRs refers to the drug *intake* which–strictly speaking–differs from drug *administration*. However, this difference has no practical impact on this work, since it does not really matter if an administrated drug was really ingested. If there was no drug intake, no lab value abnormality would be observable, which could also happen if a drug intake simply did not cause an abnormality.

### Outlook

Although the data corpus presented in this article constitutes a result of its own, it is also rather a milestone than the final outcome of a research effort, since it enables two main branches of future research: extension and usage.

As regards possible extensions, it can be strived for overcoming the limitations mentioned above. By taking into account e.g. drug dose and possible confounders of ADRs, this data corpus of temporal correlations could possibly be extended to a corpus that includes additional correlations and finally maybe a corpus of real causality relations.

Second, the data collection could be extended generally, by e.g. adding data generated from other drugs and laboratory parameters. Since the CAT software provided as S2 File presents curve plots taken from PNG format files, it can directly be re-used by other researchers. The only effort needed is to replace the curve plot image files. However, in order to produce comparable results, the protocol for data generation and data assessment should be kept as closely as possible to the procedure described above.

As regards usage, the curve data can be utilized to develop algorithms for the detection of temporal correlations and the expert assessments of the curves can be utilized to validate these algorithms.

## Conclusion

In this article, we present the first open data corpus of a computable ground truth of temporal correlations between drug administration and lab value alterations. The data consists of normalized laboratory parameter values in temporal context with drug administrations and corresponding expert assessments of possible temporal correlations.

This data corpus can and should serve as a basis for future research. While it covers only a small aspect of the complex relationships of data that must be taken into account in ADR research, it is at least a first step towards common availability of such data.

## Supporting Information

S1 File “temporal_correlation_groundtruth.zip”. The Ground Truth Dataset in zipped XML format.
(ZIP)

S2 File “CAT.zip”. The “Curve Assessment Tool” (CAT) software application. This archive also contains the plots of all curves in Portable Network Graphics (PNG) format.
(ZIP)

S3 File “DOG.zip”. The “Data Observation Gadget” (DOG) software application. This archive also contains the Ground Truth Dataset and the original, anonymized results of the pre-test and the assessment.
(ZIP)

S4 File “DOG-Sources.zip”. The C# source code of the “Data Observation Gadget” (DOG) software application. This source code is licensed under CC-BY-SA 4.0 (https://creativecommons.org/licenses/by-sa/4.0/).
(ZIP)
