# Supplementary figures and images for: Towards a Computable Data Corpus of Temporal Correlations between Drug Administration and Lab Value Changes (part 2 of 2)
Source: PLoS One. 2015 Aug 24;10(8):e0136131. doi: 10.1371/journal.pone.0136131 (PMC4547740; doi:10.1371/journal.pone.0136131)

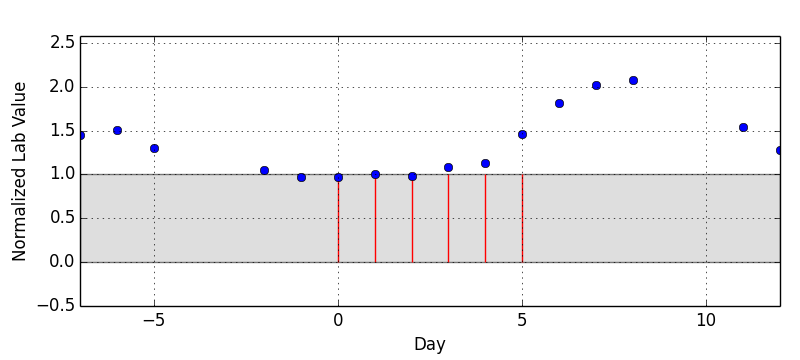

Supplement: S2 File — The “Curve Assessment Tool” (CAT) software application. This archive also contains the plots of all curves in Portable Network Graphics (PNG) format. (ZIP) [file pone.0136131.s002.zip › data/100.png]

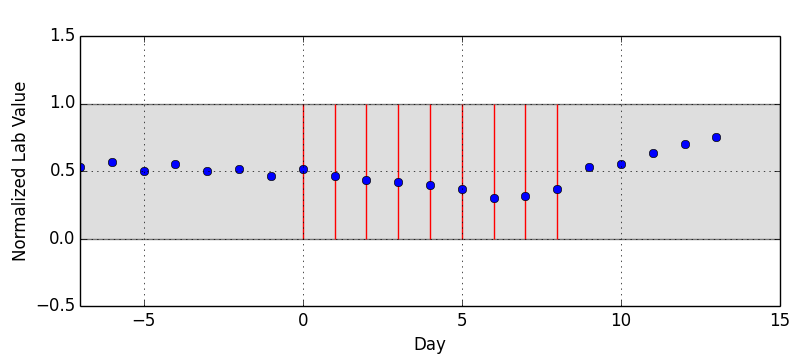

Supplement: S2 File — The “Curve Assessment Tool” (CAT) software application. This archive also contains the plots of all curves in Portable Network Graphics (PNG) format. (ZIP) [file pone.0136131.s002.zip › data/101.png]

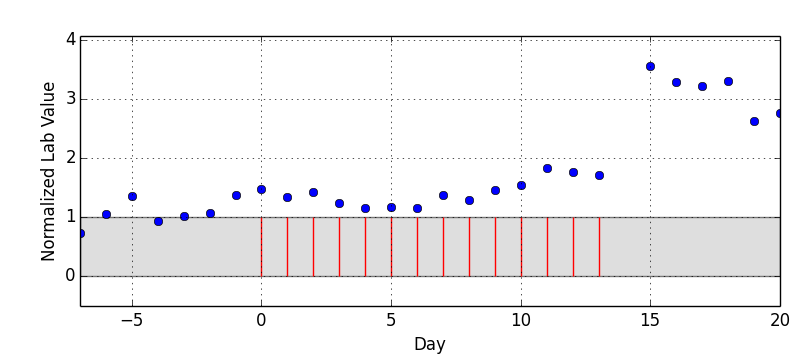

Supplement: S2 File — The “Curve Assessment Tool” (CAT) software application. This archive also contains the plots of all curves in Portable Network Graphics (PNG) format. (ZIP) [file pone.0136131.s002.zip › data/102.png]

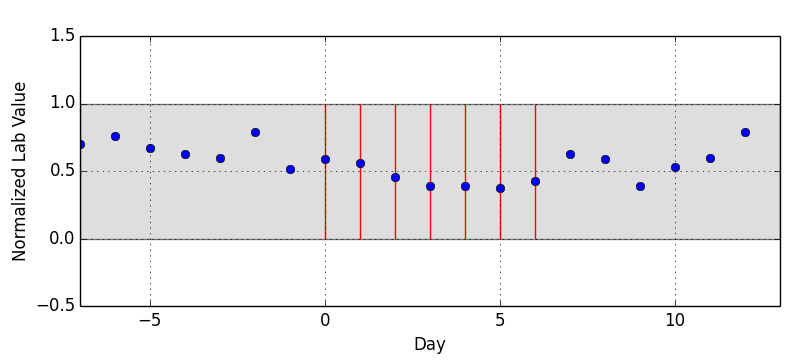

Supplement: S2 File — The “Curve Assessment Tool” (CAT) software application. This archive also contains the plots of all curves in Portable Network Graphics (PNG) format. (ZIP) [file pone.0136131.s002.zip › data/103.png]

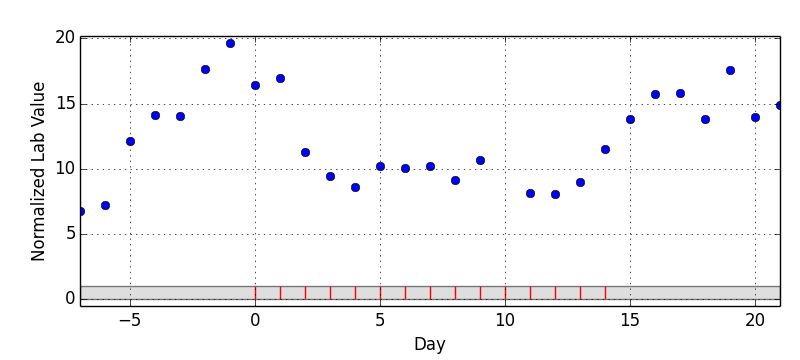

Supplement: S2 File — The “Curve Assessment Tool” (CAT) software application. This archive also contains the plots of all curves in Portable Network Graphics (PNG) format. (ZIP) [file pone.0136131.s002.zip › data/104.png]

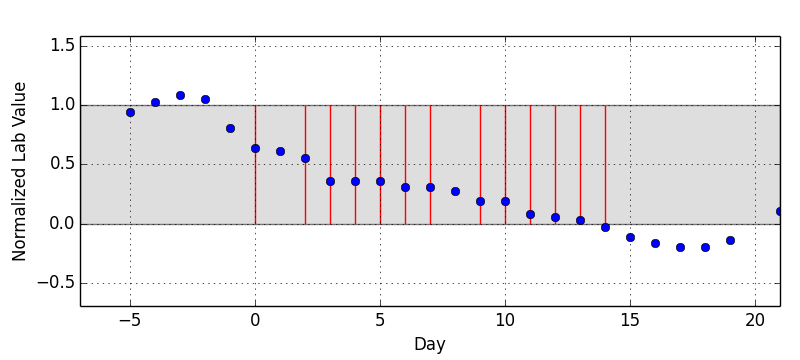

Supplement: S2 File — The “Curve Assessment Tool” (CAT) software application. This archive also contains the plots of all curves in Portable Network Graphics (PNG) format. (ZIP) [file pone.0136131.s002.zip › data/105.png]

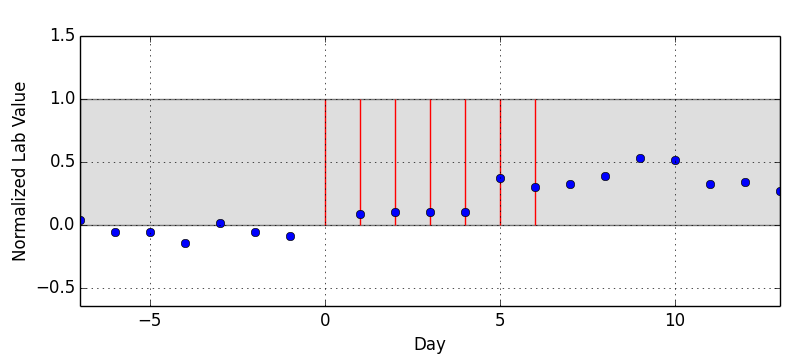

Supplement: S2 File — The “Curve Assessment Tool” (CAT) software application. This archive also contains the plots of all curves in Portable Network Graphics (PNG) format. (ZIP) [file pone.0136131.s002.zip › data/106.png]

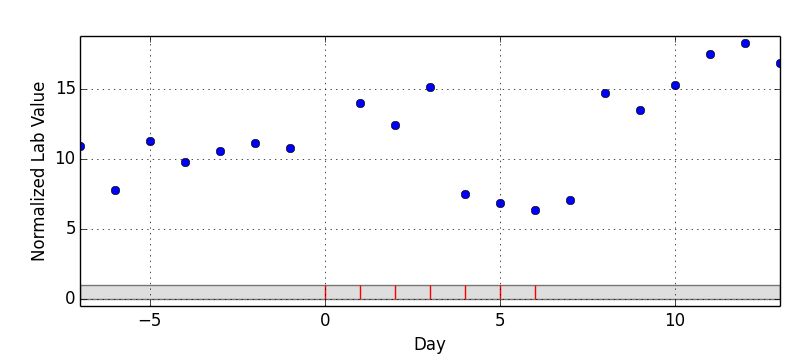

Supplement: S2 File — The “Curve Assessment Tool” (CAT) software application. This archive also contains the plots of all curves in Portable Network Graphics (PNG) format. (ZIP) [file pone.0136131.s002.zip › data/107.png]

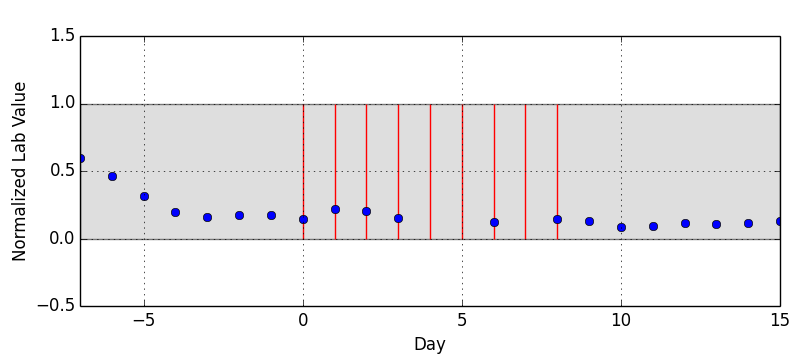

Supplement: S2 File — The “Curve Assessment Tool” (CAT) software application. This archive also contains the plots of all curves in Portable Network Graphics (PNG) format. (ZIP) [file pone.0136131.s002.zip › data/108.png]

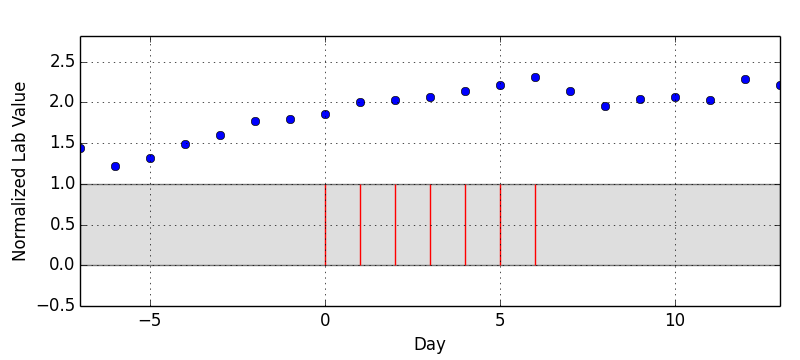

Supplement: S2 File — The “Curve Assessment Tool” (CAT) software application. This archive also contains the plots of all curves in Portable Network Graphics (PNG) format. (ZIP) [file pone.0136131.s002.zip › data/109.png]

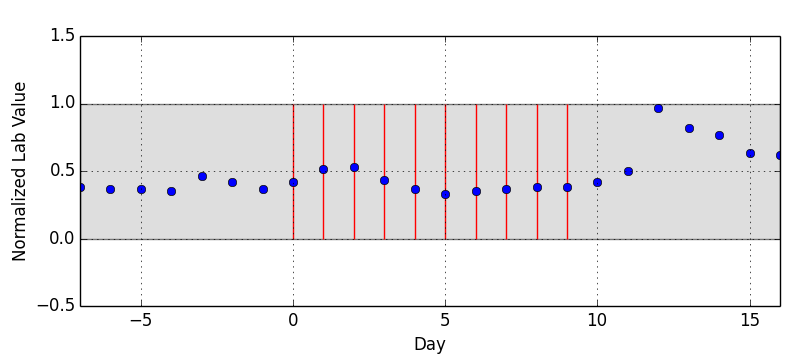

Supplement: S2 File — The “Curve Assessment Tool” (CAT) software application. This archive also contains the plots of all curves in Portable Network Graphics (PNG) format. (ZIP) [file pone.0136131.s002.zip › data/110.png]

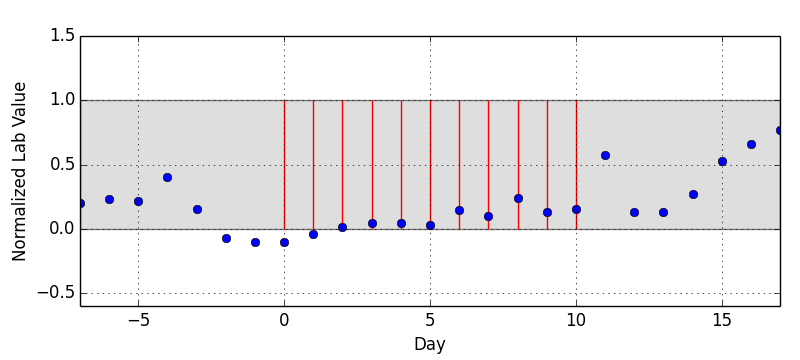

Supplement: S2 File — The “Curve Assessment Tool” (CAT) software application. This archive also contains the plots of all curves in Portable Network Graphics (PNG) format. (ZIP) [file pone.0136131.s002.zip › data/111.png]

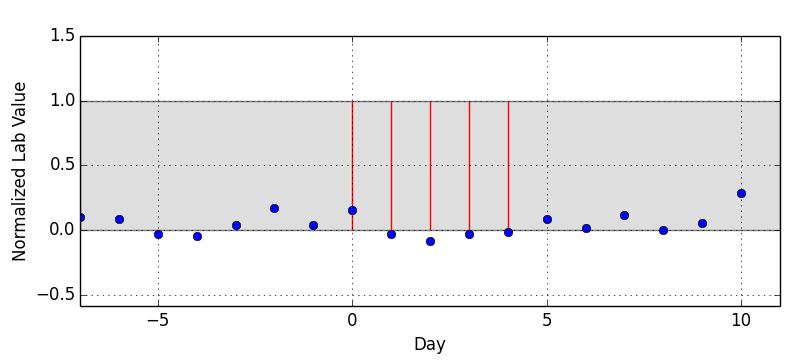

Supplement: S2 File — The “Curve Assessment Tool” (CAT) software application. This archive also contains the plots of all curves in Portable Network Graphics (PNG) format. (ZIP) [file pone.0136131.s002.zip › data/112.png]

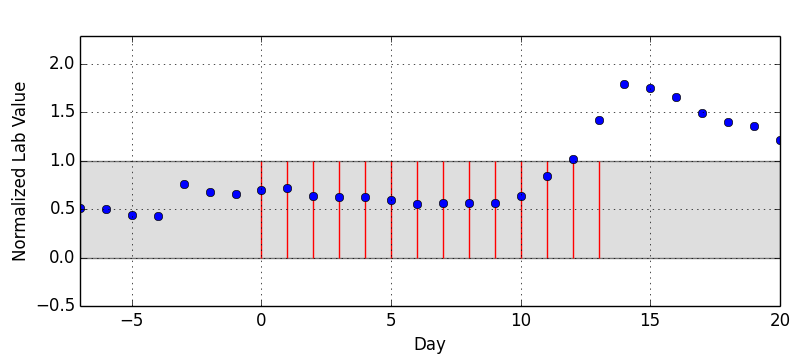

Supplement: S2 File — The “Curve Assessment Tool” (CAT) software application. This archive also contains the plots of all curves in Portable Network Graphics (PNG) format. (ZIP) [file pone.0136131.s002.zip › data/113.png]

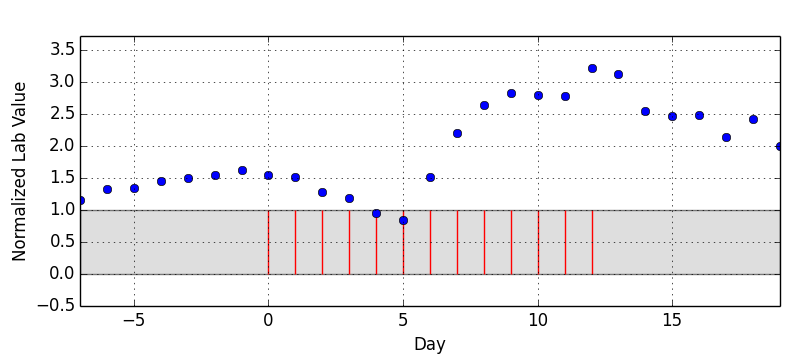

Supplement: S2 File — The “Curve Assessment Tool” (CAT) software application. This archive also contains the plots of all curves in Portable Network Graphics (PNG) format. (ZIP) [file pone.0136131.s002.zip › data/114.png]

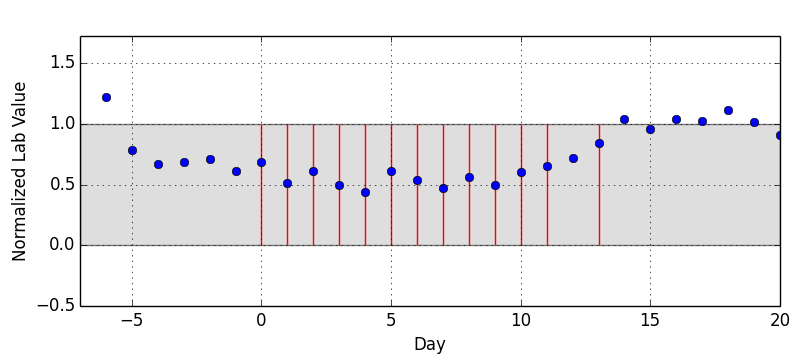

Supplement: S2 File — The “Curve Assessment Tool” (CAT) software application. This archive also contains the plots of all curves in Portable Network Graphics (PNG) format. (ZIP) [file pone.0136131.s002.zip › data/115.png]

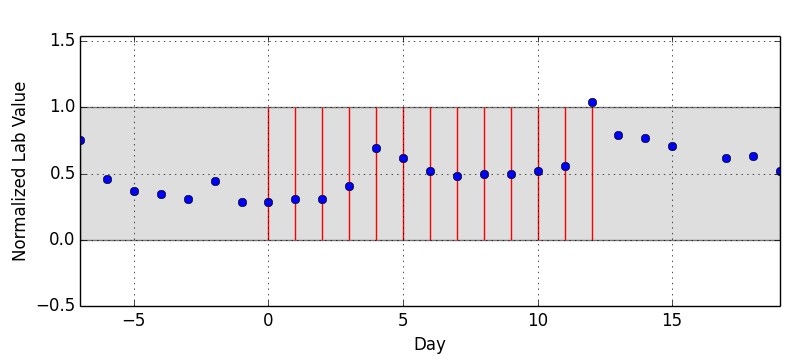

Supplement: S2 File — The “Curve Assessment Tool” (CAT) software application. This archive also contains the plots of all curves in Portable Network Graphics (PNG) format. (ZIP) [file pone.0136131.s002.zip › data/116.png]

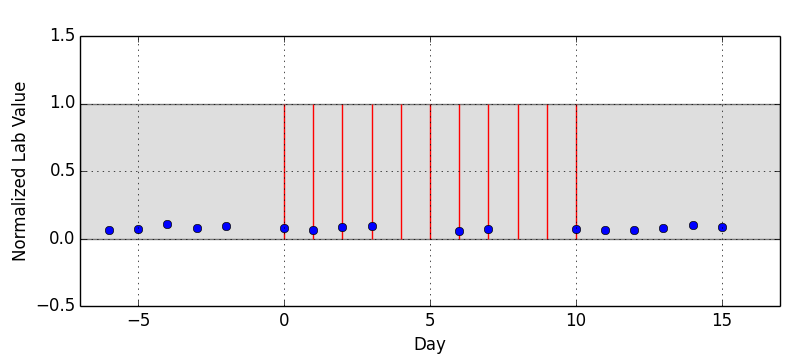

Supplement: S2 File — The “Curve Assessment Tool” (CAT) software application. This archive also contains the plots of all curves in Portable Network Graphics (PNG) format. (ZIP) [file pone.0136131.s002.zip › data/117.png]

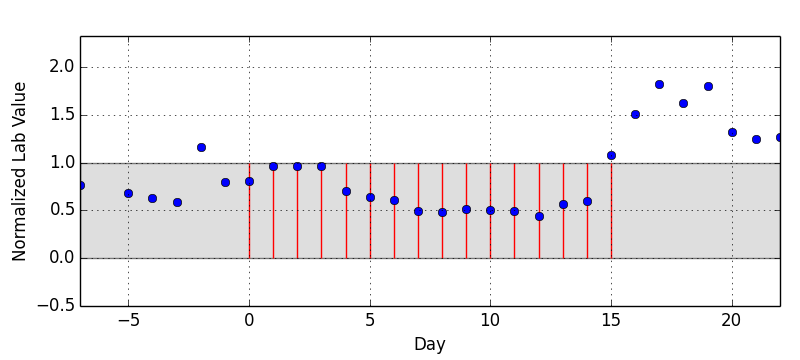

Supplement: S2 File — The “Curve Assessment Tool” (CAT) software application. This archive also contains the plots of all curves in Portable Network Graphics (PNG) format. (ZIP) [file pone.0136131.s002.zip › data/118.png]

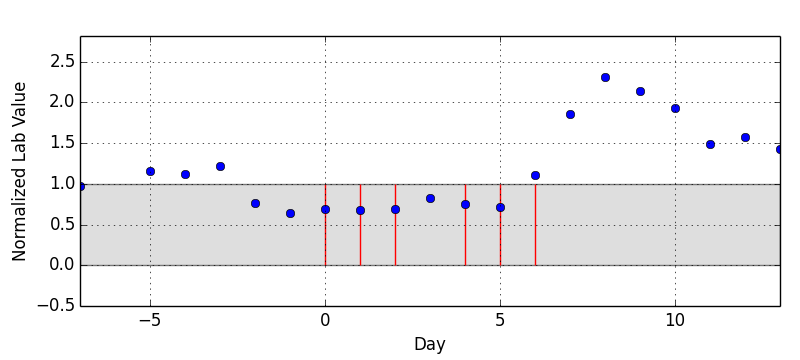

Supplement: S2 File — The “Curve Assessment Tool” (CAT) software application. This archive also contains the plots of all curves in Portable Network Graphics (PNG) format. (ZIP) [file pone.0136131.s002.zip › data/119.png]

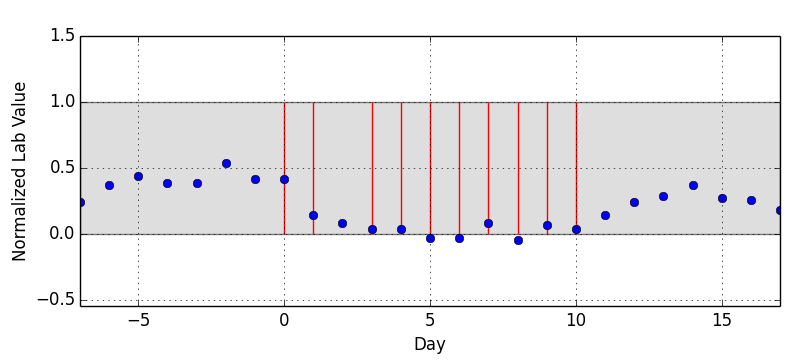

Supplement: S2 File — The “Curve Assessment Tool” (CAT) software application. This archive also contains the plots of all curves in Portable Network Graphics (PNG) format. (ZIP) [file pone.0136131.s002.zip › data/120.png]

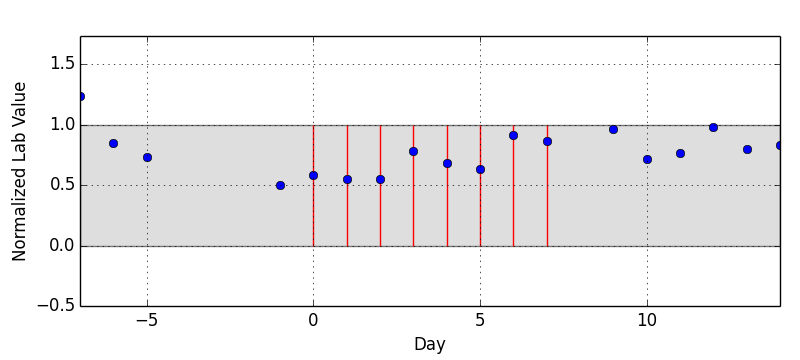

Supplement: S2 File — The “Curve Assessment Tool” (CAT) software application. This archive also contains the plots of all curves in Portable Network Graphics (PNG) format. (ZIP) [file pone.0136131.s002.zip › data/121.png]

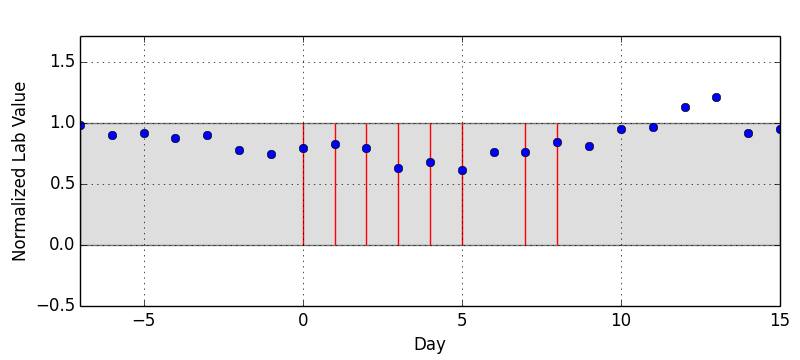

Supplement: S2 File — The “Curve Assessment Tool” (CAT) software application. This archive also contains the plots of all curves in Portable Network Graphics (PNG) format. (ZIP) [file pone.0136131.s002.zip › data/122.png]

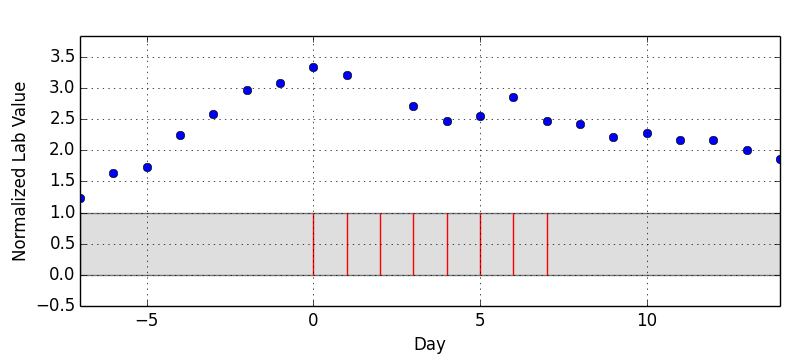

Supplement: S2 File — The “Curve Assessment Tool” (CAT) software application. This archive also contains the plots of all curves in Portable Network Graphics (PNG) format. (ZIP) [file pone.0136131.s002.zip › data/123.png]

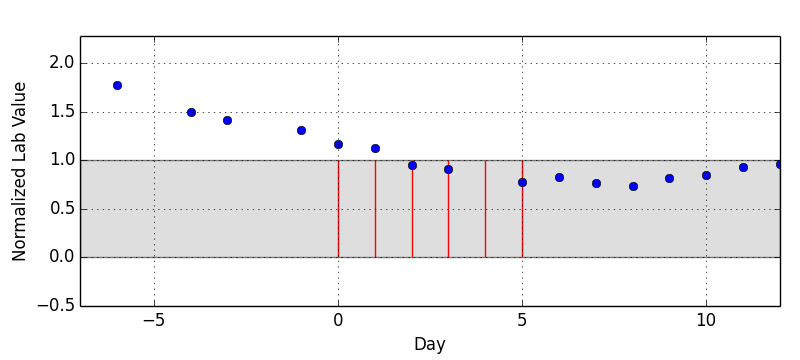

Supplement: S2 File — The “Curve Assessment Tool” (CAT) software application. This archive also contains the plots of all curves in Portable Network Graphics (PNG) format. (ZIP) [file pone.0136131.s002.zip › data/124.png]

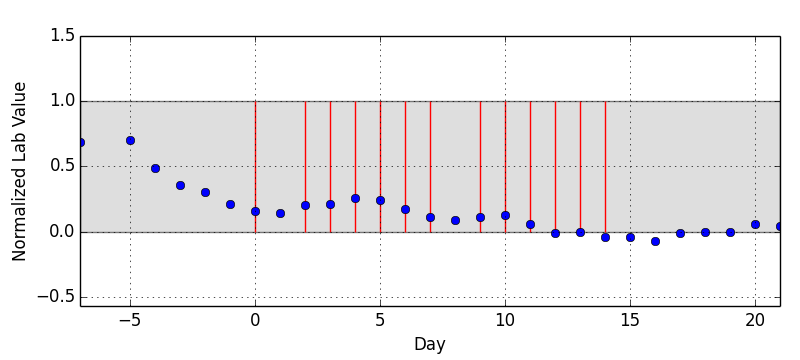

Supplement: S2 File — The “Curve Assessment Tool” (CAT) software application. This archive also contains the plots of all curves in Portable Network Graphics (PNG) format. (ZIP) [file pone.0136131.s002.zip › data/125.png]

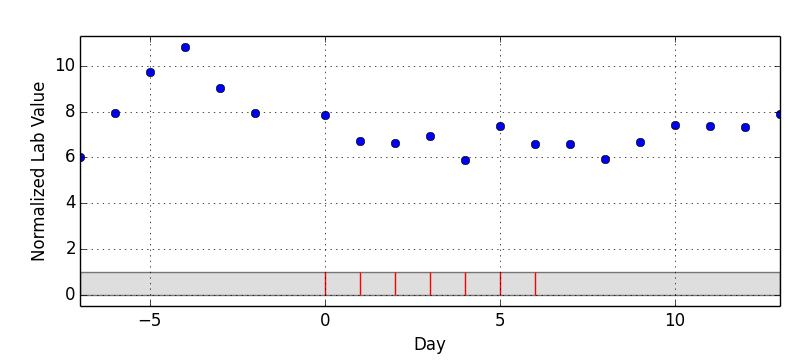

Supplement: S2 File — The “Curve Assessment Tool” (CAT) software application. This archive also contains the plots of all curves in Portable Network Graphics (PNG) format. (ZIP) [file pone.0136131.s002.zip › data/126.png]

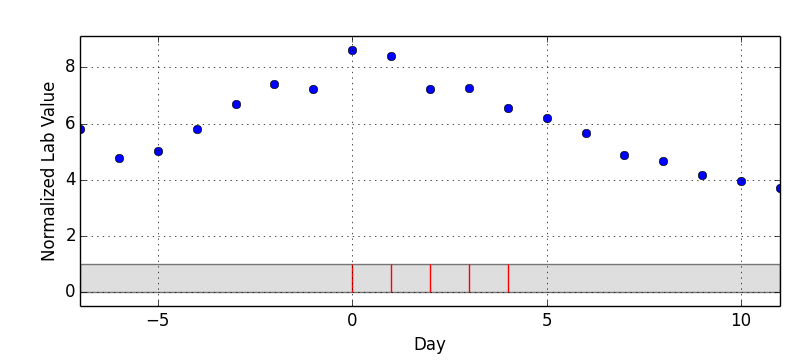

Supplement: S2 File — The “Curve Assessment Tool” (CAT) software application. This archive also contains the plots of all curves in Portable Network Graphics (PNG) format. (ZIP) [file pone.0136131.s002.zip › data/127.png]

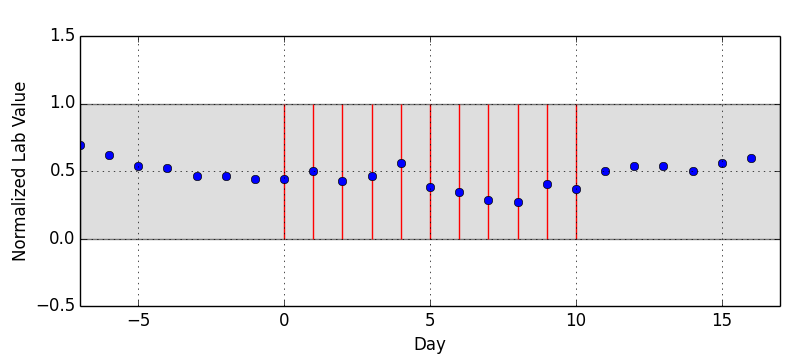

Supplement: S2 File — The “Curve Assessment Tool” (CAT) software application. This archive also contains the plots of all curves in Portable Network Graphics (PNG) format. (ZIP) [file pone.0136131.s002.zip › data/128.png]

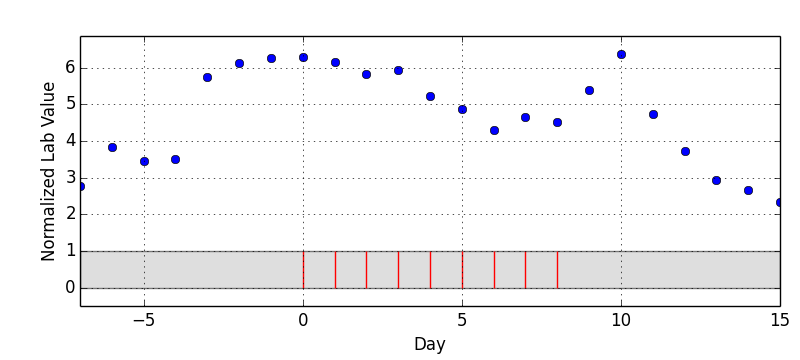

Supplement: S2 File — The “Curve Assessment Tool” (CAT) software application. This archive also contains the plots of all curves in Portable Network Graphics (PNG) format. (ZIP) [file pone.0136131.s002.zip › data/129.png]

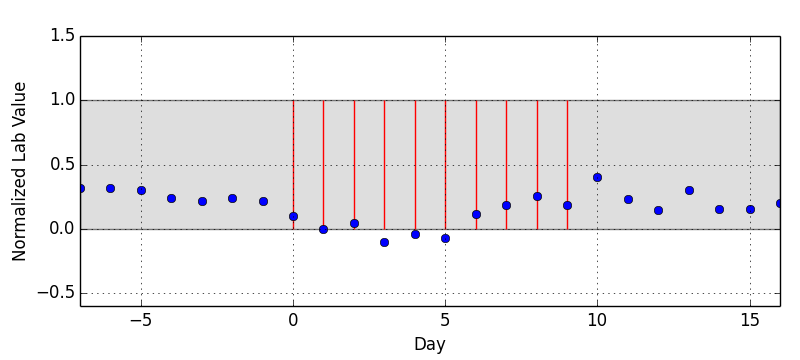

Supplement: S2 File — The “Curve Assessment Tool” (CAT) software application. This archive also contains the plots of all curves in Portable Network Graphics (PNG) format. (ZIP) [file pone.0136131.s002.zip › data/130.png]

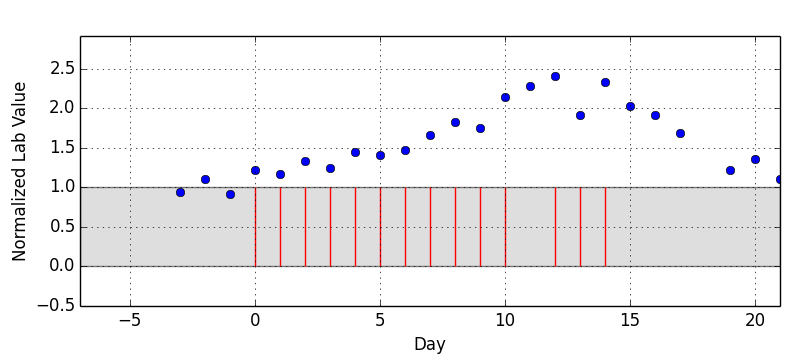

Supplement: S2 File — The “Curve Assessment Tool” (CAT) software application. This archive also contains the plots of all curves in Portable Network Graphics (PNG) format. (ZIP) [file pone.0136131.s002.zip › data/131.png]

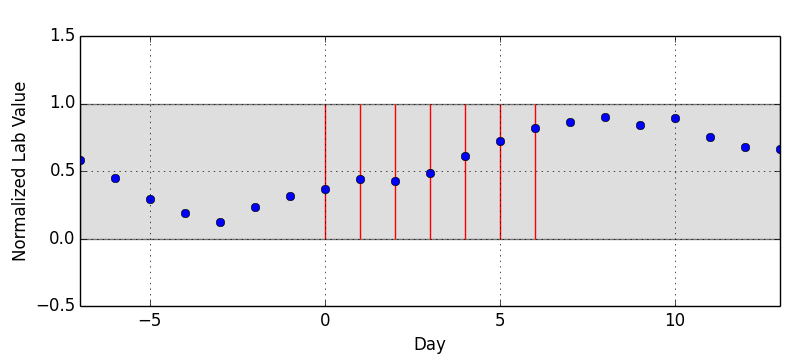

Supplement: S2 File — The “Curve Assessment Tool” (CAT) software application. This archive also contains the plots of all curves in Portable Network Graphics (PNG) format. (ZIP) [file pone.0136131.s002.zip › data/132.png]

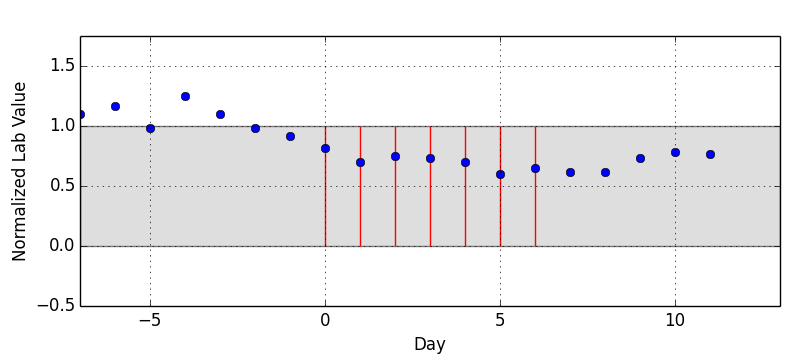

Supplement: S2 File — The “Curve Assessment Tool” (CAT) software application. This archive also contains the plots of all curves in Portable Network Graphics (PNG) format. (ZIP) [file pone.0136131.s002.zip › data/133.png]

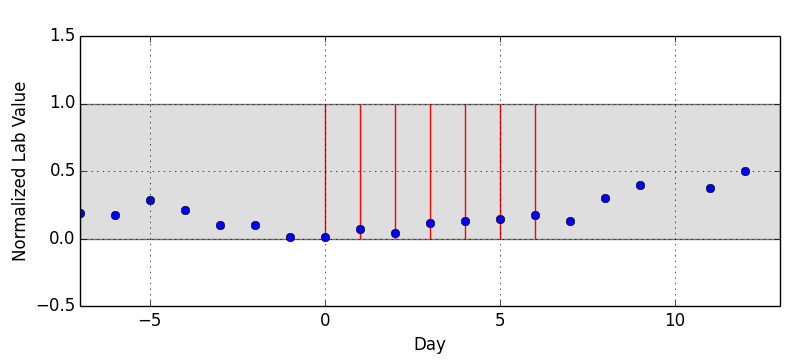

Supplement: S2 File — The “Curve Assessment Tool” (CAT) software application. This archive also contains the plots of all curves in Portable Network Graphics (PNG) format. (ZIP) [file pone.0136131.s002.zip › data/134.png]

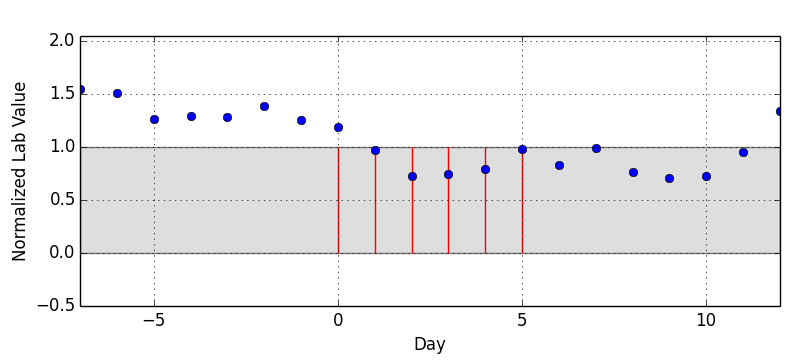

Supplement: S2 File — The “Curve Assessment Tool” (CAT) software application. This archive also contains the plots of all curves in Portable Network Graphics (PNG) format. (ZIP) [file pone.0136131.s002.zip › data/135.png]

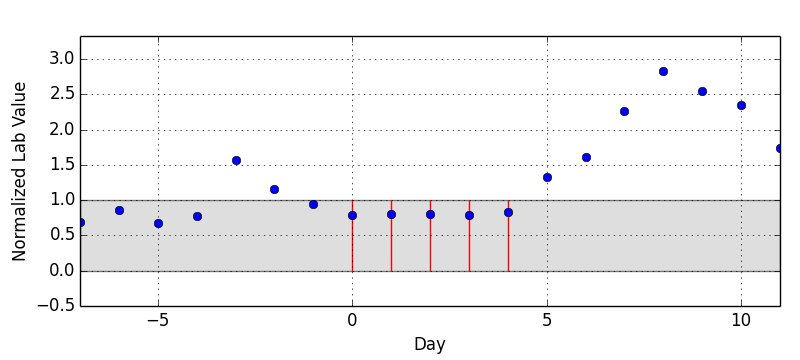

Supplement: S2 File — The “Curve Assessment Tool” (CAT) software application. This archive also contains the plots of all curves in Portable Network Graphics (PNG) format. (ZIP) [file pone.0136131.s002.zip › data/136.png]

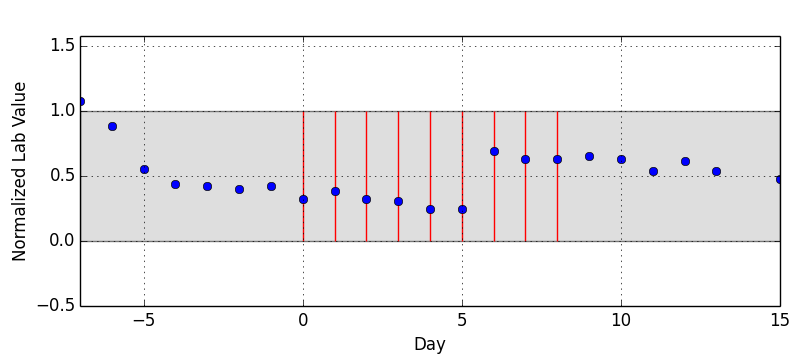

Supplement: S2 File — The “Curve Assessment Tool” (CAT) software application. This archive also contains the plots of all curves in Portable Network Graphics (PNG) format. (ZIP) [file pone.0136131.s002.zip › data/137.png]

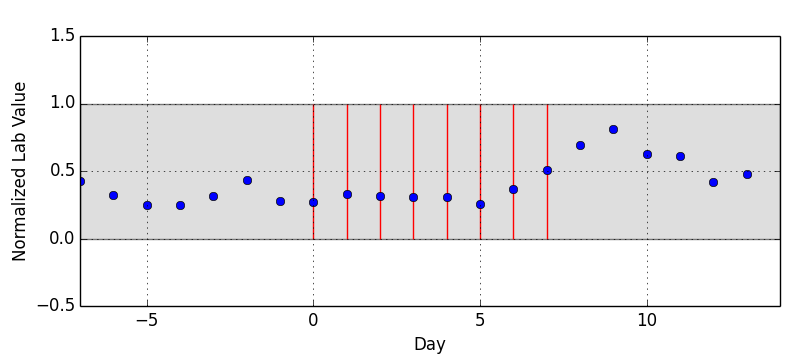

Supplement: S2 File — The “Curve Assessment Tool” (CAT) software application. This archive also contains the plots of all curves in Portable Network Graphics (PNG) format. (ZIP) [file pone.0136131.s002.zip › data/138.png]

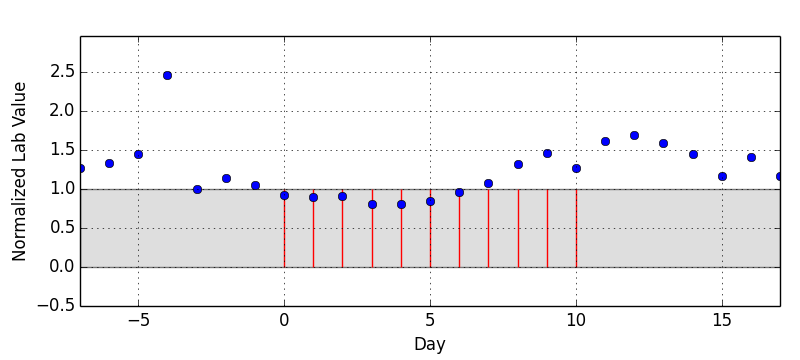

Supplement: S2 File — The “Curve Assessment Tool” (CAT) software application. This archive also contains the plots of all curves in Portable Network Graphics (PNG) format. (ZIP) [file pone.0136131.s002.zip › data/139.png]

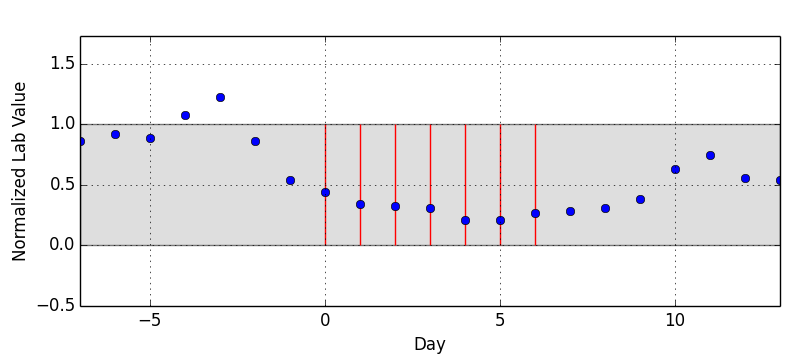

Supplement: S2 File — The “Curve Assessment Tool” (CAT) software application. This archive also contains the plots of all curves in Portable Network Graphics (PNG) format. (ZIP) [file pone.0136131.s002.zip › data/140.png]

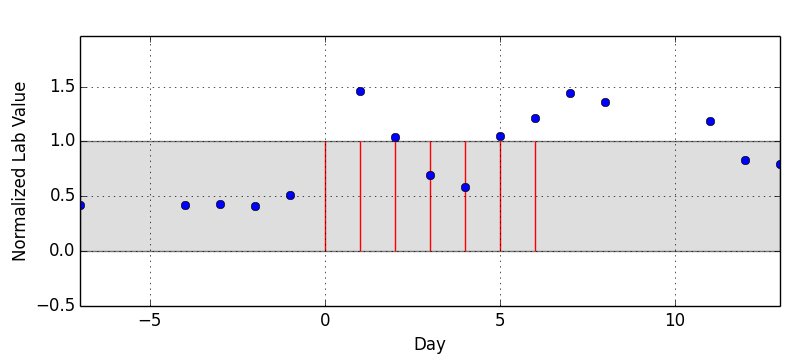

Supplement: S2 File — The “Curve Assessment Tool” (CAT) software application. This archive also contains the plots of all curves in Portable Network Graphics (PNG) format. (ZIP) [file pone.0136131.s002.zip › data/141.png]

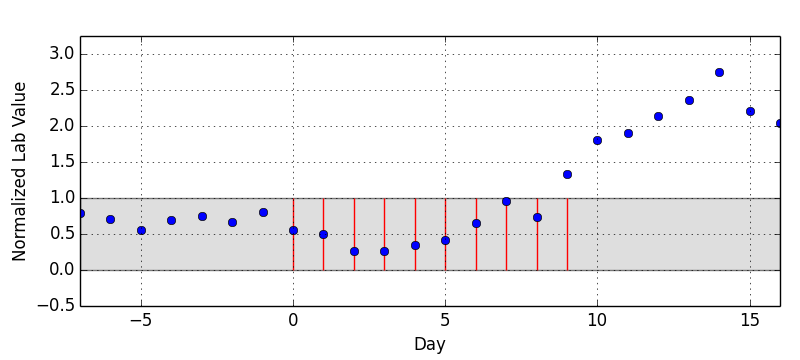

Supplement: S2 File — The “Curve Assessment Tool” (CAT) software application. This archive also contains the plots of all curves in Portable Network Graphics (PNG) format. (ZIP) [file pone.0136131.s002.zip › data/142.png]

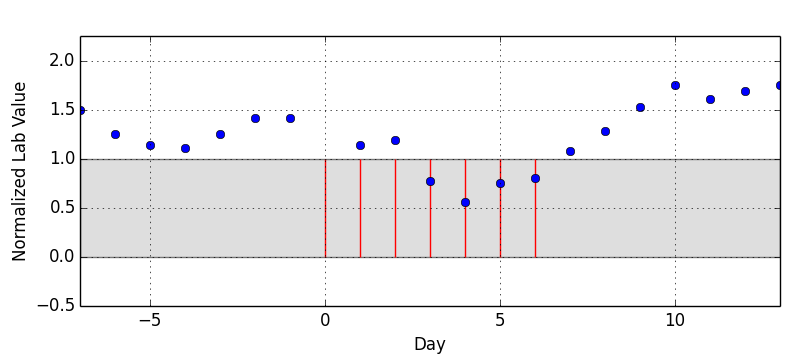

Supplement: S2 File — The “Curve Assessment Tool” (CAT) software application. This archive also contains the plots of all curves in Portable Network Graphics (PNG) format. (ZIP) [file pone.0136131.s002.zip › data/143.png]

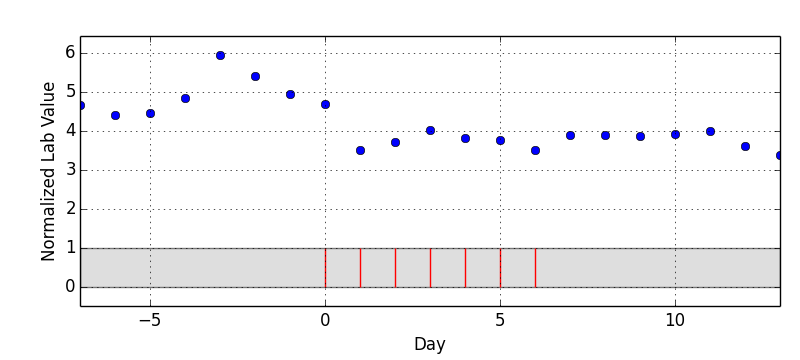

Supplement: S2 File — The “Curve Assessment Tool” (CAT) software application. This archive also contains the plots of all curves in Portable Network Graphics (PNG) format. (ZIP) [file pone.0136131.s002.zip › data/144.png]

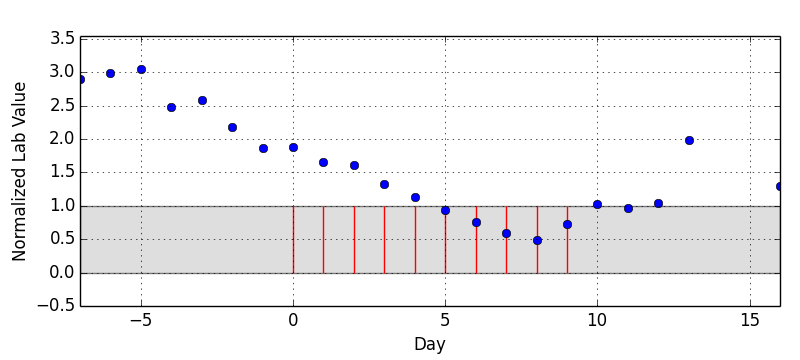

Supplement: S2 File — The “Curve Assessment Tool” (CAT) software application. This archive also contains the plots of all curves in Portable Network Graphics (PNG) format. (ZIP) [file pone.0136131.s002.zip › data/145.png]

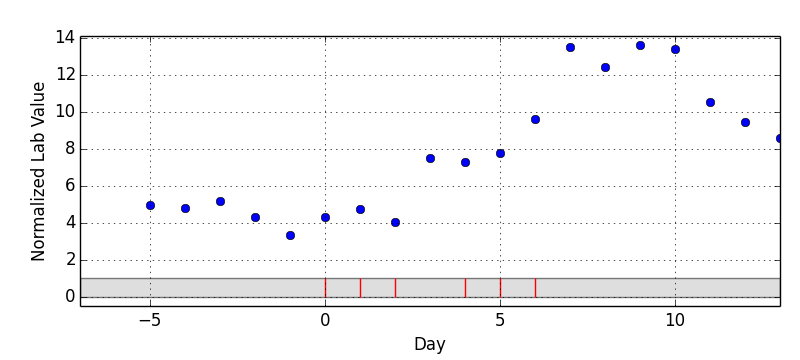

Supplement: S2 File — The “Curve Assessment Tool” (CAT) software application. This archive also contains the plots of all curves in Portable Network Graphics (PNG) format. (ZIP) [file pone.0136131.s002.zip › data/146.png]

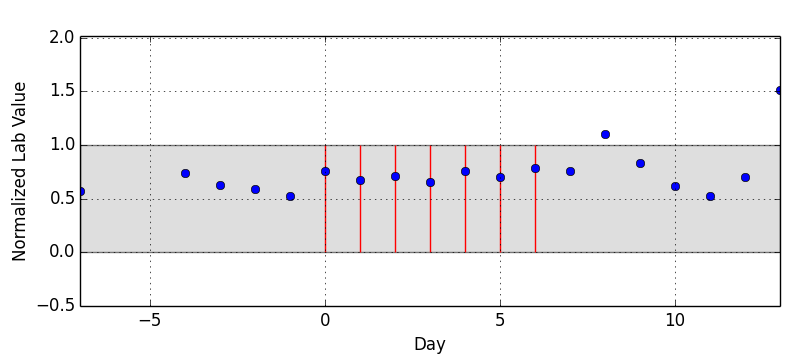

Supplement: S2 File — The “Curve Assessment Tool” (CAT) software application. This archive also contains the plots of all curves in Portable Network Graphics (PNG) format. (ZIP) [file pone.0136131.s002.zip › data/147.png]

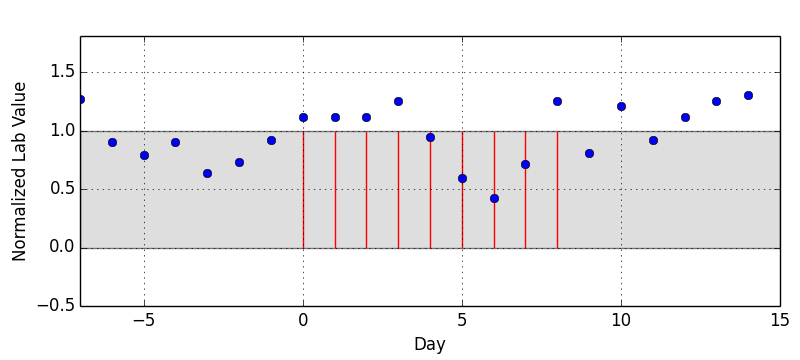

Supplement: S2 File — The “Curve Assessment Tool” (CAT) software application. This archive also contains the plots of all curves in Portable Network Graphics (PNG) format. (ZIP) [file pone.0136131.s002.zip › data/148.png]

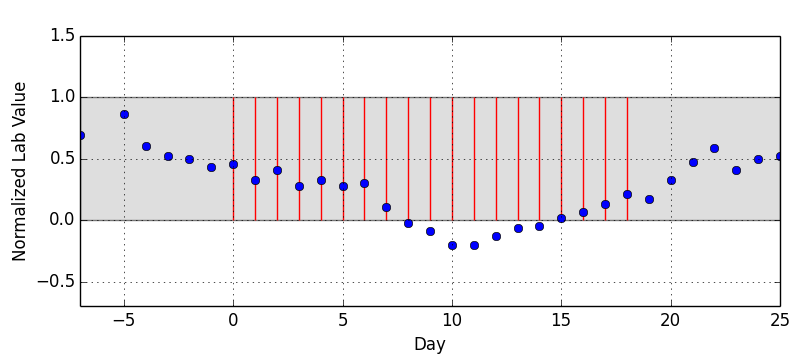

Supplement: S2 File — The “Curve Assessment Tool” (CAT) software application. This archive also contains the plots of all curves in Portable Network Graphics (PNG) format. (ZIP) [file pone.0136131.s002.zip › data/149.png]

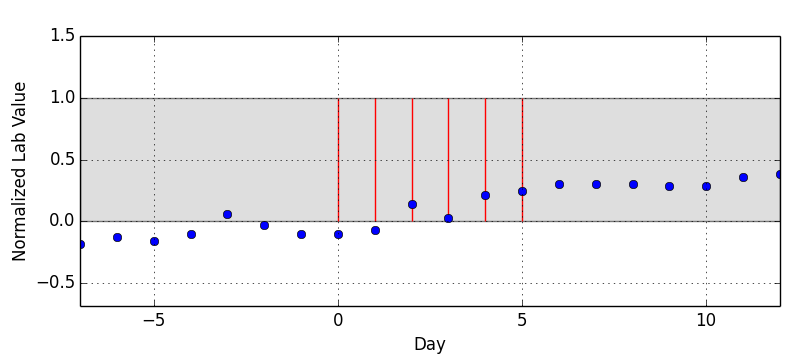

Supplement: S2 File — The “Curve Assessment Tool” (CAT) software application. This archive also contains the plots of all curves in Portable Network Graphics (PNG) format. (ZIP) [file pone.0136131.s002.zip › data/150.png]

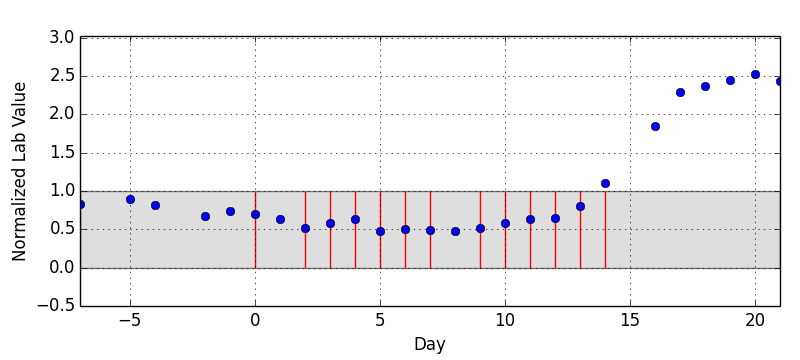

Supplement: S2 File — The “Curve Assessment Tool” (CAT) software application. This archive also contains the plots of all curves in Portable Network Graphics (PNG) format. (ZIP) [file pone.0136131.s002.zip › data/151.png]

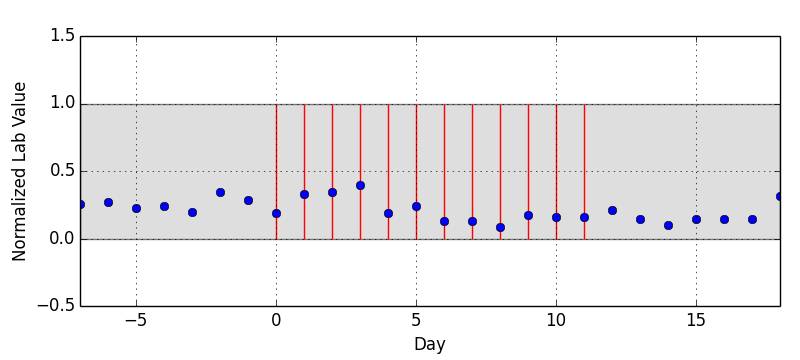

Supplement: S2 File — The “Curve Assessment Tool” (CAT) software application. This archive also contains the plots of all curves in Portable Network Graphics (PNG) format. (ZIP) [file pone.0136131.s002.zip › data/152.png]

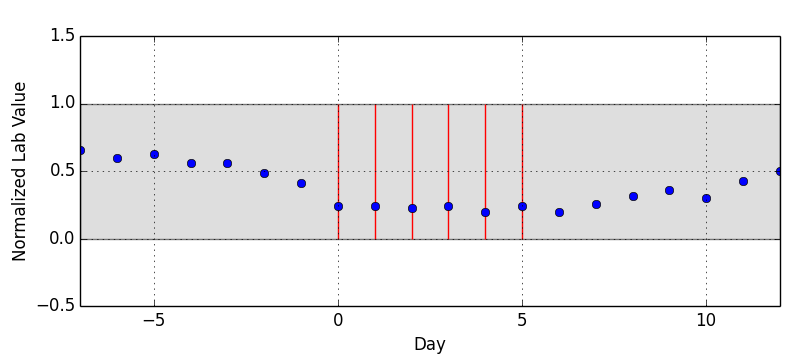

Supplement: S2 File — The “Curve Assessment Tool” (CAT) software application. This archive also contains the plots of all curves in Portable Network Graphics (PNG) format. (ZIP) [file pone.0136131.s002.zip › data/153.png]

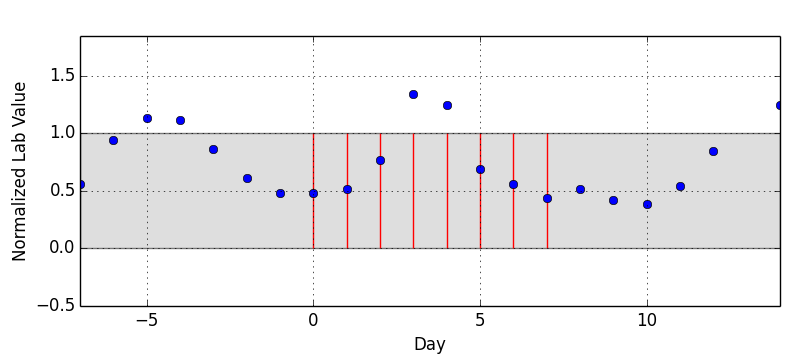

Supplement: S2 File — The “Curve Assessment Tool” (CAT) software application. This archive also contains the plots of all curves in Portable Network Graphics (PNG) format. (ZIP) [file pone.0136131.s002.zip › data/154.png]

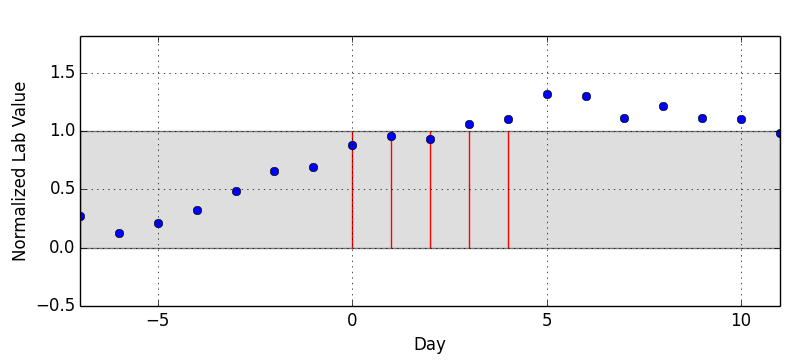

Supplement: S2 File — The “Curve Assessment Tool” (CAT) software application. This archive also contains the plots of all curves in Portable Network Graphics (PNG) format. (ZIP) [file pone.0136131.s002.zip › data/155.png]

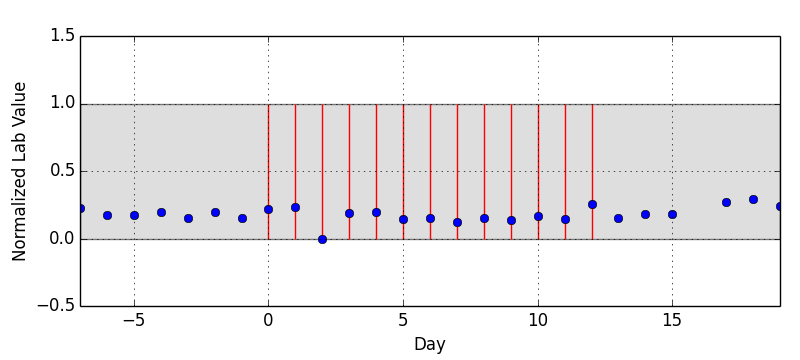

Supplement: S2 File — The “Curve Assessment Tool” (CAT) software application. This archive also contains the plots of all curves in Portable Network Graphics (PNG) format. (ZIP) [file pone.0136131.s002.zip › data/156.png]

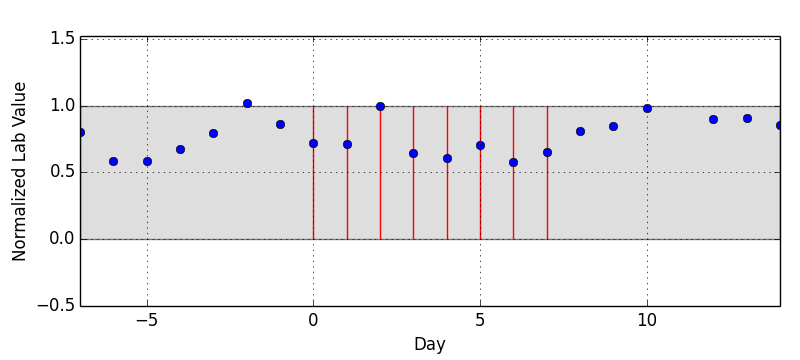

Supplement: S2 File — The “Curve Assessment Tool” (CAT) software application. This archive also contains the plots of all curves in Portable Network Graphics (PNG) format. (ZIP) [file pone.0136131.s002.zip › data/157.png]

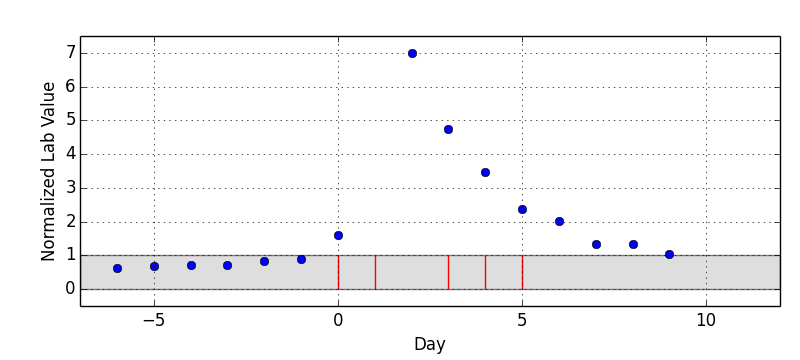

Supplement: S2 File — The “Curve Assessment Tool” (CAT) software application. This archive also contains the plots of all curves in Portable Network Graphics (PNG) format. (ZIP) [file pone.0136131.s002.zip › data/158.png]

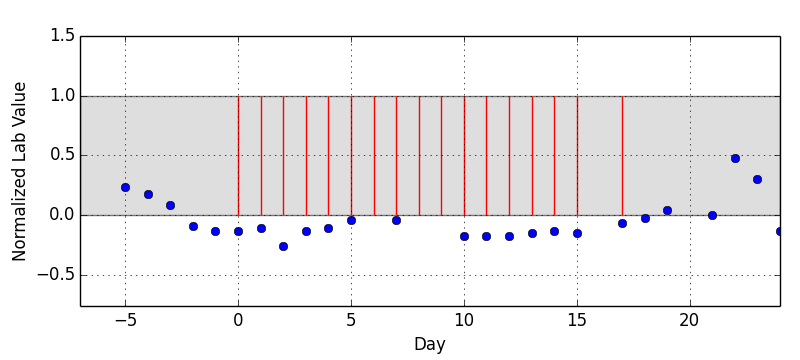

Supplement: S2 File — The “Curve Assessment Tool” (CAT) software application. This archive also contains the plots of all curves in Portable Network Graphics (PNG) format. (ZIP) [file pone.0136131.s002.zip › data/159.png]

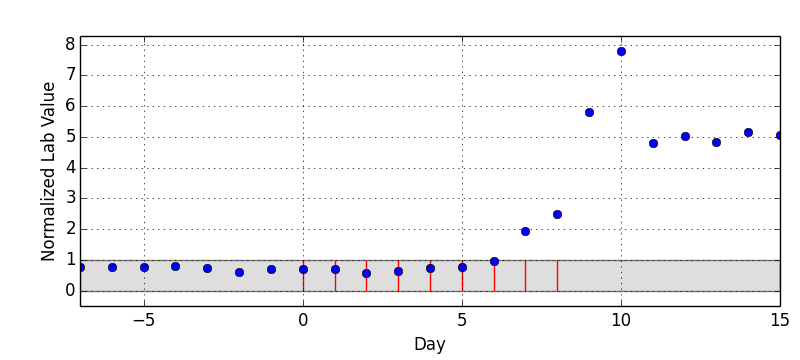

Supplement: S2 File — The “Curve Assessment Tool” (CAT) software application. This archive also contains the plots of all curves in Portable Network Graphics (PNG) format. (ZIP) [file pone.0136131.s002.zip › data/160.png]

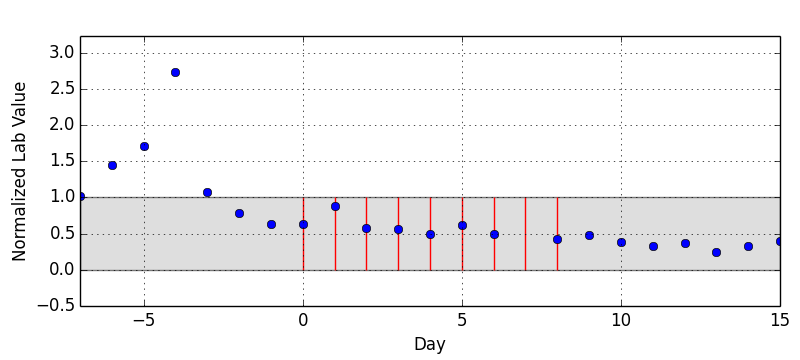

Supplement: S2 File — The “Curve Assessment Tool” (CAT) software application. This archive also contains the plots of all curves in Portable Network Graphics (PNG) format. (ZIP) [file pone.0136131.s002.zip › data/161.png]

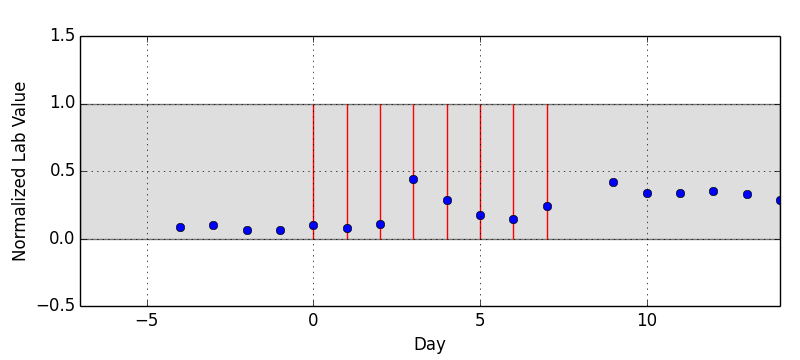

Supplement: S2 File — The “Curve Assessment Tool” (CAT) software application. This archive also contains the plots of all curves in Portable Network Graphics (PNG) format. (ZIP) [file pone.0136131.s002.zip › data/162.png]

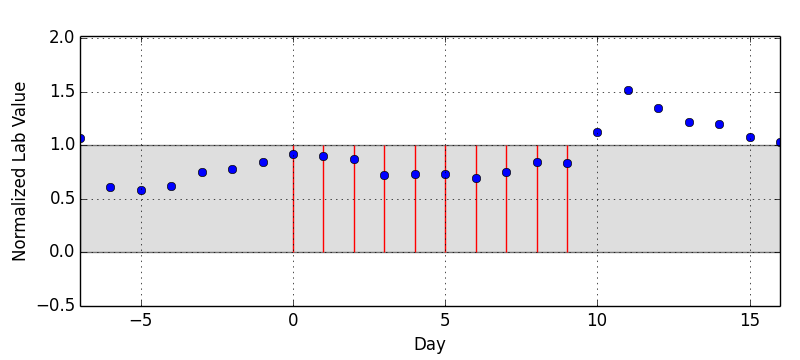

Supplement: S2 File — The “Curve Assessment Tool” (CAT) software application. This archive also contains the plots of all curves in Portable Network Graphics (PNG) format. (ZIP) [file pone.0136131.s002.zip › data/163.png]

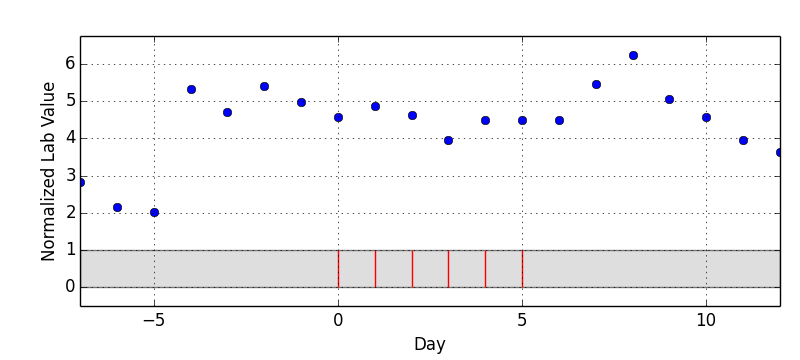

Supplement: S2 File — The “Curve Assessment Tool” (CAT) software application. This archive also contains the plots of all curves in Portable Network Graphics (PNG) format. (ZIP) [file pone.0136131.s002.zip › data/164.png]

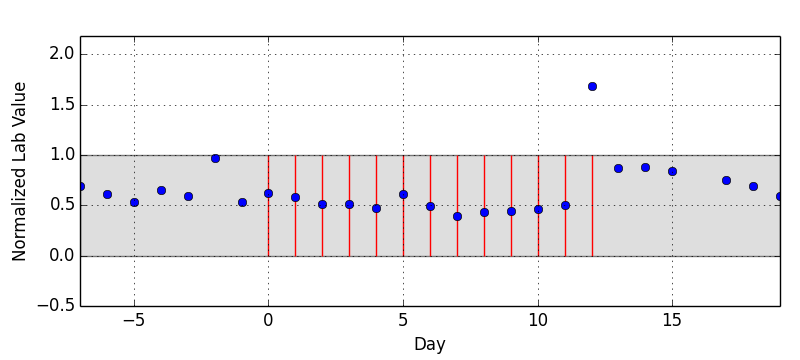

Supplement: S2 File — The “Curve Assessment Tool” (CAT) software application. This archive also contains the plots of all curves in Portable Network Graphics (PNG) format. (ZIP) [file pone.0136131.s002.zip › data/165.png]

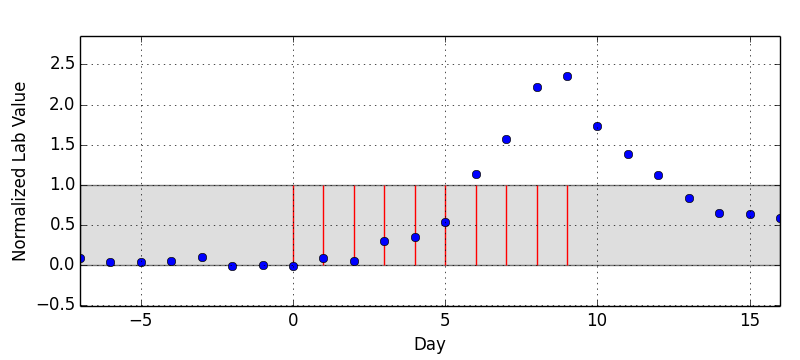

Supplement: S2 File — The “Curve Assessment Tool” (CAT) software application. This archive also contains the plots of all curves in Portable Network Graphics (PNG) format. (ZIP) [file pone.0136131.s002.zip › data/166.png]

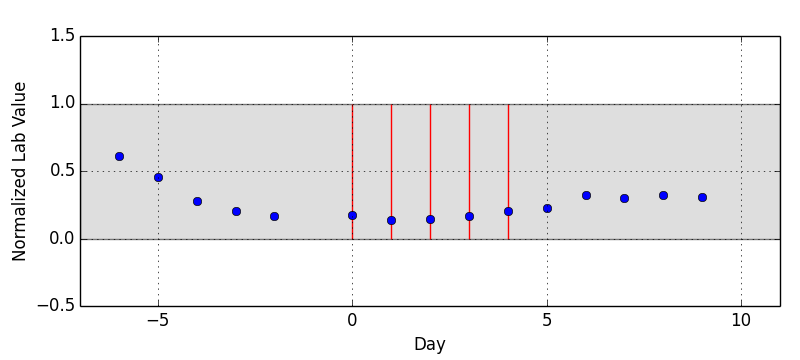

Supplement: S2 File — The “Curve Assessment Tool” (CAT) software application. This archive also contains the plots of all curves in Portable Network Graphics (PNG) format. (ZIP) [file pone.0136131.s002.zip › data/167.png]

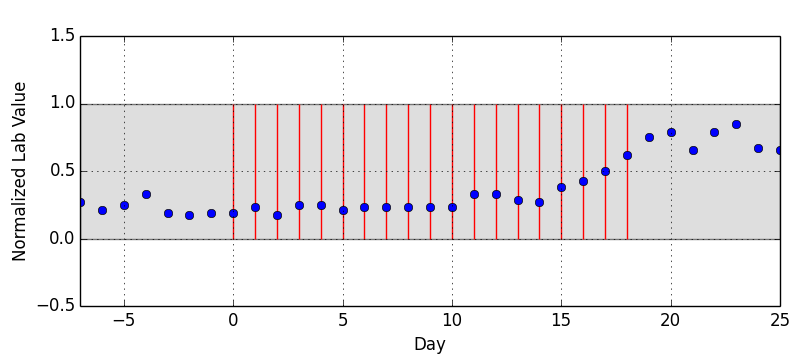

Supplement: S2 File — The “Curve Assessment Tool” (CAT) software application. This archive also contains the plots of all curves in Portable Network Graphics (PNG) format. (ZIP) [file pone.0136131.s002.zip › data/168.png]

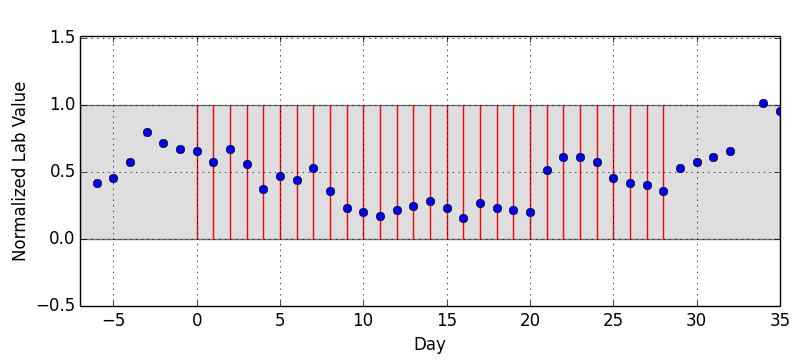

Supplement: S2 File — The “Curve Assessment Tool” (CAT) software application. This archive also contains the plots of all curves in Portable Network Graphics (PNG) format. (ZIP) [file pone.0136131.s002.zip › data/169.png]

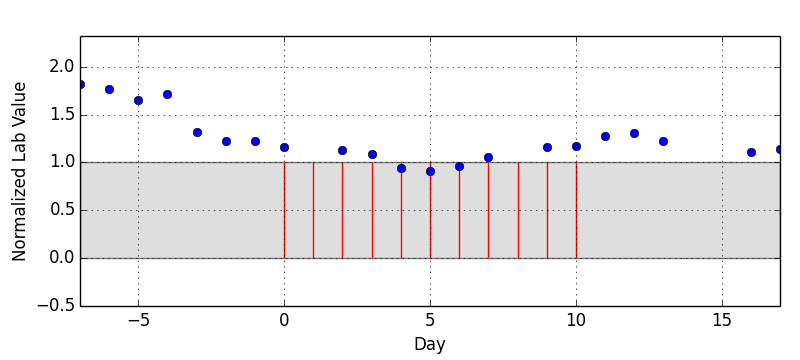

Supplement: S2 File — The “Curve Assessment Tool” (CAT) software application. This archive also contains the plots of all curves in Portable Network Graphics (PNG) format. (ZIP) [file pone.0136131.s002.zip › data/170.png]

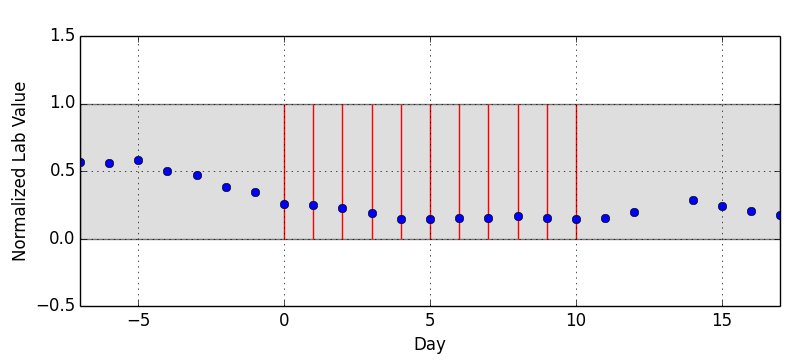

Supplement: S2 File — The “Curve Assessment Tool” (CAT) software application. This archive also contains the plots of all curves in Portable Network Graphics (PNG) format. (ZIP) [file pone.0136131.s002.zip › data/171.png]

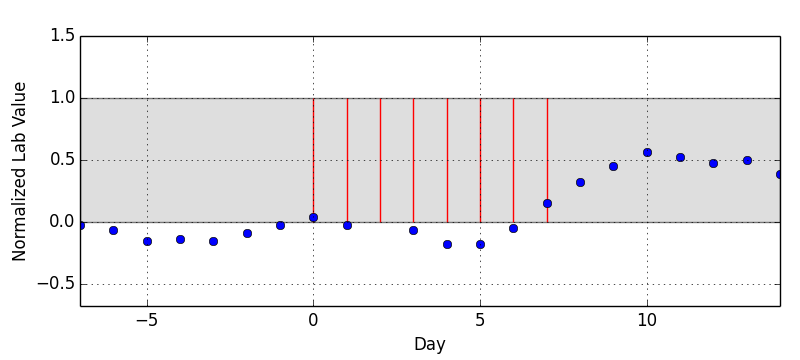

Supplement: S2 File — The “Curve Assessment Tool” (CAT) software application. This archive also contains the plots of all curves in Portable Network Graphics (PNG) format. (ZIP) [file pone.0136131.s002.zip › data/172.png]

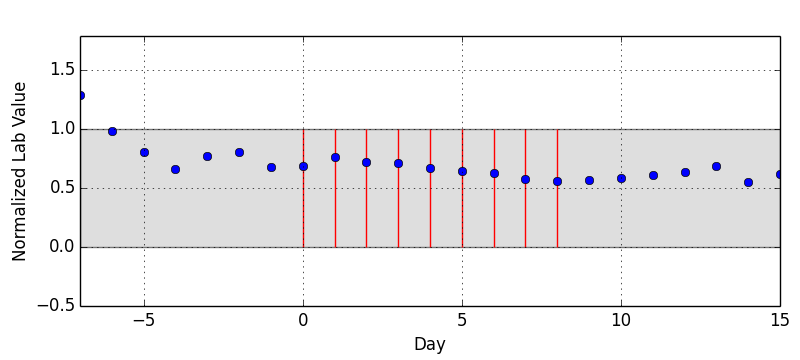

Supplement: S2 File — The “Curve Assessment Tool” (CAT) software application. This archive also contains the plots of all curves in Portable Network Graphics (PNG) format. (ZIP) [file pone.0136131.s002.zip › data/173.png]

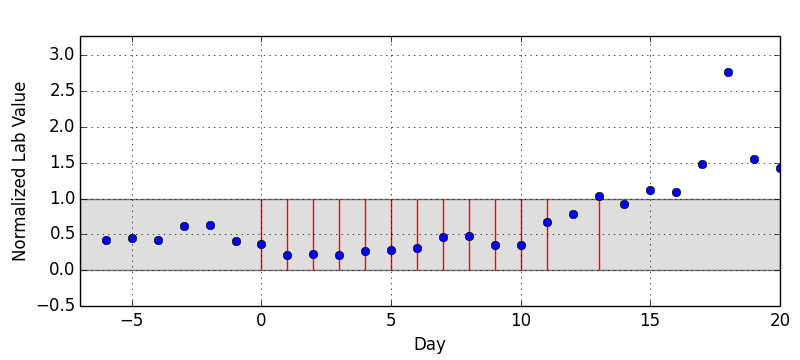

Supplement: S2 File — The “Curve Assessment Tool” (CAT) software application. This archive also contains the plots of all curves in Portable Network Graphics (PNG) format. (ZIP) [file pone.0136131.s002.zip › data/174.png]

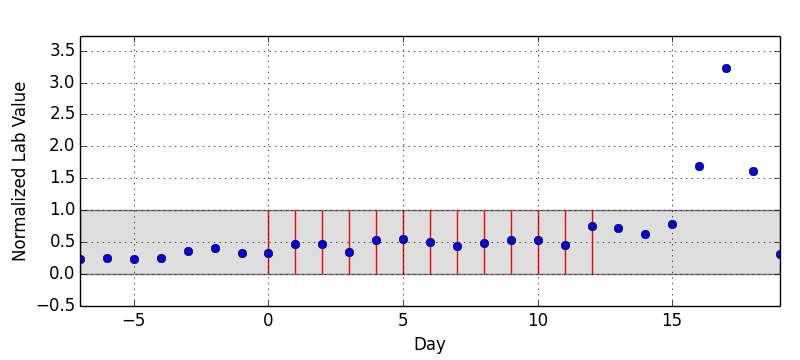

Supplement: S2 File — The “Curve Assessment Tool” (CAT) software application. This archive also contains the plots of all curves in Portable Network Graphics (PNG) format. (ZIP) [file pone.0136131.s002.zip › data/175.png]

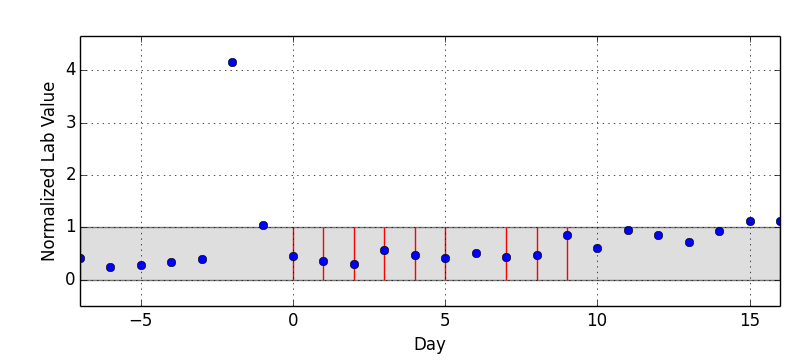

Supplement: S2 File — The “Curve Assessment Tool” (CAT) software application. This archive also contains the plots of all curves in Portable Network Graphics (PNG) format. (ZIP) [file pone.0136131.s002.zip › data/176.png]

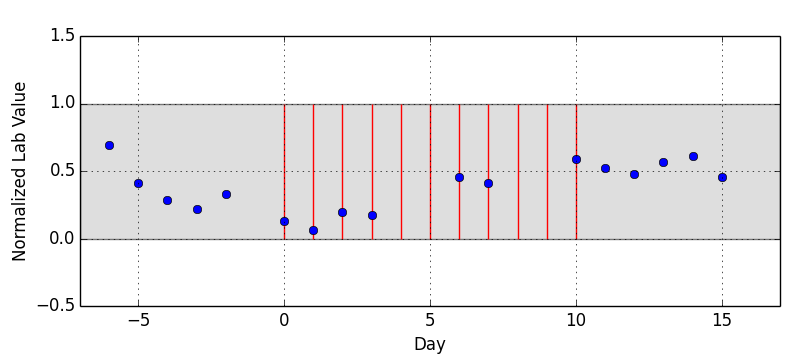

Supplement: S2 File — The “Curve Assessment Tool” (CAT) software application. This archive also contains the plots of all curves in Portable Network Graphics (PNG) format. (ZIP) [file pone.0136131.s002.zip › data/177.png]

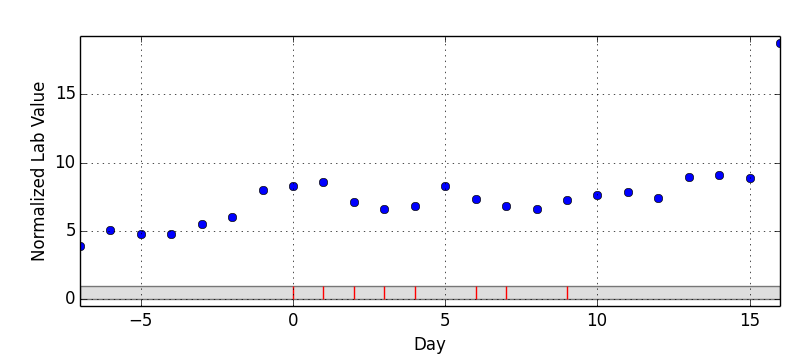

Supplement: S2 File — The “Curve Assessment Tool” (CAT) software application. This archive also contains the plots of all curves in Portable Network Graphics (PNG) format. (ZIP) [file pone.0136131.s002.zip › data/178.png]

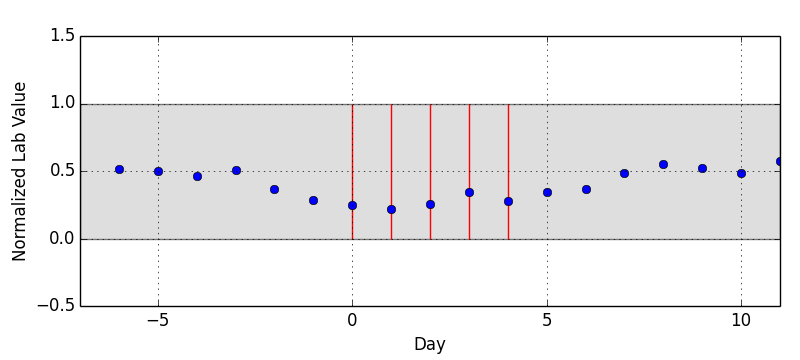

Supplement: S2 File — The “Curve Assessment Tool” (CAT) software application. This archive also contains the plots of all curves in Portable Network Graphics (PNG) format. (ZIP) [file pone.0136131.s002.zip › data/179.png]

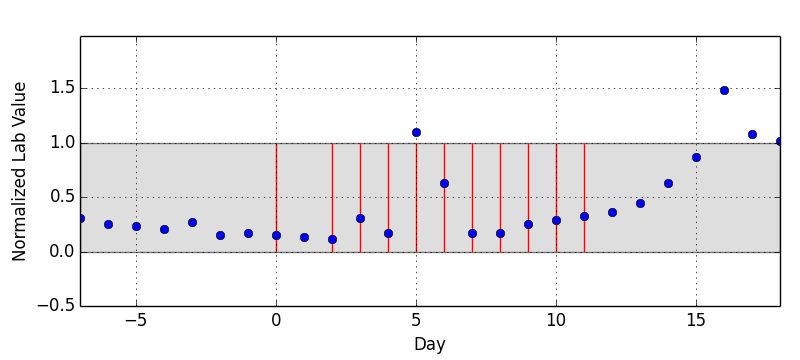

Supplement: S2 File — The “Curve Assessment Tool” (CAT) software application. This archive also contains the plots of all curves in Portable Network Graphics (PNG) format. (ZIP) [file pone.0136131.s002.zip › data/180.png]

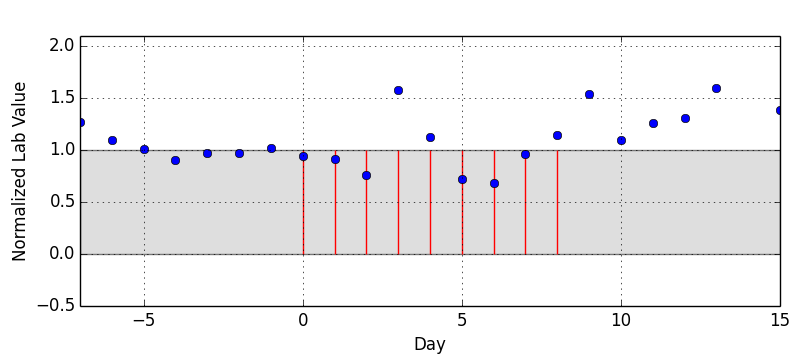

Supplement: S2 File — The “Curve Assessment Tool” (CAT) software application. This archive also contains the plots of all curves in Portable Network Graphics (PNG) format. (ZIP) [file pone.0136131.s002.zip › data/181.png]

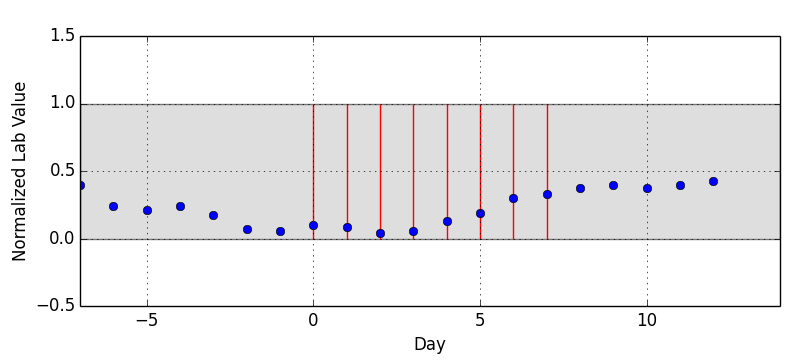

Supplement: S2 File — The “Curve Assessment Tool” (CAT) software application. This archive also contains the plots of all curves in Portable Network Graphics (PNG) format. (ZIP) [file pone.0136131.s002.zip › data/182.png]

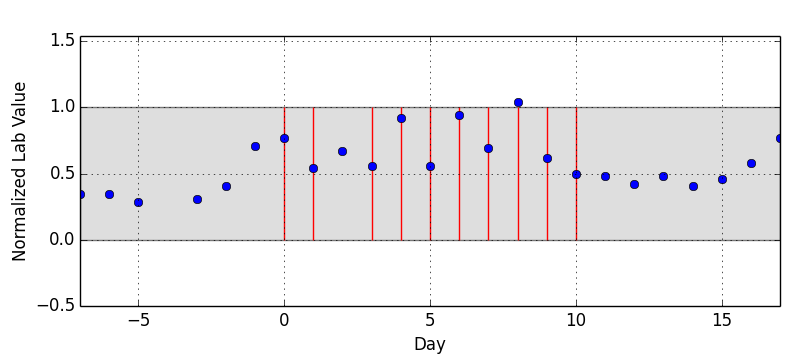

Supplement: S2 File — The “Curve Assessment Tool” (CAT) software application. This archive also contains the plots of all curves in Portable Network Graphics (PNG) format. (ZIP) [file pone.0136131.s002.zip › data/183.png]

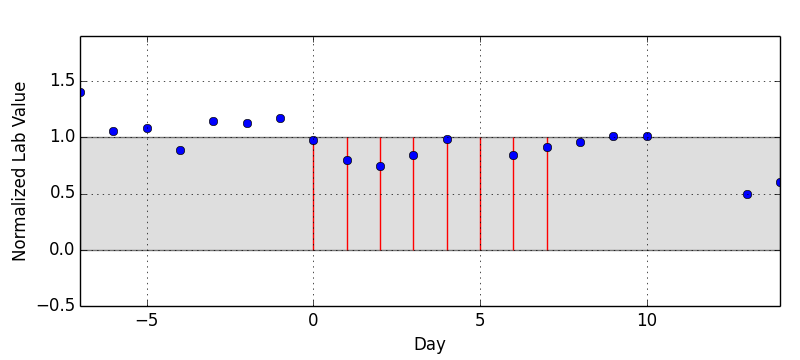

Supplement: S2 File — The “Curve Assessment Tool” (CAT) software application. This archive also contains the plots of all curves in Portable Network Graphics (PNG) format. (ZIP) [file pone.0136131.s002.zip › data/184.png]

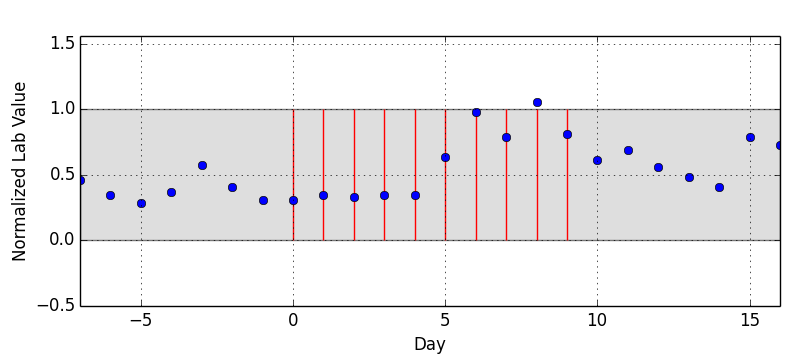

Supplement: S2 File — The “Curve Assessment Tool” (CAT) software application. This archive also contains the plots of all curves in Portable Network Graphics (PNG) format. (ZIP) [file pone.0136131.s002.zip › data/185.png]

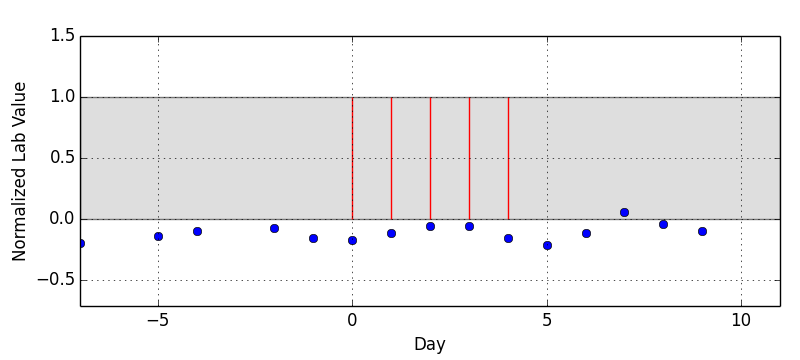

Supplement: S2 File — The “Curve Assessment Tool” (CAT) software application. This archive also contains the plots of all curves in Portable Network Graphics (PNG) format. (ZIP) [file pone.0136131.s002.zip › data/186.png]

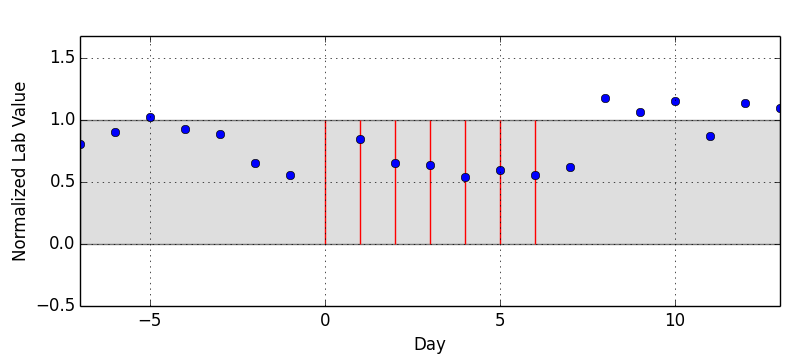

Supplement: S2 File — The “Curve Assessment Tool” (CAT) software application. This archive also contains the plots of all curves in Portable Network Graphics (PNG) format. (ZIP) [file pone.0136131.s002.zip › data/187.png]

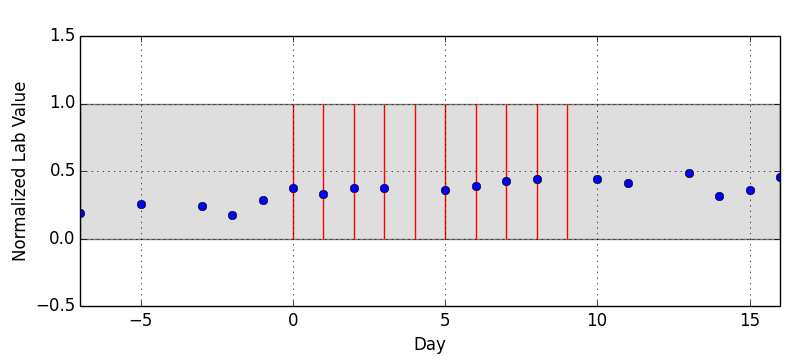

Supplement: S2 File — The “Curve Assessment Tool” (CAT) software application. This archive also contains the plots of all curves in Portable Network Graphics (PNG) format. (ZIP) [file pone.0136131.s002.zip › data/188.png]

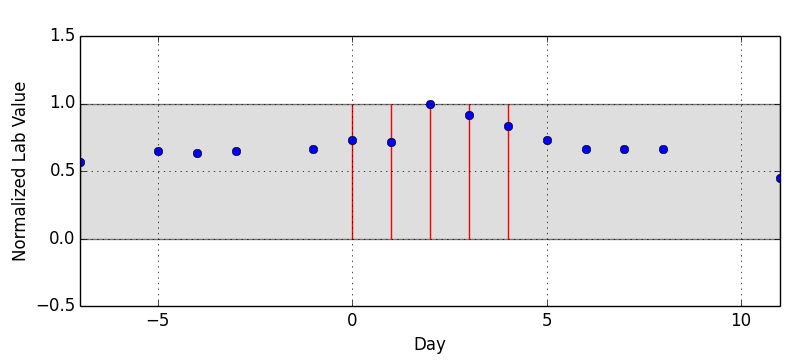

Supplement: S2 File — The “Curve Assessment Tool” (CAT) software application. This archive also contains the plots of all curves in Portable Network Graphics (PNG) format. (ZIP) [file pone.0136131.s002.zip › data/189.png]

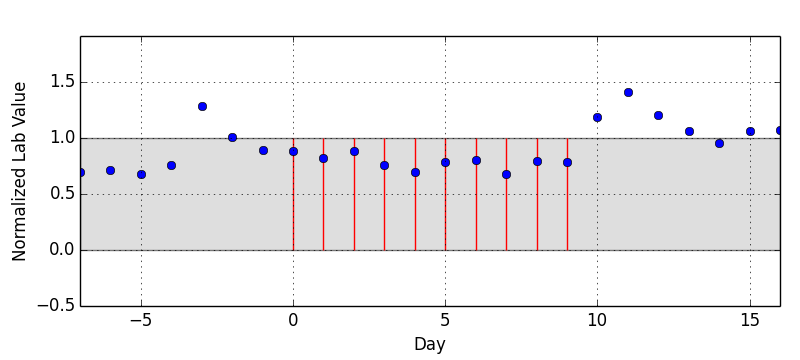

Supplement: S2 File — The “Curve Assessment Tool” (CAT) software application. This archive also contains the plots of all curves in Portable Network Graphics (PNG) format. (ZIP) [file pone.0136131.s002.zip › data/190.png]

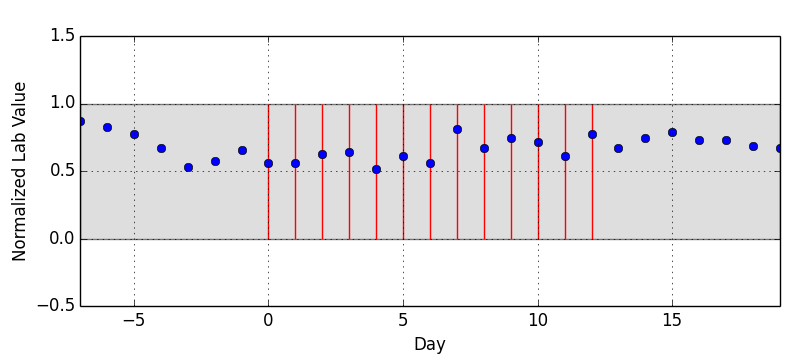

Supplement: S2 File — The “Curve Assessment Tool” (CAT) software application. This archive also contains the plots of all curves in Portable Network Graphics (PNG) format. (ZIP) [file pone.0136131.s002.zip › data/191.png]

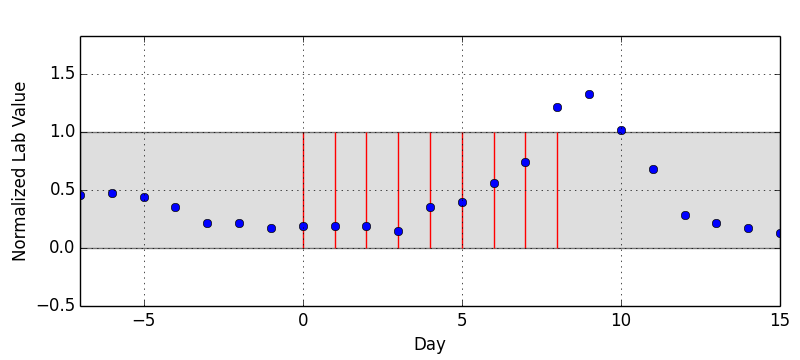

Supplement: S2 File — The “Curve Assessment Tool” (CAT) software application. This archive also contains the plots of all curves in Portable Network Graphics (PNG) format. (ZIP) [file pone.0136131.s002.zip › data/192.png]

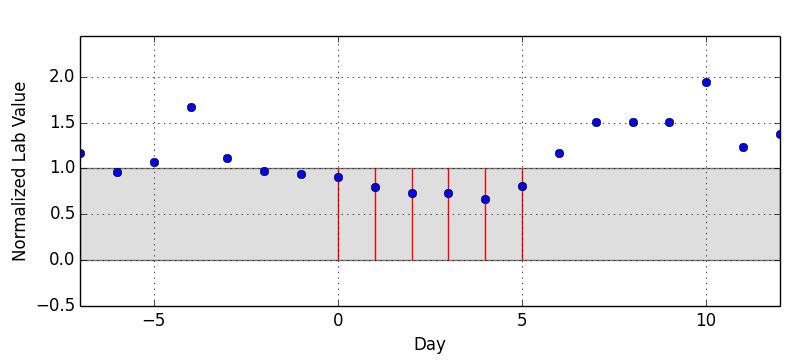

Supplement: S2 File — The “Curve Assessment Tool” (CAT) software application. This archive also contains the plots of all curves in Portable Network Graphics (PNG) format. (ZIP) [file pone.0136131.s002.zip › data/193.png]

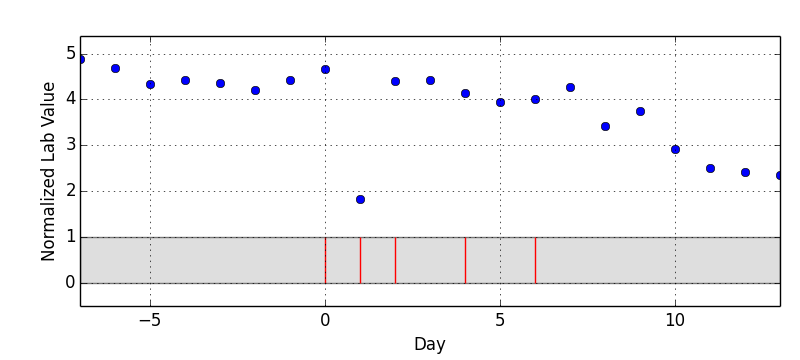

Supplement: S2 File — The “Curve Assessment Tool” (CAT) software application. This archive also contains the plots of all curves in Portable Network Graphics (PNG) format. (ZIP) [file pone.0136131.s002.zip › data/194.png]

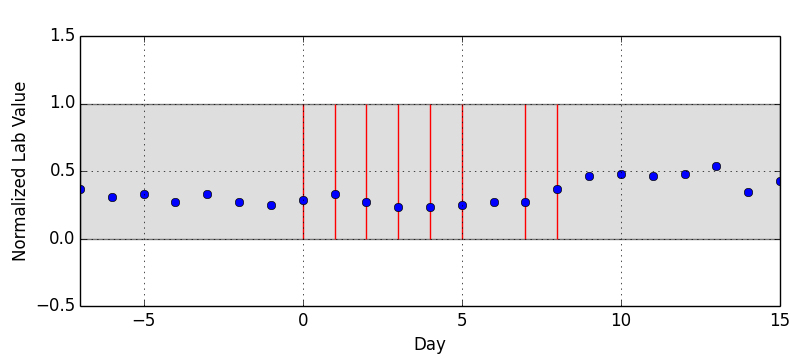

Supplement: S2 File — The “Curve Assessment Tool” (CAT) software application. This archive also contains the plots of all curves in Portable Network Graphics (PNG) format. (ZIP) [file pone.0136131.s002.zip › data/195.png]

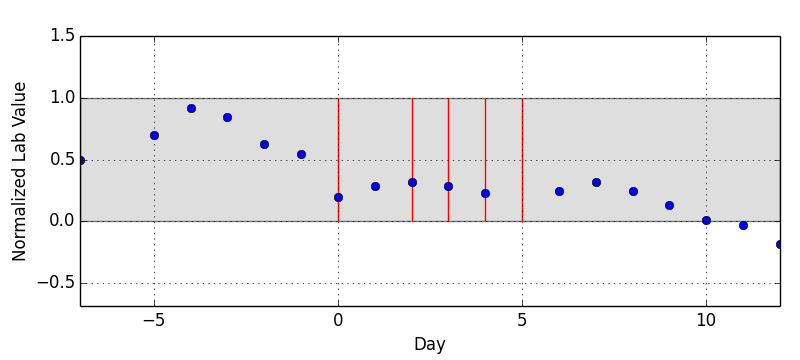

Supplement: S2 File — The “Curve Assessment Tool” (CAT) software application. This archive also contains the plots of all curves in Portable Network Graphics (PNG) format. (ZIP) [file pone.0136131.s002.zip › data/196.png]

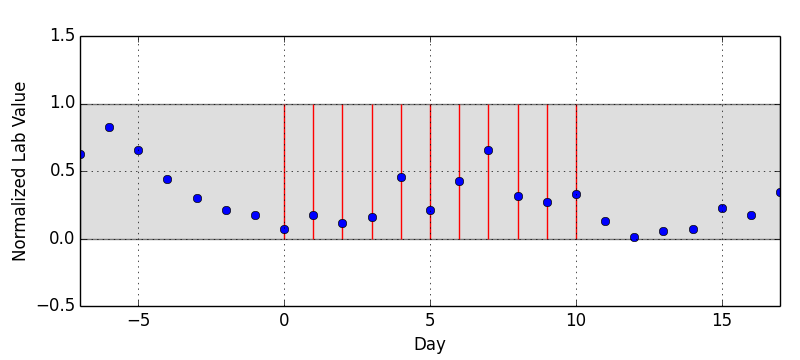

Supplement: S2 File — The “Curve Assessment Tool” (CAT) software application. This archive also contains the plots of all curves in Portable Network Graphics (PNG) format. (ZIP) [file pone.0136131.s002.zip › data/197.png]

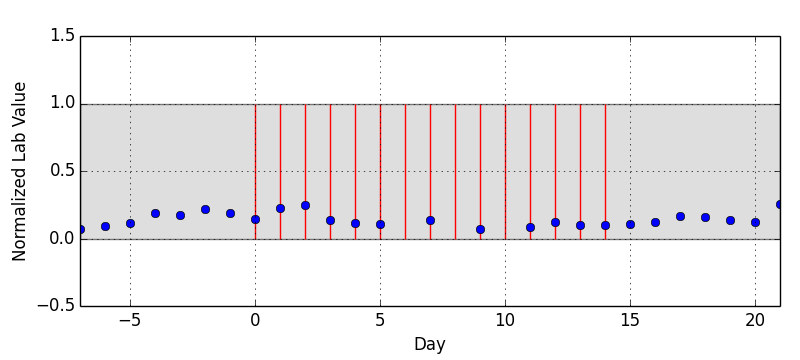

Supplement: S2 File — The “Curve Assessment Tool” (CAT) software application. This archive also contains the plots of all curves in Portable Network Graphics (PNG) format. (ZIP) [file pone.0136131.s002.zip › data/198.png]

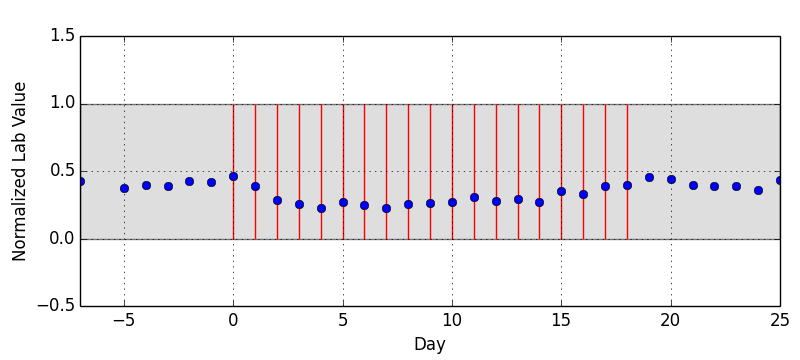

Supplement: S2 File — The “Curve Assessment Tool” (CAT) software application. This archive also contains the plots of all curves in Portable Network Graphics (PNG) format. (ZIP) [file pone.0136131.s002.zip › data/199.png]
