# Supplementary figures and images for: Towards a Computable Data Corpus of Temporal Correlations between Drug Administration and Lab Value Changes (part 1 of 2)
Source: PLoS One. 2015 Aug 24;10(8):e0136131. doi: 10.1371/journal.pone.0136131 (PMC4547740; doi:10.1371/journal.pone.0136131)

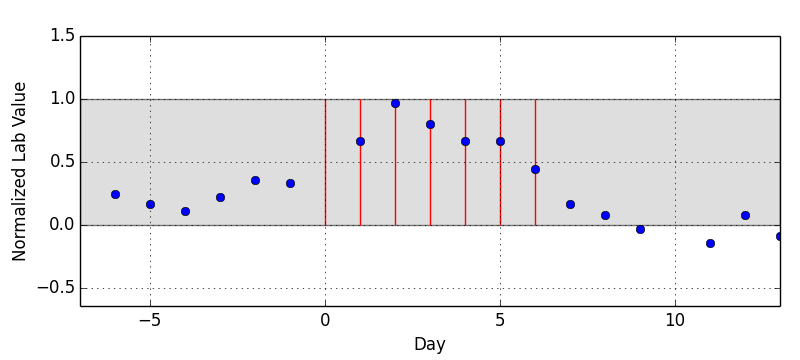

Supplement: S2 File — The “Curve Assessment Tool” (CAT) software application. This archive also contains the plots of all curves in Portable Network Graphics (PNG) format. (ZIP) [file pone.0136131.s002.zip › data/000.png]

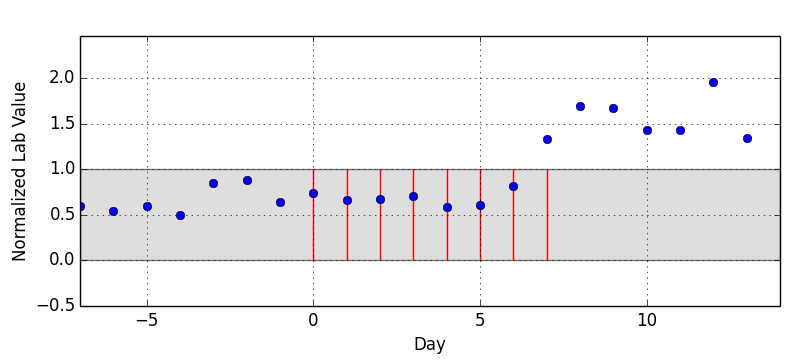

Supplement: S2 File — The “Curve Assessment Tool” (CAT) software application. This archive also contains the plots of all curves in Portable Network Graphics (PNG) format. (ZIP) [file pone.0136131.s002.zip › data/001.png]

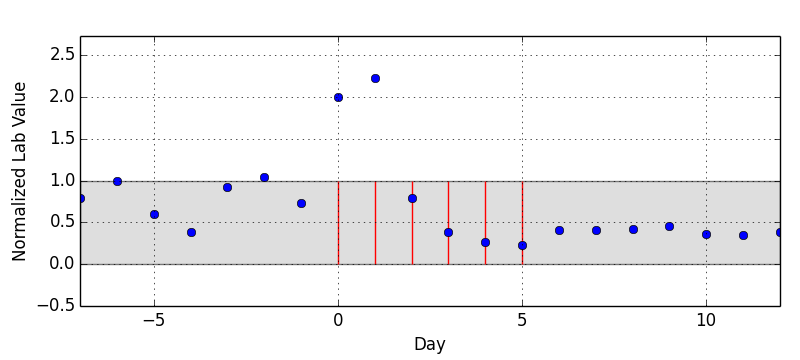

Supplement: S2 File — The “Curve Assessment Tool” (CAT) software application. This archive also contains the plots of all curves in Portable Network Graphics (PNG) format. (ZIP) [file pone.0136131.s002.zip › data/002.png]

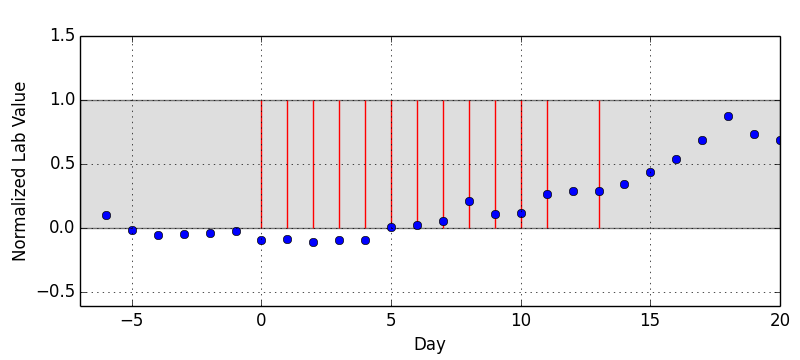

Supplement: S2 File — The “Curve Assessment Tool” (CAT) software application. This archive also contains the plots of all curves in Portable Network Graphics (PNG) format. (ZIP) [file pone.0136131.s002.zip › data/003.png]

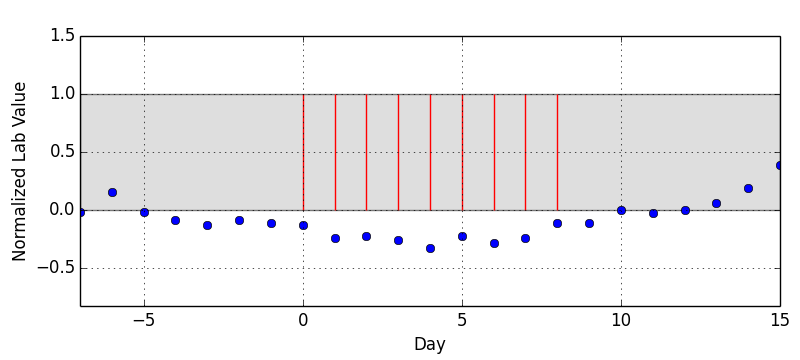

Supplement: S2 File — The “Curve Assessment Tool” (CAT) software application. This archive also contains the plots of all curves in Portable Network Graphics (PNG) format. (ZIP) [file pone.0136131.s002.zip › data/004.png]

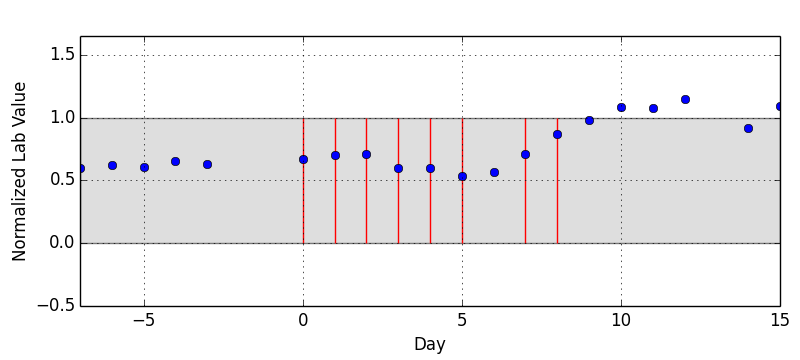

Supplement: S2 File — The “Curve Assessment Tool” (CAT) software application. This archive also contains the plots of all curves in Portable Network Graphics (PNG) format. (ZIP) [file pone.0136131.s002.zip › data/005.png]

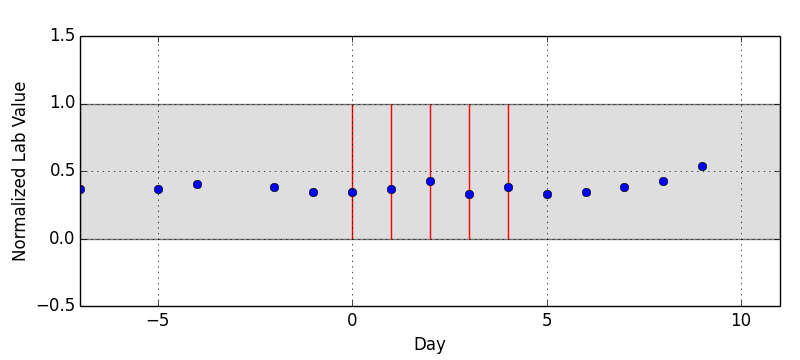

Supplement: S2 File — The “Curve Assessment Tool” (CAT) software application. This archive also contains the plots of all curves in Portable Network Graphics (PNG) format. (ZIP) [file pone.0136131.s002.zip › data/006.png]

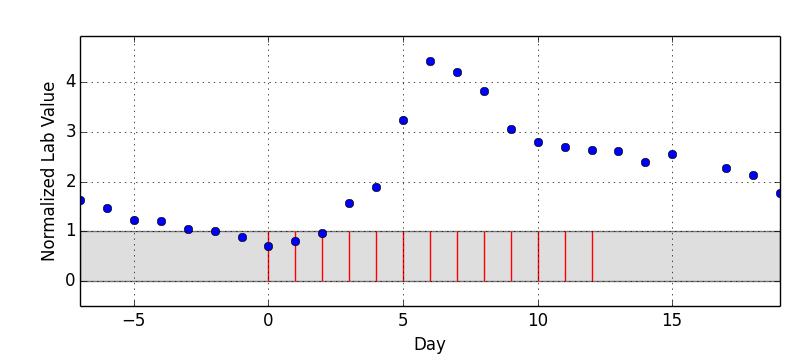

Supplement: S2 File — The “Curve Assessment Tool” (CAT) software application. This archive also contains the plots of all curves in Portable Network Graphics (PNG) format. (ZIP) [file pone.0136131.s002.zip › data/007.png]

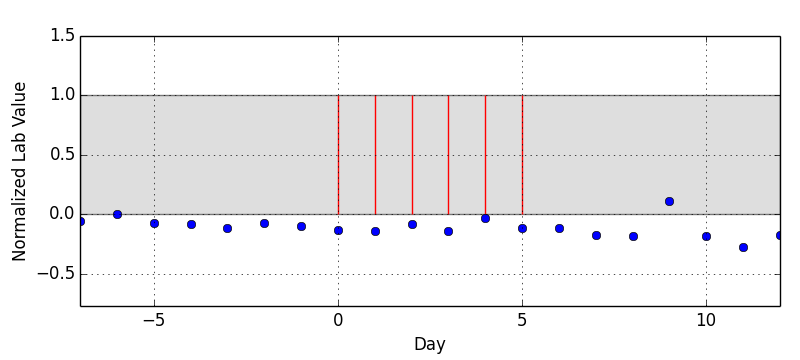

Supplement: S2 File — The “Curve Assessment Tool” (CAT) software application. This archive also contains the plots of all curves in Portable Network Graphics (PNG) format. (ZIP) [file pone.0136131.s002.zip › data/008.png]

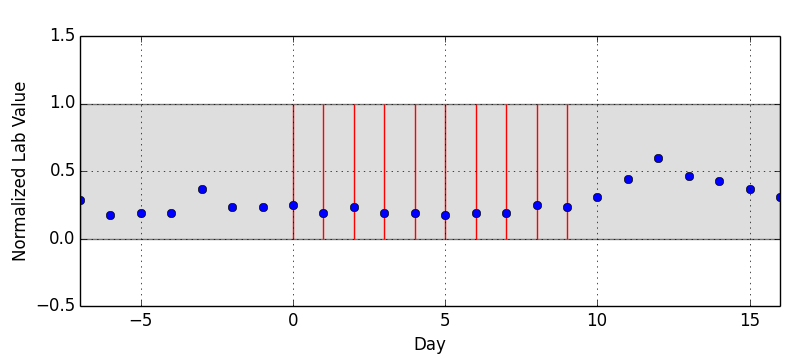

Supplement: S2 File — The “Curve Assessment Tool” (CAT) software application. This archive also contains the plots of all curves in Portable Network Graphics (PNG) format. (ZIP) [file pone.0136131.s002.zip › data/009.png]

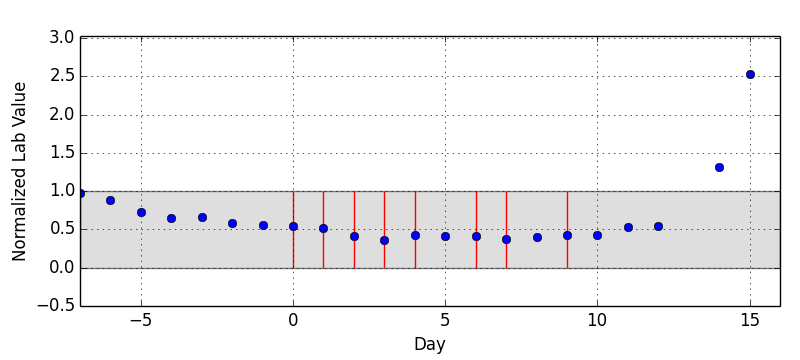

Supplement: S2 File — The “Curve Assessment Tool” (CAT) software application. This archive also contains the plots of all curves in Portable Network Graphics (PNG) format. (ZIP) [file pone.0136131.s002.zip › data/010.png]

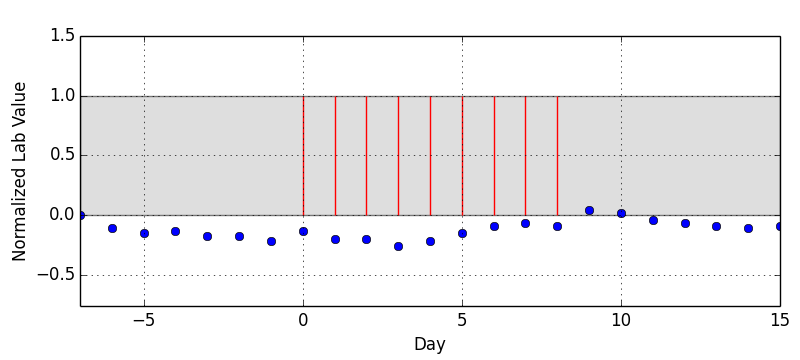

Supplement: S2 File — The “Curve Assessment Tool” (CAT) software application. This archive also contains the plots of all curves in Portable Network Graphics (PNG) format. (ZIP) [file pone.0136131.s002.zip › data/011.png]

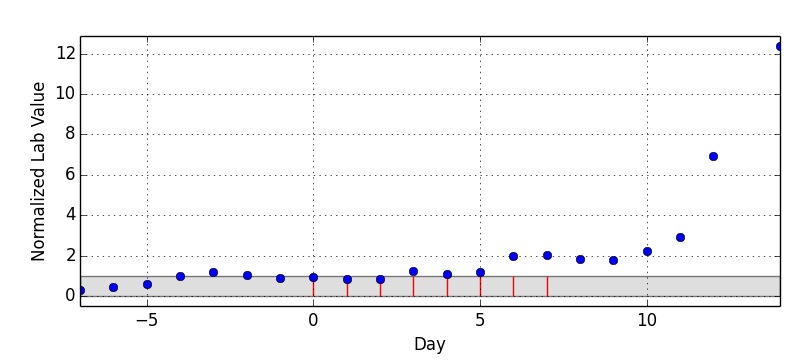

Supplement: S2 File — The “Curve Assessment Tool” (CAT) software application. This archive also contains the plots of all curves in Portable Network Graphics (PNG) format. (ZIP) [file pone.0136131.s002.zip › data/012.png]

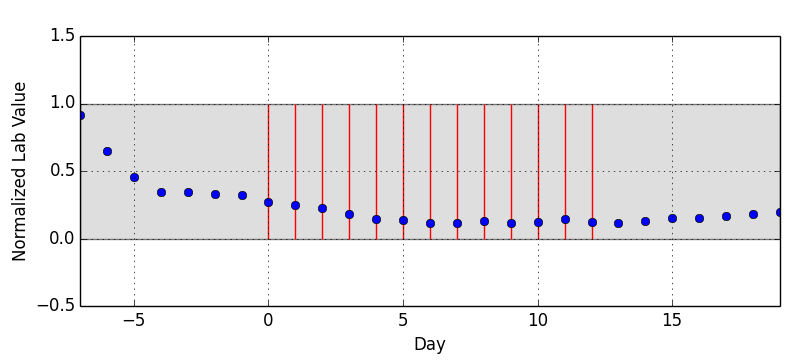

Supplement: S2 File — The “Curve Assessment Tool” (CAT) software application. This archive also contains the plots of all curves in Portable Network Graphics (PNG) format. (ZIP) [file pone.0136131.s002.zip › data/013.png]

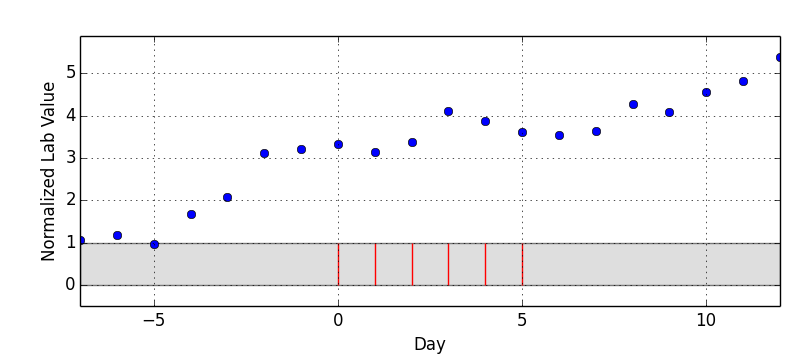

Supplement: S2 File — The “Curve Assessment Tool” (CAT) software application. This archive also contains the plots of all curves in Portable Network Graphics (PNG) format. (ZIP) [file pone.0136131.s002.zip › data/014.png]

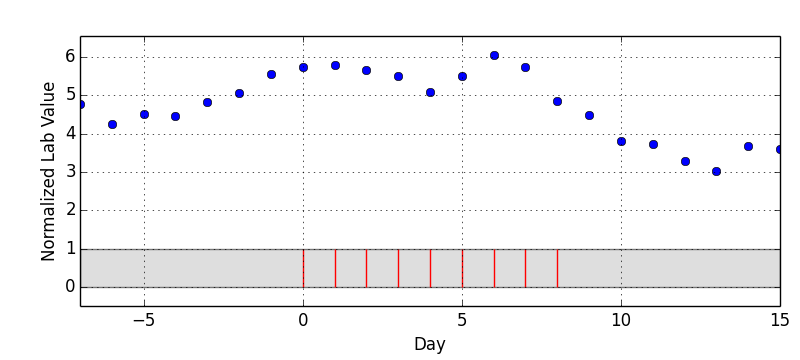

Supplement: S2 File — The “Curve Assessment Tool” (CAT) software application. This archive also contains the plots of all curves in Portable Network Graphics (PNG) format. (ZIP) [file pone.0136131.s002.zip › data/015.png]

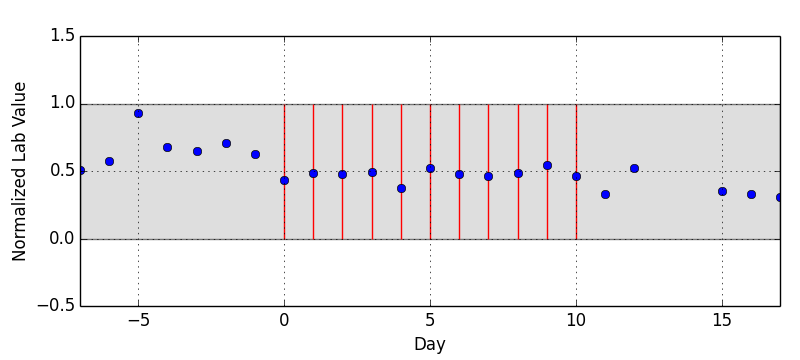

Supplement: S2 File — The “Curve Assessment Tool” (CAT) software application. This archive also contains the plots of all curves in Portable Network Graphics (PNG) format. (ZIP) [file pone.0136131.s002.zip › data/016.png]

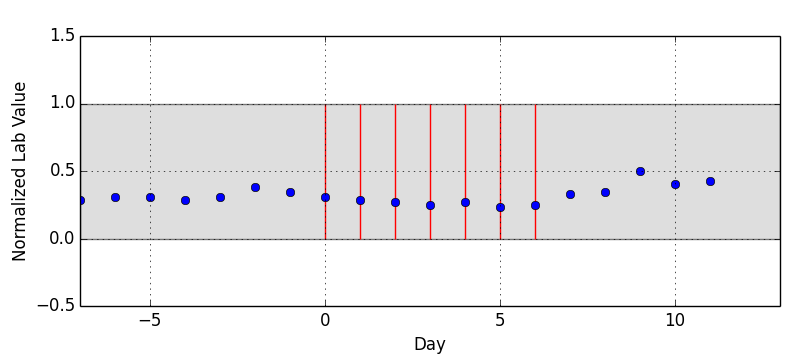

Supplement: S2 File — The “Curve Assessment Tool” (CAT) software application. This archive also contains the plots of all curves in Portable Network Graphics (PNG) format. (ZIP) [file pone.0136131.s002.zip › data/017.png]

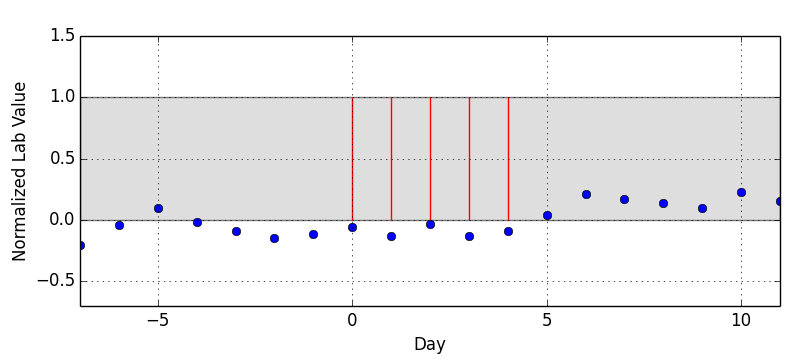

Supplement: S2 File — The “Curve Assessment Tool” (CAT) software application. This archive also contains the plots of all curves in Portable Network Graphics (PNG) format. (ZIP) [file pone.0136131.s002.zip › data/018.png]

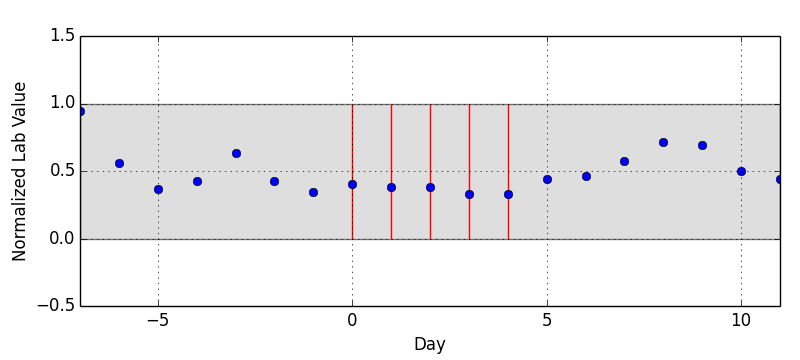

Supplement: S2 File — The “Curve Assessment Tool” (CAT) software application. This archive also contains the plots of all curves in Portable Network Graphics (PNG) format. (ZIP) [file pone.0136131.s002.zip › data/019.png]

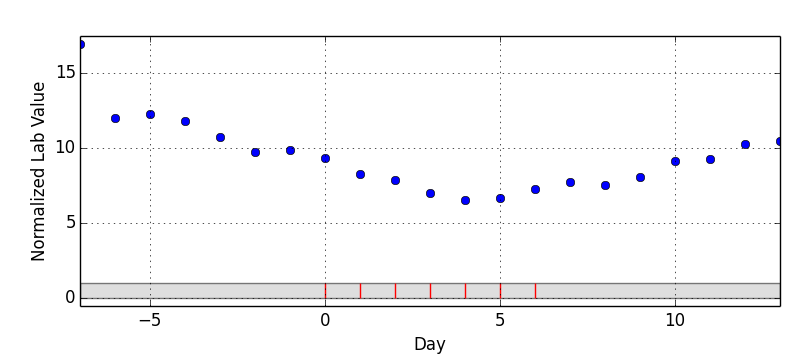

Supplement: S2 File — The “Curve Assessment Tool” (CAT) software application. This archive also contains the plots of all curves in Portable Network Graphics (PNG) format. (ZIP) [file pone.0136131.s002.zip › data/020.png]

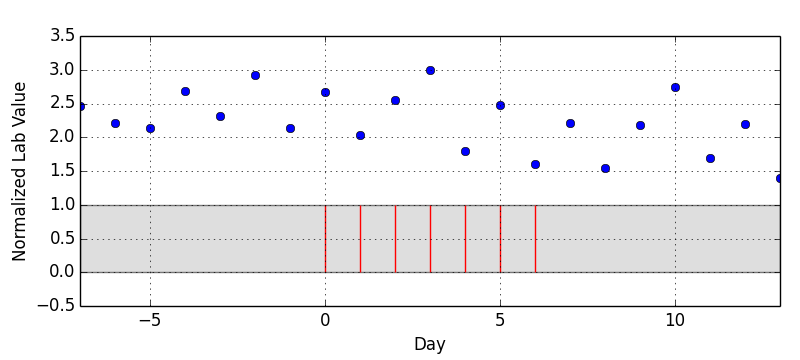

Supplement: S2 File — The “Curve Assessment Tool” (CAT) software application. This archive also contains the plots of all curves in Portable Network Graphics (PNG) format. (ZIP) [file pone.0136131.s002.zip › data/021.png]

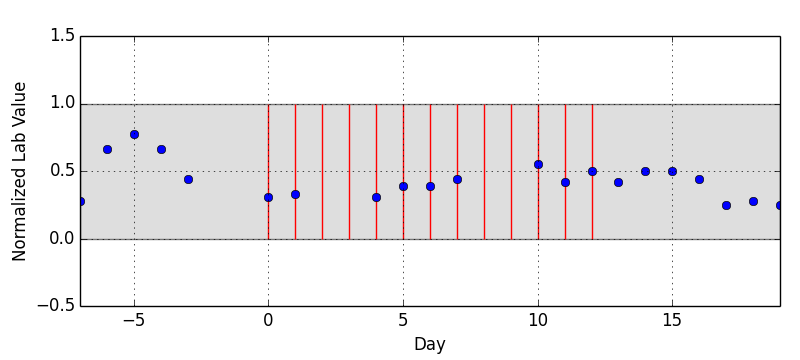

Supplement: S2 File — The “Curve Assessment Tool” (CAT) software application. This archive also contains the plots of all curves in Portable Network Graphics (PNG) format. (ZIP) [file pone.0136131.s002.zip › data/022.png]

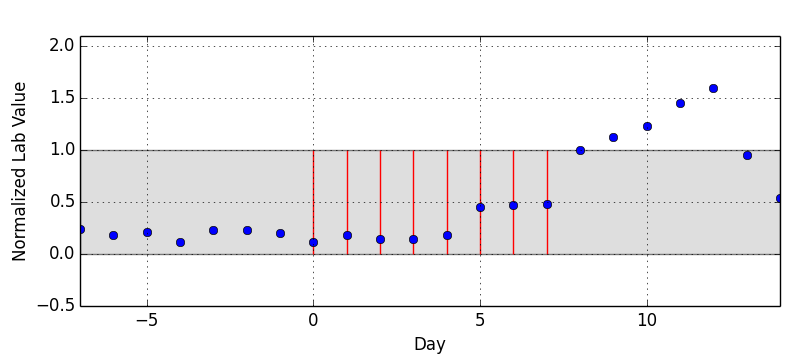

Supplement: S2 File — The “Curve Assessment Tool” (CAT) software application. This archive also contains the plots of all curves in Portable Network Graphics (PNG) format. (ZIP) [file pone.0136131.s002.zip › data/023.png]

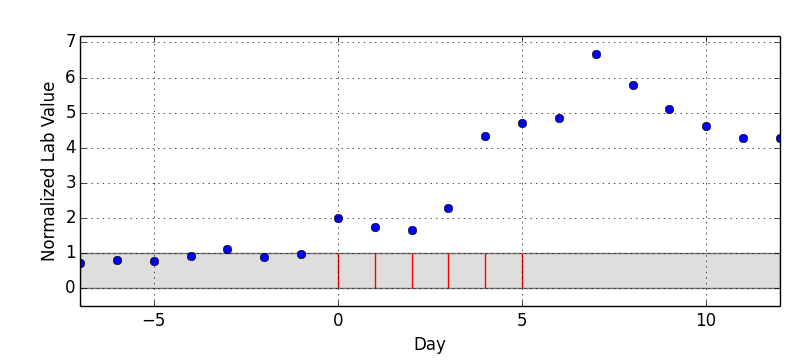

Supplement: S2 File — The “Curve Assessment Tool” (CAT) software application. This archive also contains the plots of all curves in Portable Network Graphics (PNG) format. (ZIP) [file pone.0136131.s002.zip › data/024.png]

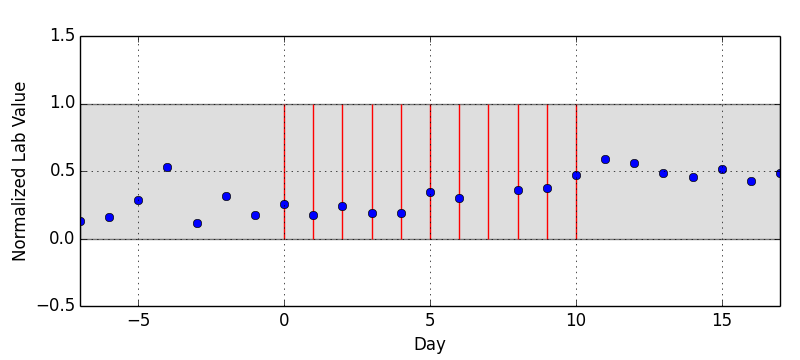

Supplement: S2 File — The “Curve Assessment Tool” (CAT) software application. This archive also contains the plots of all curves in Portable Network Graphics (PNG) format. (ZIP) [file pone.0136131.s002.zip › data/025.png]

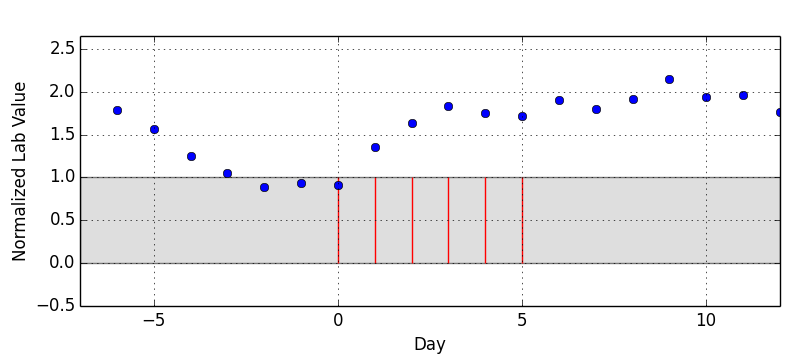

Supplement: S2 File — The “Curve Assessment Tool” (CAT) software application. This archive also contains the plots of all curves in Portable Network Graphics (PNG) format. (ZIP) [file pone.0136131.s002.zip › data/026.png]

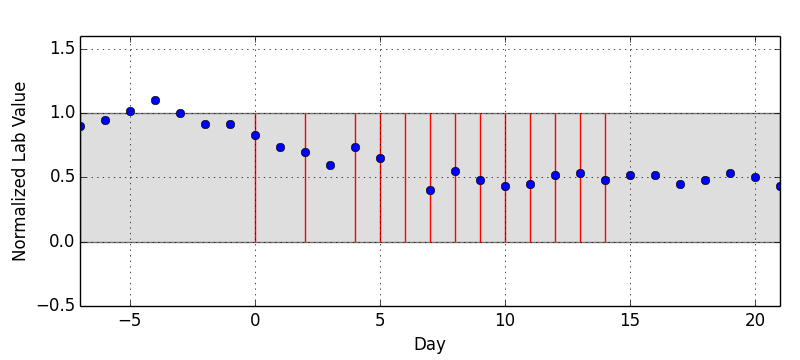

Supplement: S2 File — The “Curve Assessment Tool” (CAT) software application. This archive also contains the plots of all curves in Portable Network Graphics (PNG) format. (ZIP) [file pone.0136131.s002.zip › data/027.png]

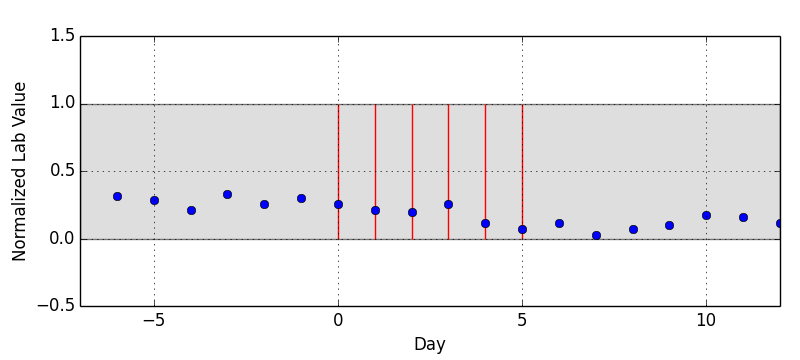

Supplement: S2 File — The “Curve Assessment Tool” (CAT) software application. This archive also contains the plots of all curves in Portable Network Graphics (PNG) format. (ZIP) [file pone.0136131.s002.zip › data/028.png]

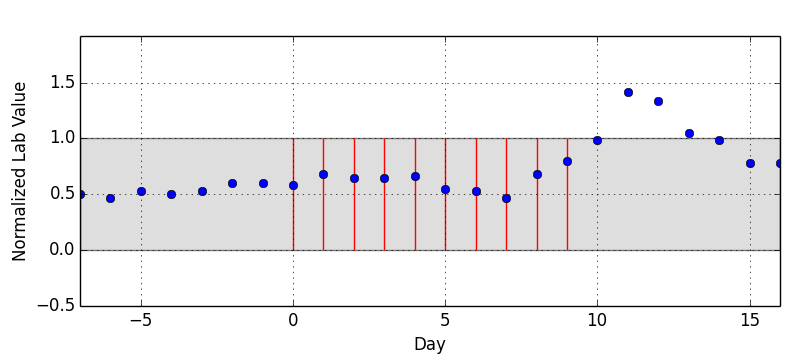

Supplement: S2 File — The “Curve Assessment Tool” (CAT) software application. This archive also contains the plots of all curves in Portable Network Graphics (PNG) format. (ZIP) [file pone.0136131.s002.zip › data/029.png]

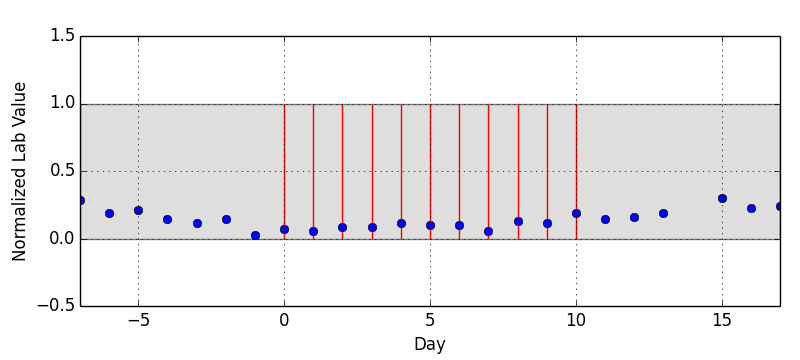

Supplement: S2 File — The “Curve Assessment Tool” (CAT) software application. This archive also contains the plots of all curves in Portable Network Graphics (PNG) format. (ZIP) [file pone.0136131.s002.zip › data/030.png]

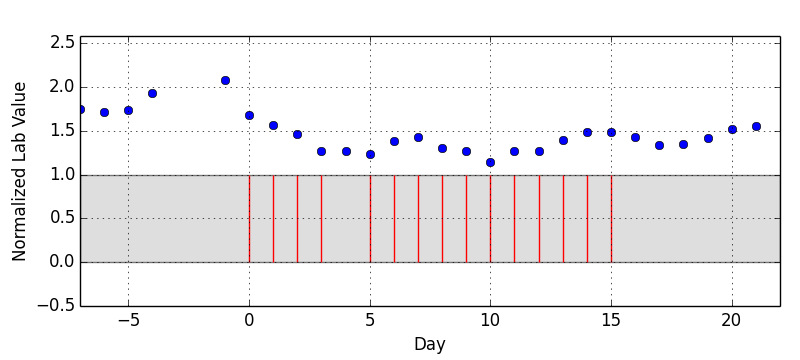

Supplement: S2 File — The “Curve Assessment Tool” (CAT) software application. This archive also contains the plots of all curves in Portable Network Graphics (PNG) format. (ZIP) [file pone.0136131.s002.zip › data/031.png]

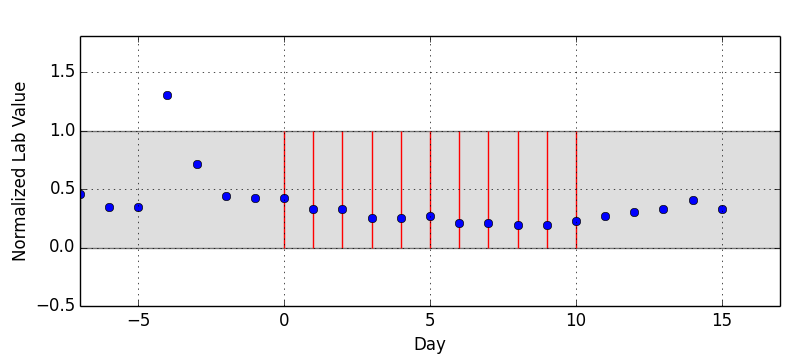

Supplement: S2 File — The “Curve Assessment Tool” (CAT) software application. This archive also contains the plots of all curves in Portable Network Graphics (PNG) format. (ZIP) [file pone.0136131.s002.zip › data/032.png]

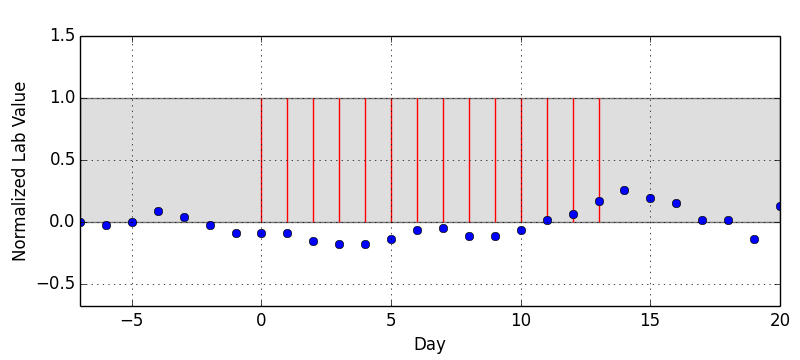

Supplement: S2 File — The “Curve Assessment Tool” (CAT) software application. This archive also contains the plots of all curves in Portable Network Graphics (PNG) format. (ZIP) [file pone.0136131.s002.zip › data/033.png]

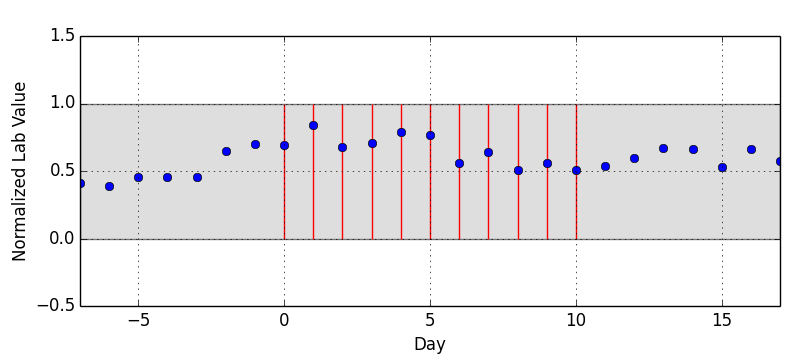

Supplement: S2 File — The “Curve Assessment Tool” (CAT) software application. This archive also contains the plots of all curves in Portable Network Graphics (PNG) format. (ZIP) [file pone.0136131.s002.zip › data/034.png]

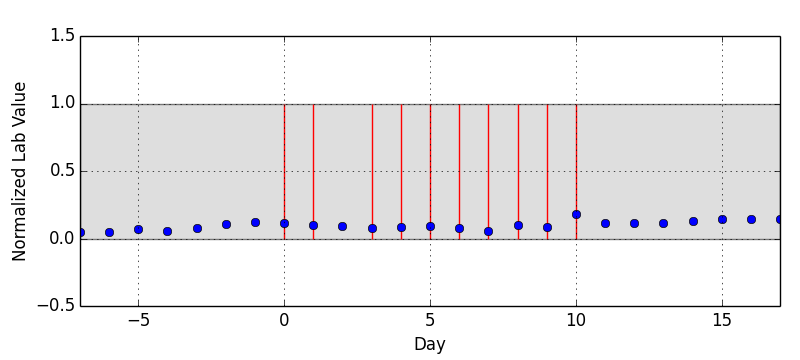

Supplement: S2 File — The “Curve Assessment Tool” (CAT) software application. This archive also contains the plots of all curves in Portable Network Graphics (PNG) format. (ZIP) [file pone.0136131.s002.zip › data/035.png]

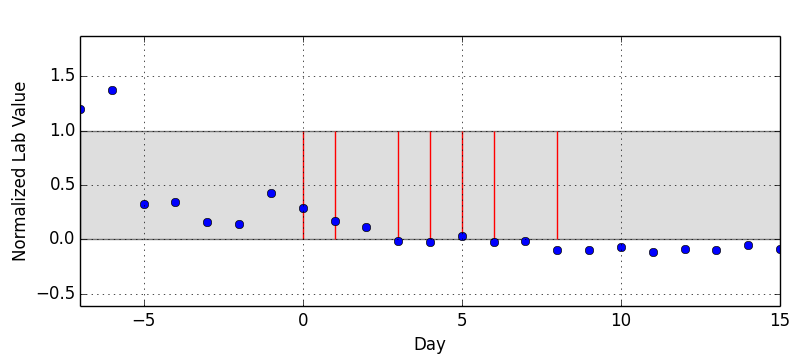

Supplement: S2 File — The “Curve Assessment Tool” (CAT) software application. This archive also contains the plots of all curves in Portable Network Graphics (PNG) format. (ZIP) [file pone.0136131.s002.zip › data/036.png]

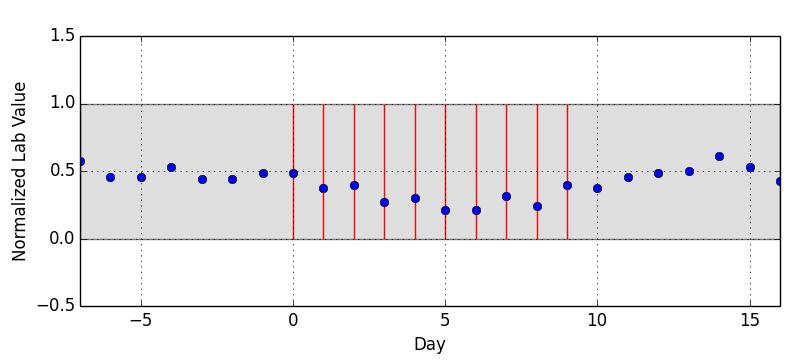

Supplement: S2 File — The “Curve Assessment Tool” (CAT) software application. This archive also contains the plots of all curves in Portable Network Graphics (PNG) format. (ZIP) [file pone.0136131.s002.zip › data/037.png]

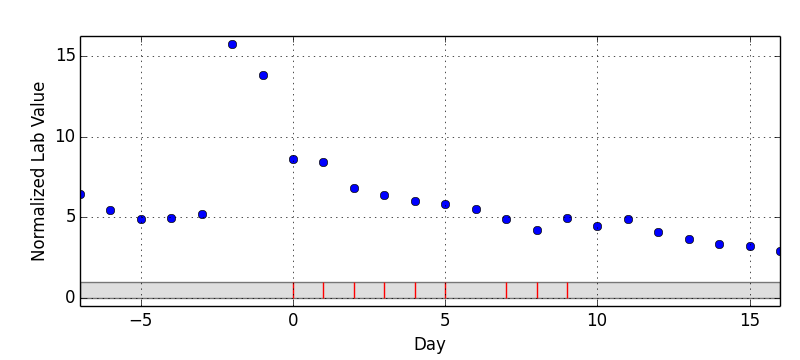

Supplement: S2 File — The “Curve Assessment Tool” (CAT) software application. This archive also contains the plots of all curves in Portable Network Graphics (PNG) format. (ZIP) [file pone.0136131.s002.zip › data/038.png]

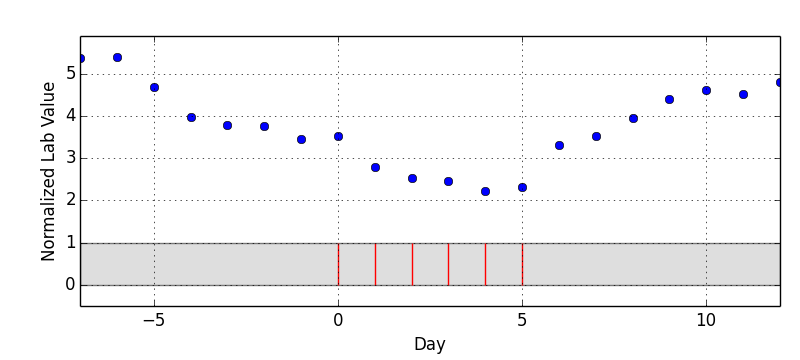

Supplement: S2 File — The “Curve Assessment Tool” (CAT) software application. This archive also contains the plots of all curves in Portable Network Graphics (PNG) format. (ZIP) [file pone.0136131.s002.zip › data/039.png]

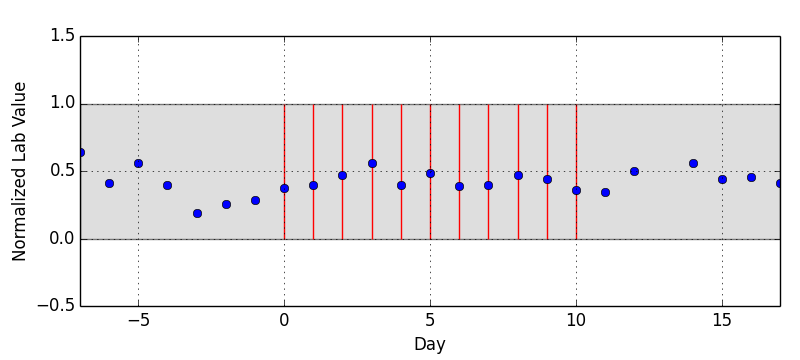

Supplement: S2 File — The “Curve Assessment Tool” (CAT) software application. This archive also contains the plots of all curves in Portable Network Graphics (PNG) format. (ZIP) [file pone.0136131.s002.zip › data/040.png]

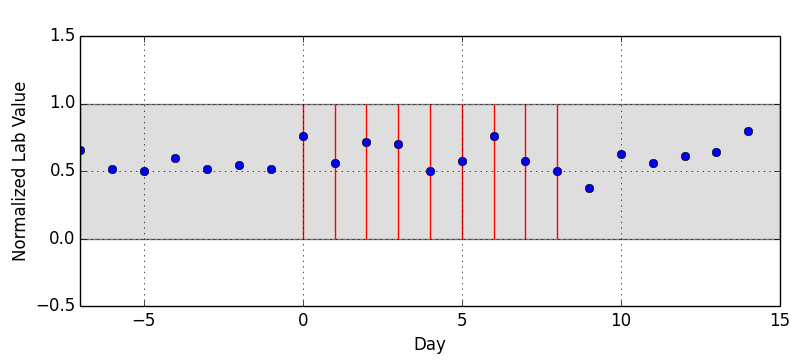

Supplement: S2 File — The “Curve Assessment Tool” (CAT) software application. This archive also contains the plots of all curves in Portable Network Graphics (PNG) format. (ZIP) [file pone.0136131.s002.zip › data/041.png]

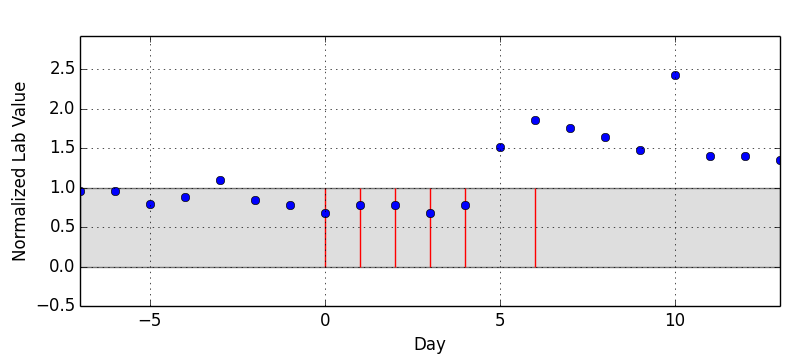

Supplement: S2 File — The “Curve Assessment Tool” (CAT) software application. This archive also contains the plots of all curves in Portable Network Graphics (PNG) format. (ZIP) [file pone.0136131.s002.zip › data/042.png]

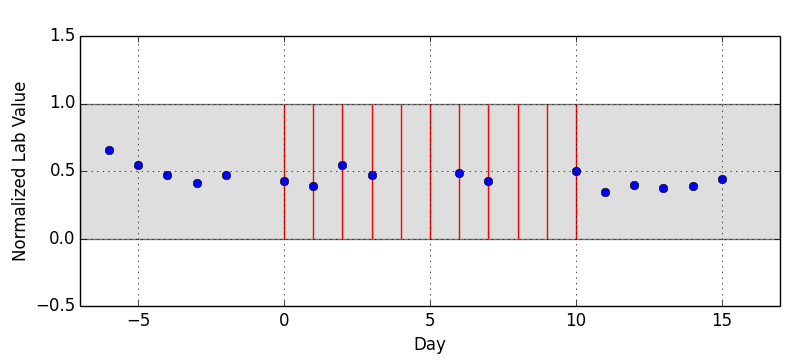

Supplement: S2 File — The “Curve Assessment Tool” (CAT) software application. This archive also contains the plots of all curves in Portable Network Graphics (PNG) format. (ZIP) [file pone.0136131.s002.zip › data/043.png]

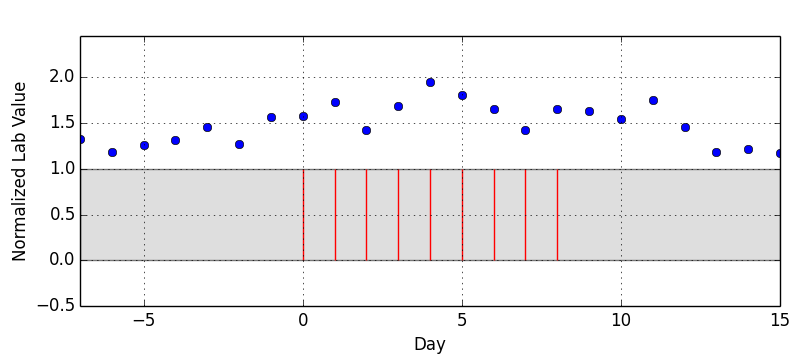

Supplement: S2 File — The “Curve Assessment Tool” (CAT) software application. This archive also contains the plots of all curves in Portable Network Graphics (PNG) format. (ZIP) [file pone.0136131.s002.zip › data/044.png]

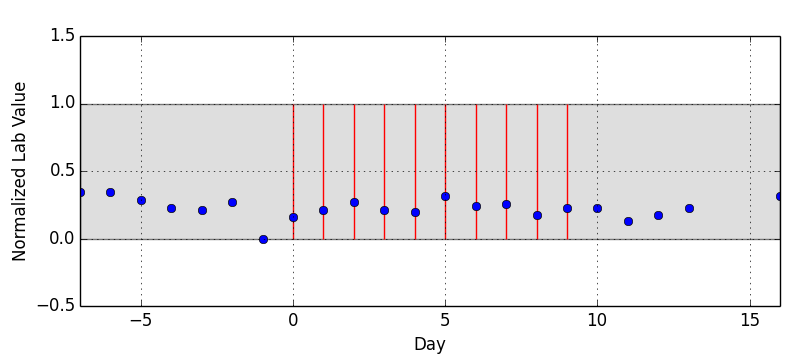

Supplement: S2 File — The “Curve Assessment Tool” (CAT) software application. This archive also contains the plots of all curves in Portable Network Graphics (PNG) format. (ZIP) [file pone.0136131.s002.zip › data/045.png]

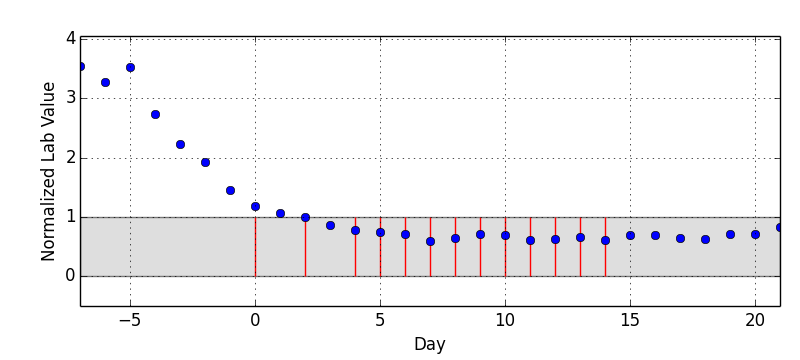

Supplement: S2 File — The “Curve Assessment Tool” (CAT) software application. This archive also contains the plots of all curves in Portable Network Graphics (PNG) format. (ZIP) [file pone.0136131.s002.zip › data/046.png]

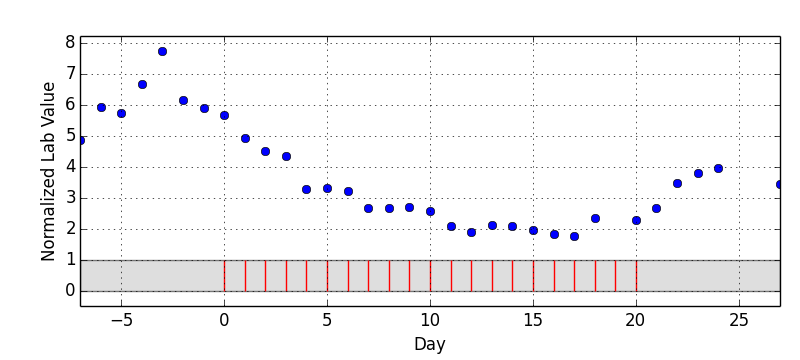

Supplement: S2 File — The “Curve Assessment Tool” (CAT) software application. This archive also contains the plots of all curves in Portable Network Graphics (PNG) format. (ZIP) [file pone.0136131.s002.zip › data/047.png]

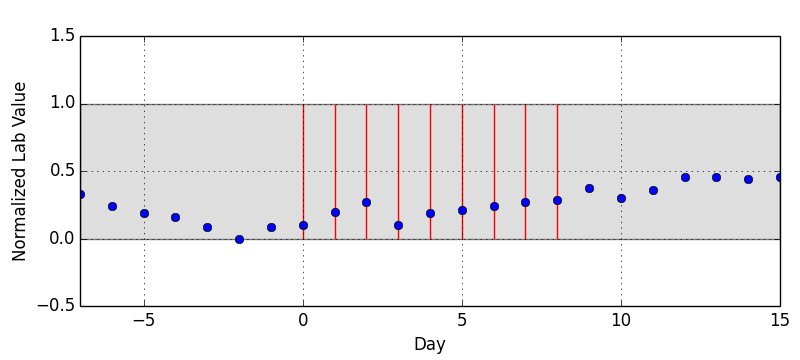

Supplement: S2 File — The “Curve Assessment Tool” (CAT) software application. This archive also contains the plots of all curves in Portable Network Graphics (PNG) format. (ZIP) [file pone.0136131.s002.zip › data/048.png]

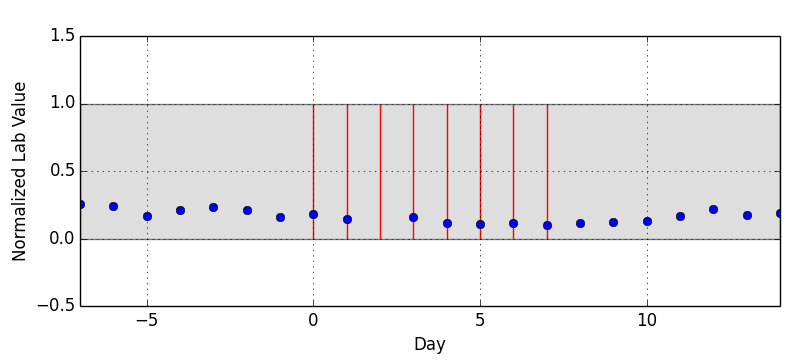

Supplement: S2 File — The “Curve Assessment Tool” (CAT) software application. This archive also contains the plots of all curves in Portable Network Graphics (PNG) format. (ZIP) [file pone.0136131.s002.zip › data/049.png]

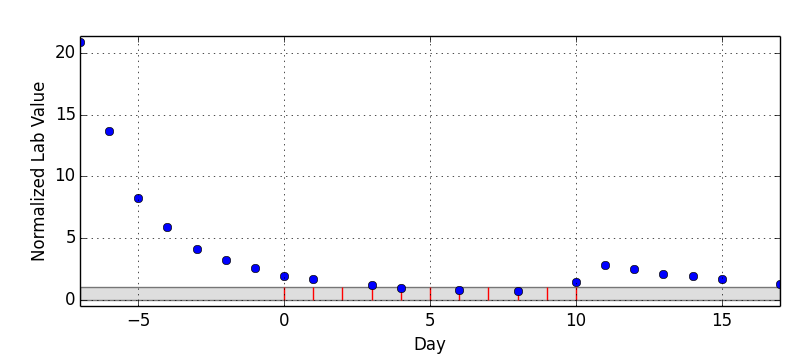

Supplement: S2 File — The “Curve Assessment Tool” (CAT) software application. This archive also contains the plots of all curves in Portable Network Graphics (PNG) format. (ZIP) [file pone.0136131.s002.zip › data/050.png]

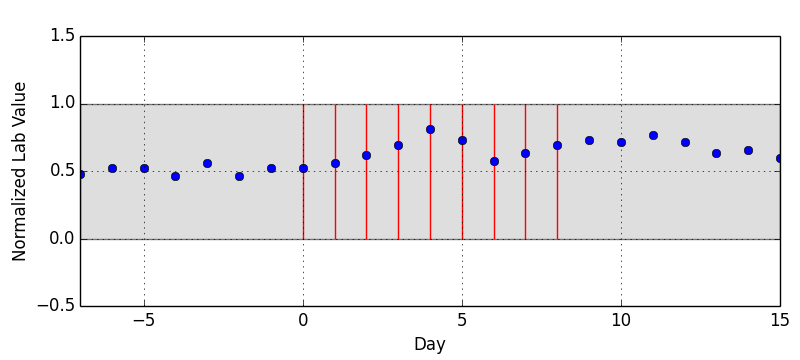

Supplement: S2 File — The “Curve Assessment Tool” (CAT) software application. This archive also contains the plots of all curves in Portable Network Graphics (PNG) format. (ZIP) [file pone.0136131.s002.zip › data/051.png]

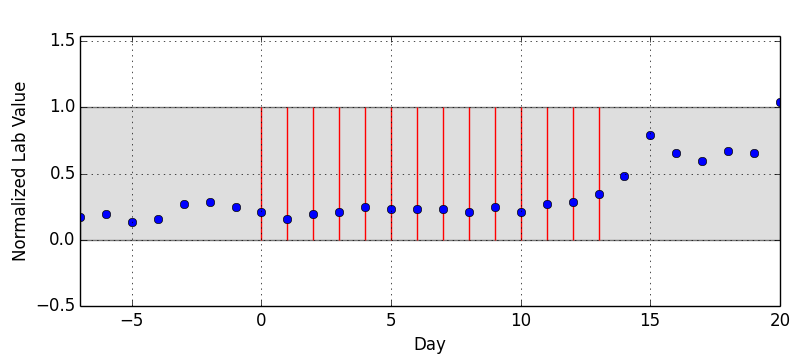

Supplement: S2 File — The “Curve Assessment Tool” (CAT) software application. This archive also contains the plots of all curves in Portable Network Graphics (PNG) format. (ZIP) [file pone.0136131.s002.zip › data/052.png]

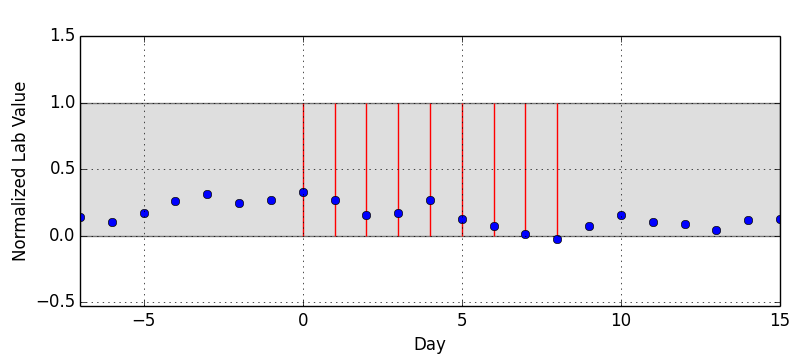

Supplement: S2 File — The “Curve Assessment Tool” (CAT) software application. This archive also contains the plots of all curves in Portable Network Graphics (PNG) format. (ZIP) [file pone.0136131.s002.zip › data/053.png]

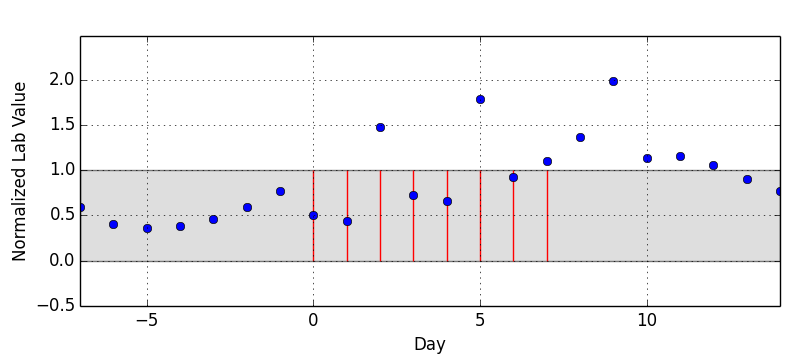

Supplement: S2 File — The “Curve Assessment Tool” (CAT) software application. This archive also contains the plots of all curves in Portable Network Graphics (PNG) format. (ZIP) [file pone.0136131.s002.zip › data/054.png]

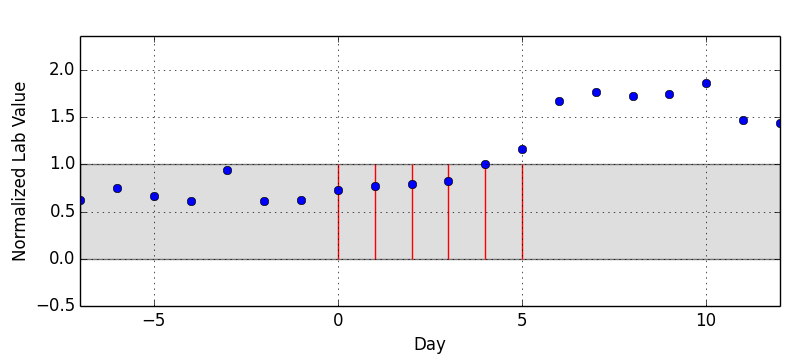

Supplement: S2 File — The “Curve Assessment Tool” (CAT) software application. This archive also contains the plots of all curves in Portable Network Graphics (PNG) format. (ZIP) [file pone.0136131.s002.zip › data/055.png]

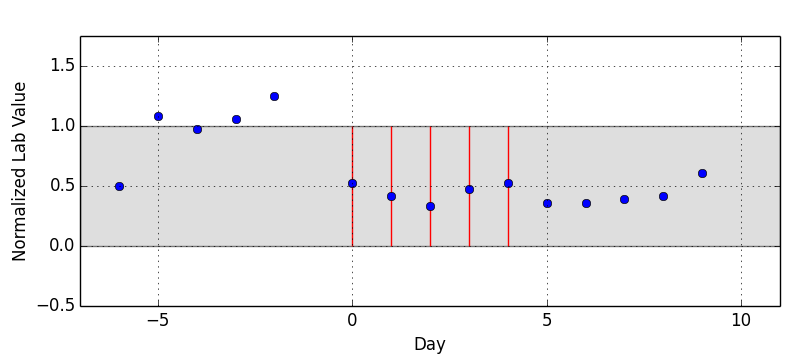

Supplement: S2 File — The “Curve Assessment Tool” (CAT) software application. This archive also contains the plots of all curves in Portable Network Graphics (PNG) format. (ZIP) [file pone.0136131.s002.zip › data/056.png]

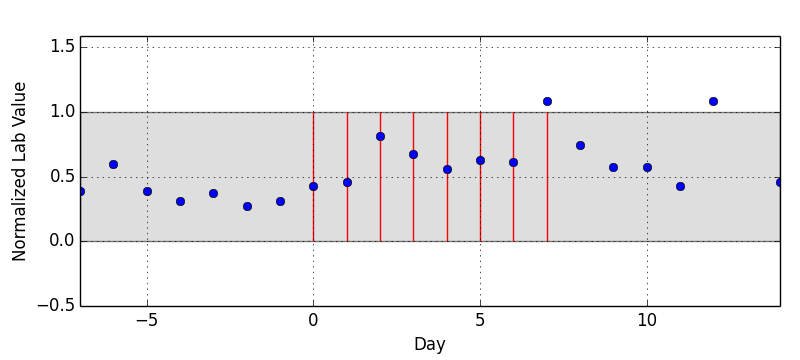

Supplement: S2 File — The “Curve Assessment Tool” (CAT) software application. This archive also contains the plots of all curves in Portable Network Graphics (PNG) format. (ZIP) [file pone.0136131.s002.zip › data/057.png]

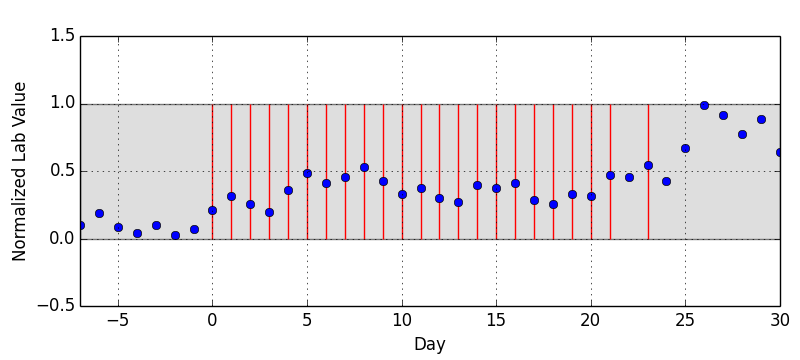

Supplement: S2 File — The “Curve Assessment Tool” (CAT) software application. This archive also contains the plots of all curves in Portable Network Graphics (PNG) format. (ZIP) [file pone.0136131.s002.zip › data/058.png]

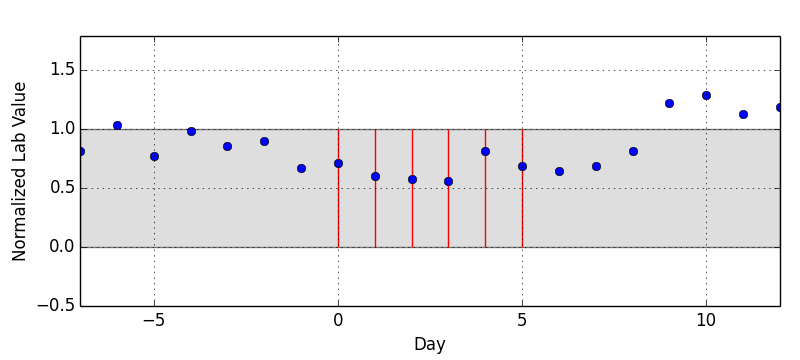

Supplement: S2 File — The “Curve Assessment Tool” (CAT) software application. This archive also contains the plots of all curves in Portable Network Graphics (PNG) format. (ZIP) [file pone.0136131.s002.zip › data/059.png]

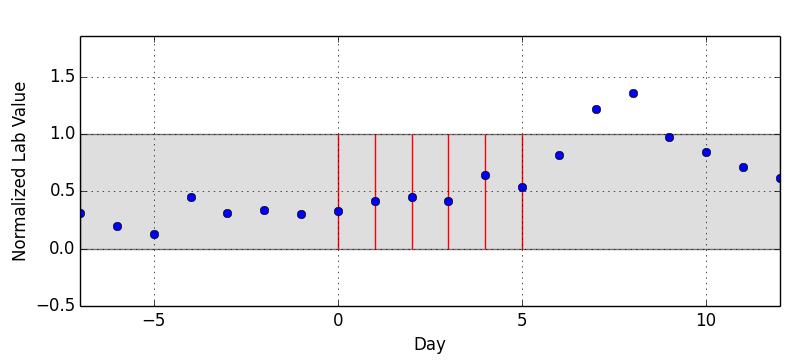

Supplement: S2 File — The “Curve Assessment Tool” (CAT) software application. This archive also contains the plots of all curves in Portable Network Graphics (PNG) format. (ZIP) [file pone.0136131.s002.zip › data/060.png]

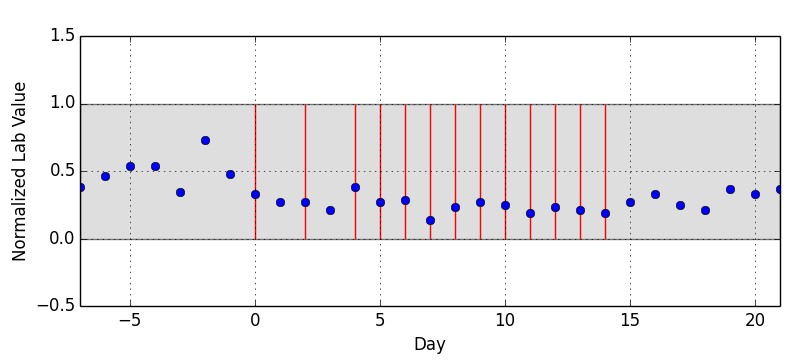

Supplement: S2 File — The “Curve Assessment Tool” (CAT) software application. This archive also contains the plots of all curves in Portable Network Graphics (PNG) format. (ZIP) [file pone.0136131.s002.zip › data/061.png]

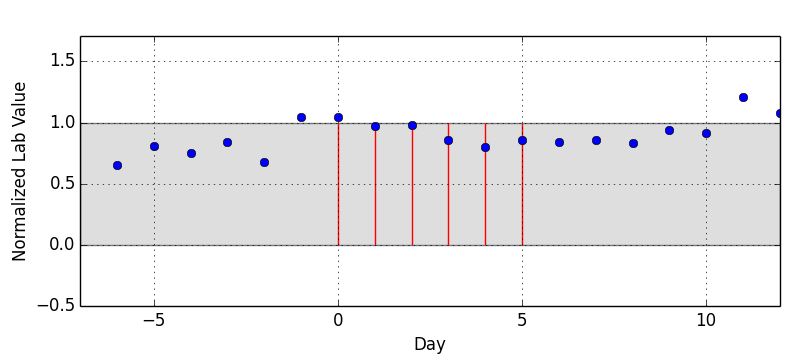

Supplement: S2 File — The “Curve Assessment Tool” (CAT) software application. This archive also contains the plots of all curves in Portable Network Graphics (PNG) format. (ZIP) [file pone.0136131.s002.zip › data/062.png]

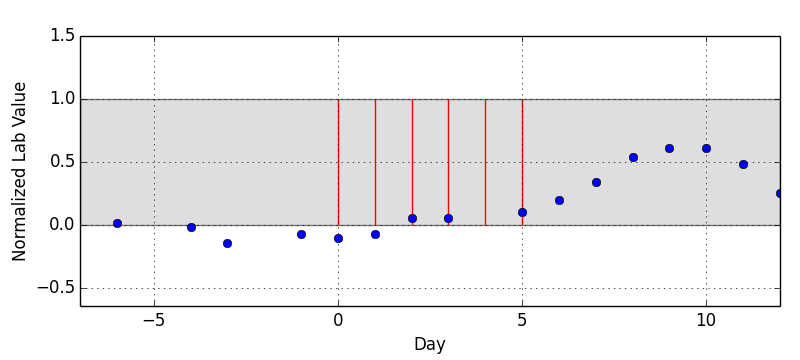

Supplement: S2 File — The “Curve Assessment Tool” (CAT) software application. This archive also contains the plots of all curves in Portable Network Graphics (PNG) format. (ZIP) [file pone.0136131.s002.zip › data/063.png]

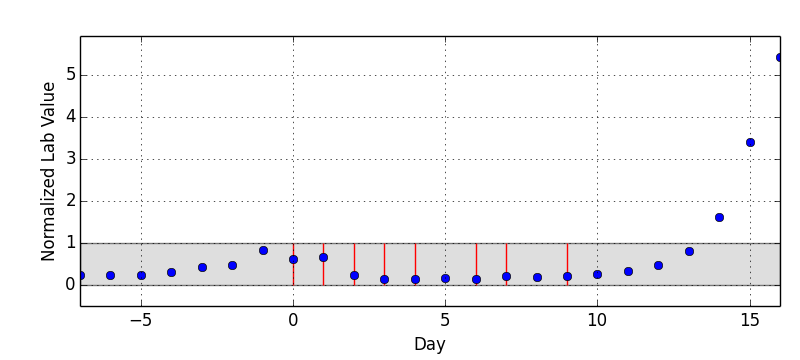

Supplement: S2 File — The “Curve Assessment Tool” (CAT) software application. This archive also contains the plots of all curves in Portable Network Graphics (PNG) format. (ZIP) [file pone.0136131.s002.zip › data/064.png]

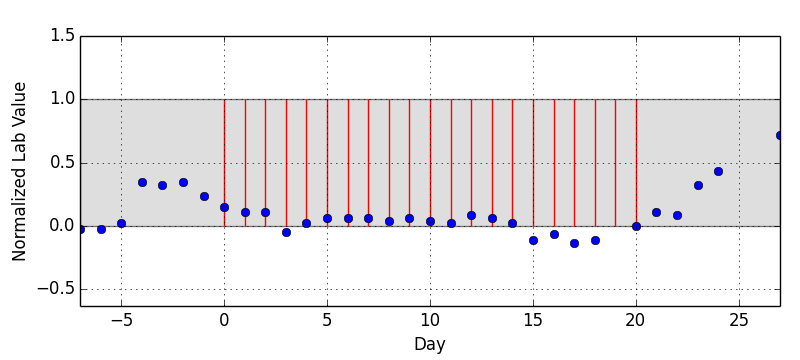

Supplement: S2 File — The “Curve Assessment Tool” (CAT) software application. This archive also contains the plots of all curves in Portable Network Graphics (PNG) format. (ZIP) [file pone.0136131.s002.zip › data/065.png]

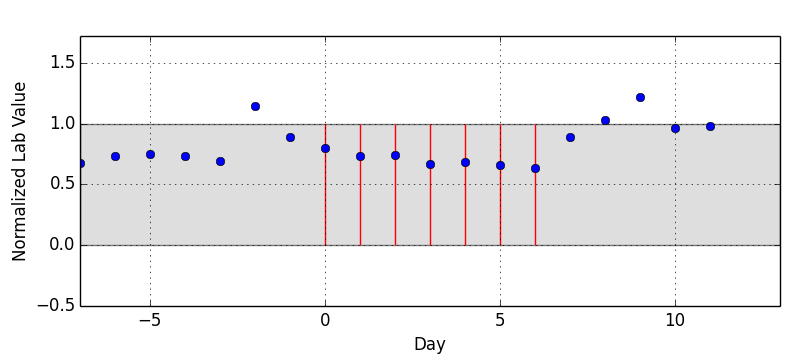

Supplement: S2 File — The “Curve Assessment Tool” (CAT) software application. This archive also contains the plots of all curves in Portable Network Graphics (PNG) format. (ZIP) [file pone.0136131.s002.zip › data/066.png]

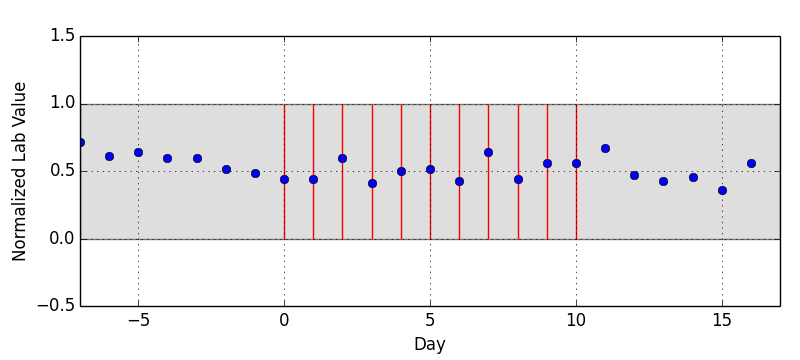

Supplement: S2 File — The “Curve Assessment Tool” (CAT) software application. This archive also contains the plots of all curves in Portable Network Graphics (PNG) format. (ZIP) [file pone.0136131.s002.zip › data/067.png]

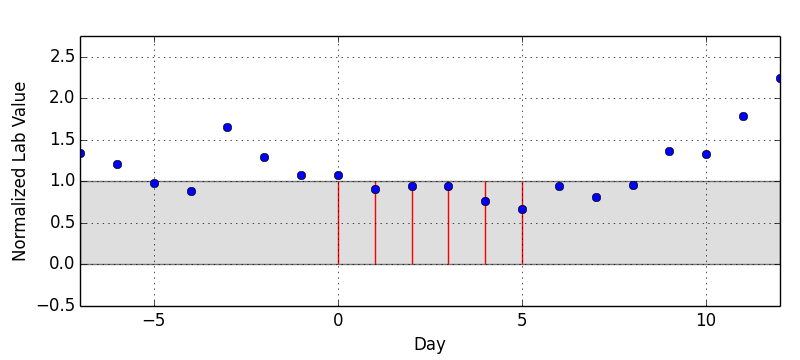

Supplement: S2 File — The “Curve Assessment Tool” (CAT) software application. This archive also contains the plots of all curves in Portable Network Graphics (PNG) format. (ZIP) [file pone.0136131.s002.zip › data/068.png]

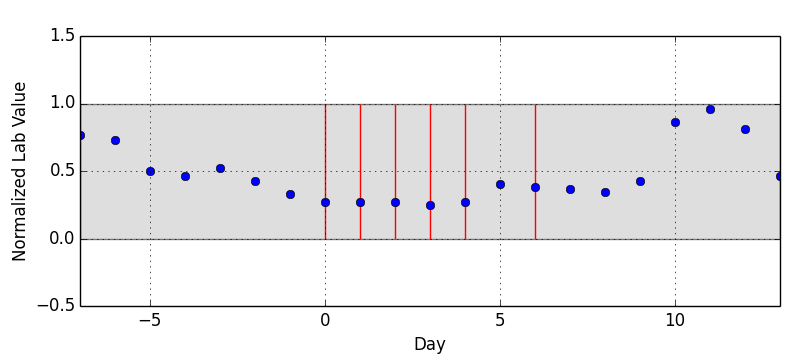

Supplement: S2 File — The “Curve Assessment Tool” (CAT) software application. This archive also contains the plots of all curves in Portable Network Graphics (PNG) format. (ZIP) [file pone.0136131.s002.zip › data/069.png]

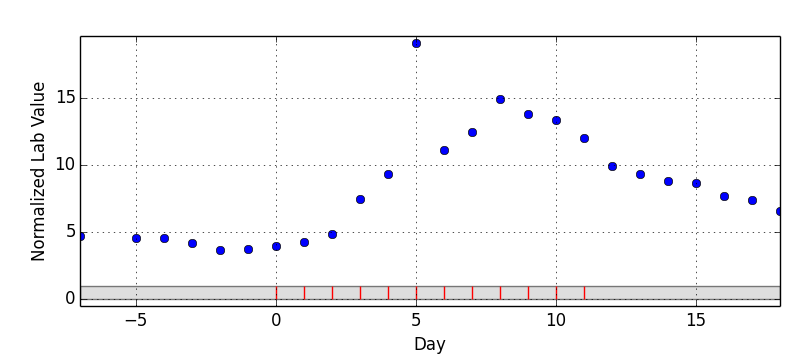

Supplement: S2 File — The “Curve Assessment Tool” (CAT) software application. This archive also contains the plots of all curves in Portable Network Graphics (PNG) format. (ZIP) [file pone.0136131.s002.zip › data/070.png]

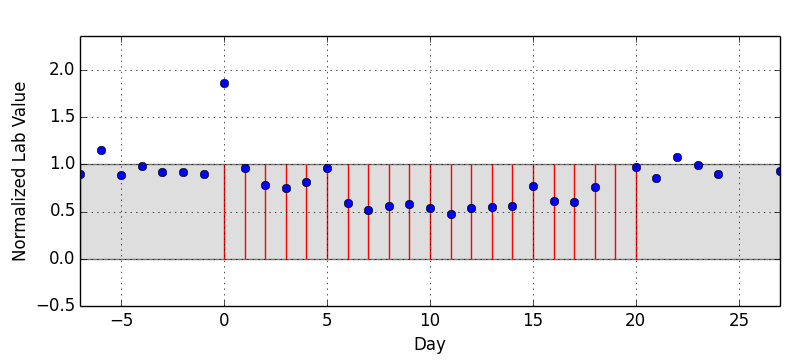

Supplement: S2 File — The “Curve Assessment Tool” (CAT) software application. This archive also contains the plots of all curves in Portable Network Graphics (PNG) format. (ZIP) [file pone.0136131.s002.zip › data/071.png]

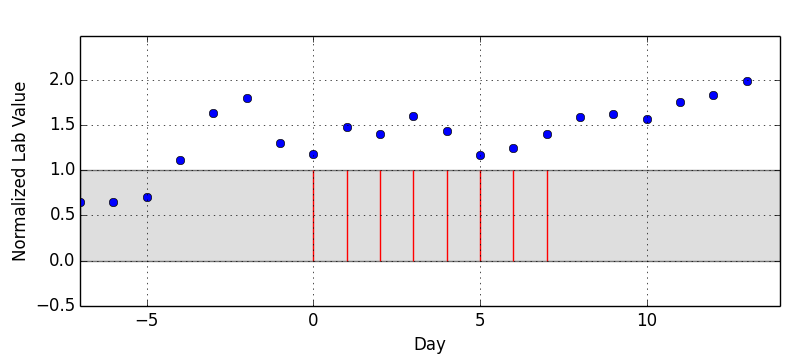

Supplement: S2 File — The “Curve Assessment Tool” (CAT) software application. This archive also contains the plots of all curves in Portable Network Graphics (PNG) format. (ZIP) [file pone.0136131.s002.zip › data/072.png]

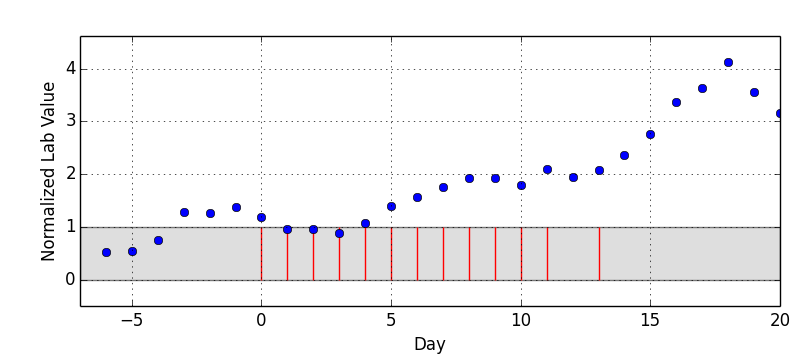

Supplement: S2 File — The “Curve Assessment Tool” (CAT) software application. This archive also contains the plots of all curves in Portable Network Graphics (PNG) format. (ZIP) [file pone.0136131.s002.zip › data/073.png]

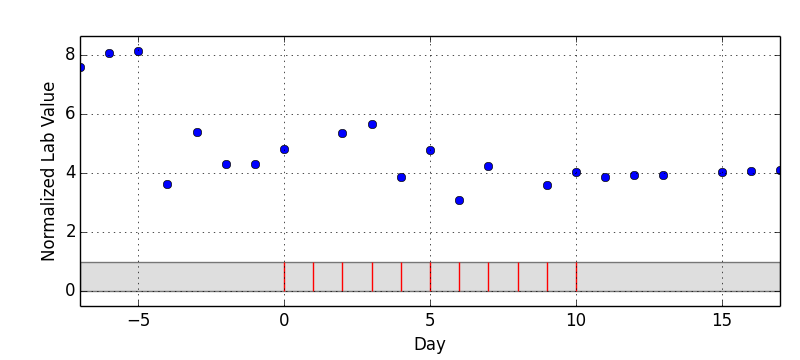

Supplement: S2 File — The “Curve Assessment Tool” (CAT) software application. This archive also contains the plots of all curves in Portable Network Graphics (PNG) format. (ZIP) [file pone.0136131.s002.zip › data/074.png]

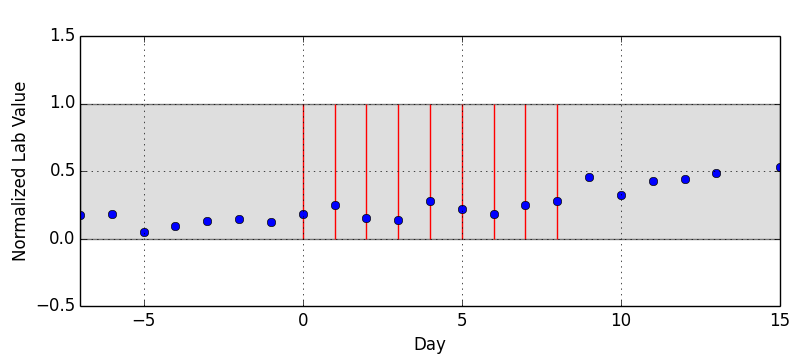

Supplement: S2 File — The “Curve Assessment Tool” (CAT) software application. This archive also contains the plots of all curves in Portable Network Graphics (PNG) format. (ZIP) [file pone.0136131.s002.zip › data/075.png]

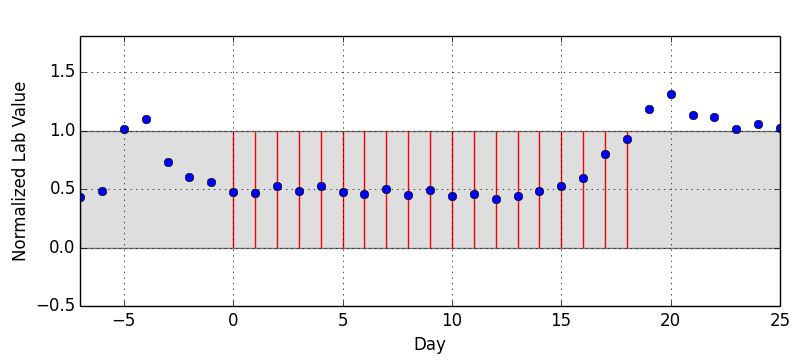

Supplement: S2 File — The “Curve Assessment Tool” (CAT) software application. This archive also contains the plots of all curves in Portable Network Graphics (PNG) format. (ZIP) [file pone.0136131.s002.zip › data/076.png]

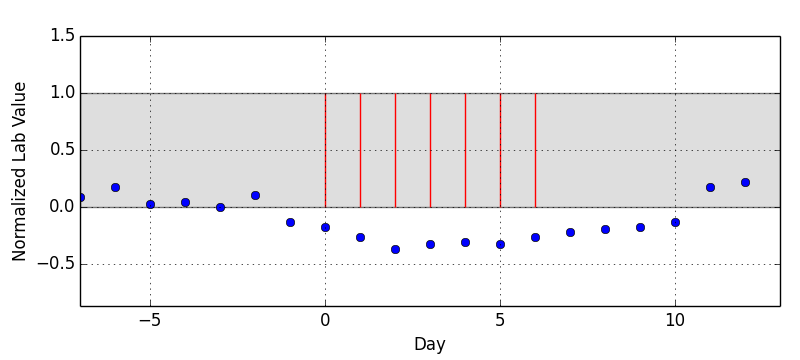

Supplement: S2 File — The “Curve Assessment Tool” (CAT) software application. This archive also contains the plots of all curves in Portable Network Graphics (PNG) format. (ZIP) [file pone.0136131.s002.zip › data/077.png]

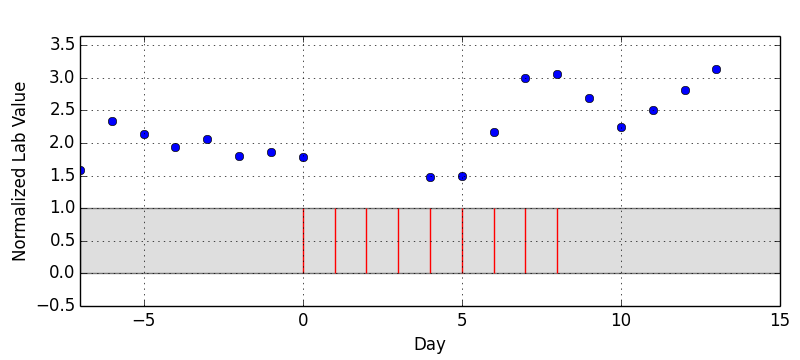

Supplement: S2 File — The “Curve Assessment Tool” (CAT) software application. This archive also contains the plots of all curves in Portable Network Graphics (PNG) format. (ZIP) [file pone.0136131.s002.zip › data/078.png]

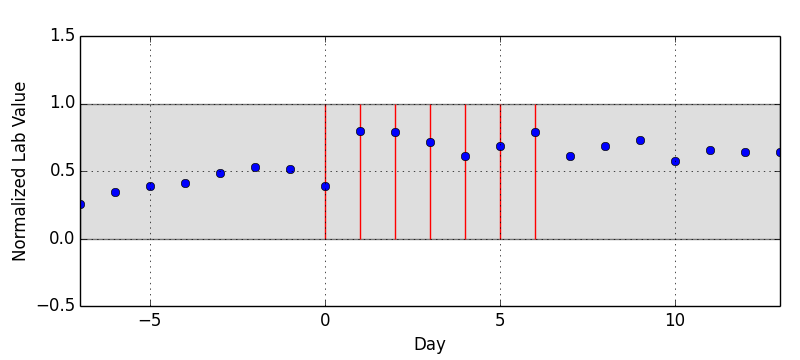

Supplement: S2 File — The “Curve Assessment Tool” (CAT) software application. This archive also contains the plots of all curves in Portable Network Graphics (PNG) format. (ZIP) [file pone.0136131.s002.zip › data/079.png]

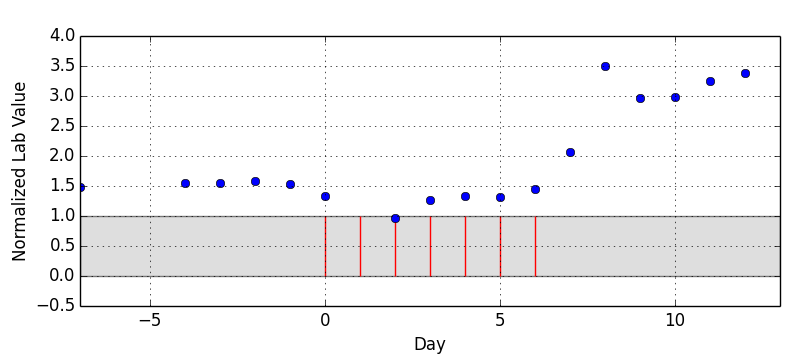

Supplement: S2 File — The “Curve Assessment Tool” (CAT) software application. This archive also contains the plots of all curves in Portable Network Graphics (PNG) format. (ZIP) [file pone.0136131.s002.zip › data/080.png]

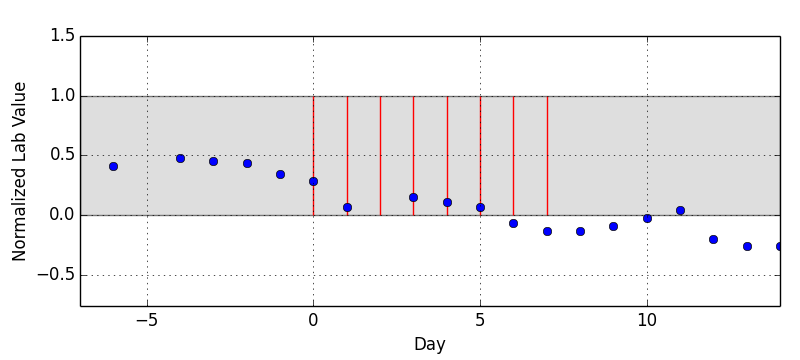

Supplement: S2 File — The “Curve Assessment Tool” (CAT) software application. This archive also contains the plots of all curves in Portable Network Graphics (PNG) format. (ZIP) [file pone.0136131.s002.zip › data/081.png]

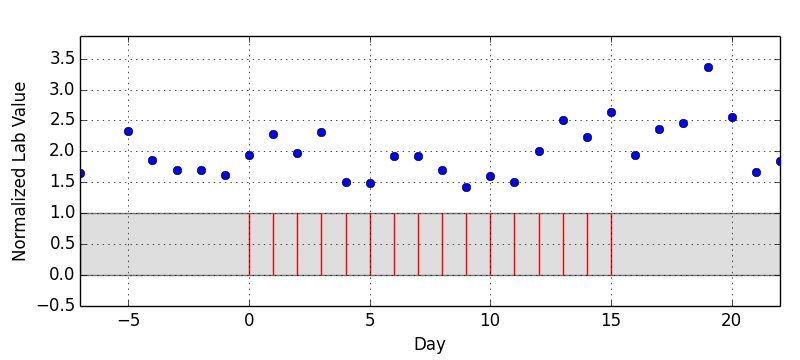

Supplement: S2 File — The “Curve Assessment Tool” (CAT) software application. This archive also contains the plots of all curves in Portable Network Graphics (PNG) format. (ZIP) [file pone.0136131.s002.zip › data/082.png]

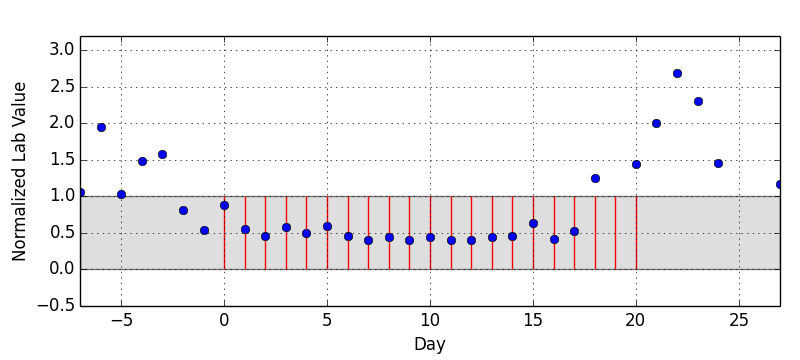

Supplement: S2 File — The “Curve Assessment Tool” (CAT) software application. This archive also contains the plots of all curves in Portable Network Graphics (PNG) format. (ZIP) [file pone.0136131.s002.zip › data/083.png]

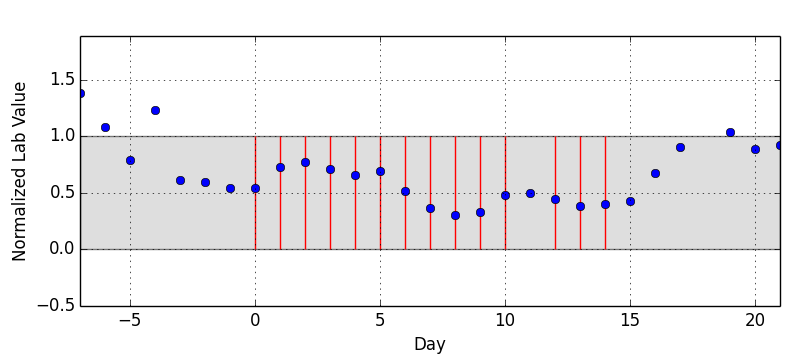

Supplement: S2 File — The “Curve Assessment Tool” (CAT) software application. This archive also contains the plots of all curves in Portable Network Graphics (PNG) format. (ZIP) [file pone.0136131.s002.zip › data/084.png]

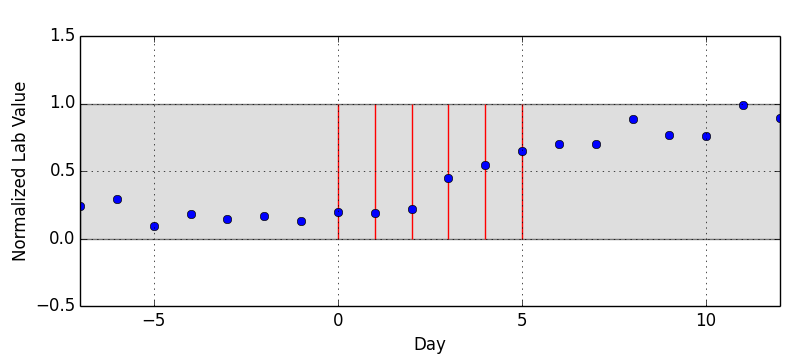

Supplement: S2 File — The “Curve Assessment Tool” (CAT) software application. This archive also contains the plots of all curves in Portable Network Graphics (PNG) format. (ZIP) [file pone.0136131.s002.zip › data/085.png]

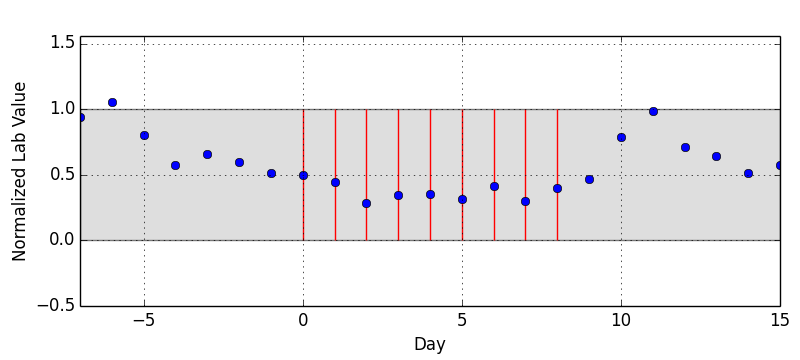

Supplement: S2 File — The “Curve Assessment Tool” (CAT) software application. This archive also contains the plots of all curves in Portable Network Graphics (PNG) format. (ZIP) [file pone.0136131.s002.zip › data/086.png]

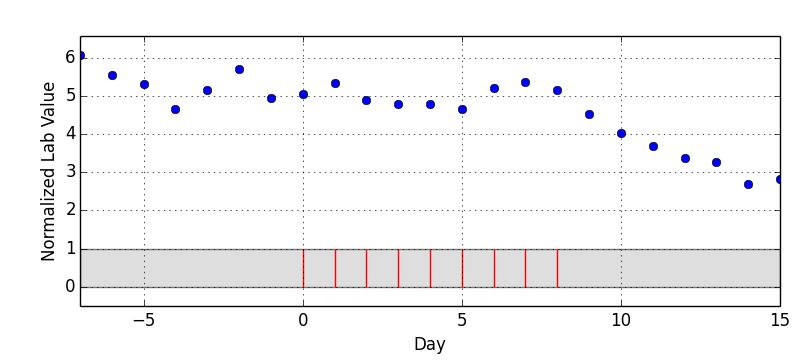

Supplement: S2 File — The “Curve Assessment Tool” (CAT) software application. This archive also contains the plots of all curves in Portable Network Graphics (PNG) format. (ZIP) [file pone.0136131.s002.zip › data/087.png]

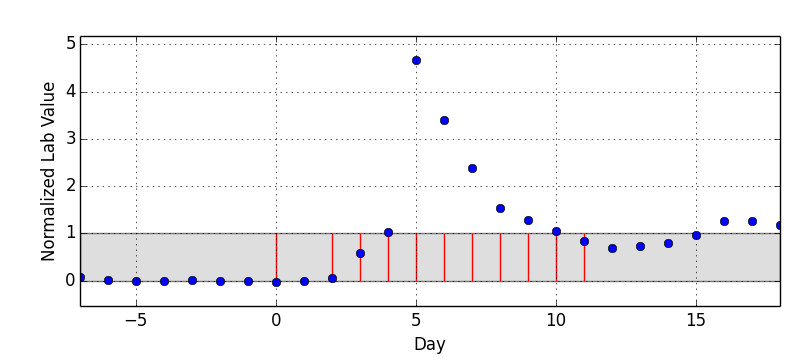

Supplement: S2 File — The “Curve Assessment Tool” (CAT) software application. This archive also contains the plots of all curves in Portable Network Graphics (PNG) format. (ZIP) [file pone.0136131.s002.zip › data/088.png]

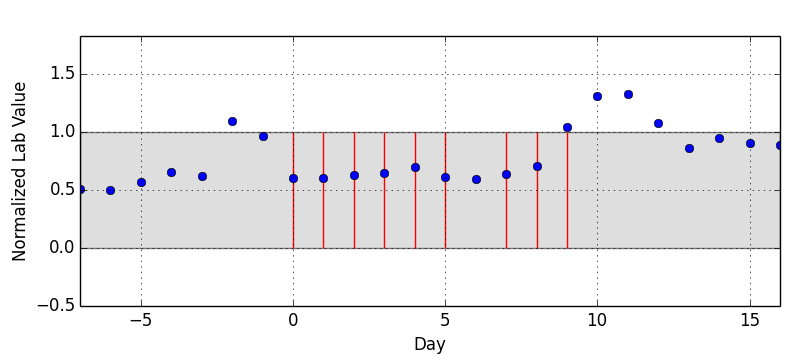

Supplement: S2 File — The “Curve Assessment Tool” (CAT) software application. This archive also contains the plots of all curves in Portable Network Graphics (PNG) format. (ZIP) [file pone.0136131.s002.zip › data/089.png]

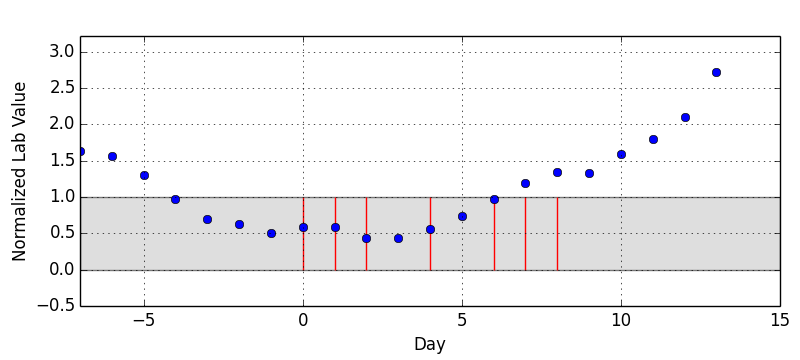

Supplement: S2 File — The “Curve Assessment Tool” (CAT) software application. This archive also contains the plots of all curves in Portable Network Graphics (PNG) format. (ZIP) [file pone.0136131.s002.zip › data/090.png]

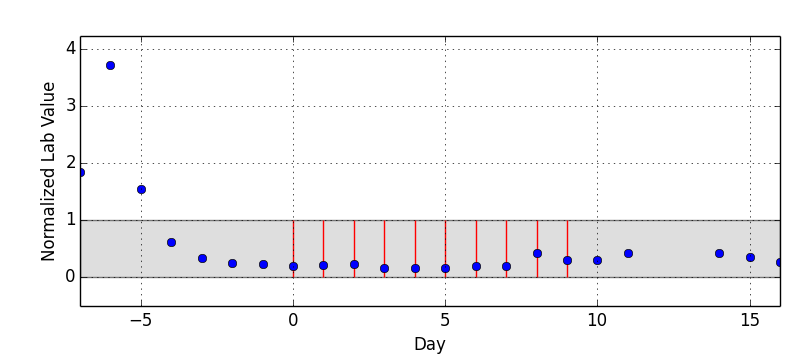

Supplement: S2 File — The “Curve Assessment Tool” (CAT) software application. This archive also contains the plots of all curves in Portable Network Graphics (PNG) format. (ZIP) [file pone.0136131.s002.zip › data/091.png]

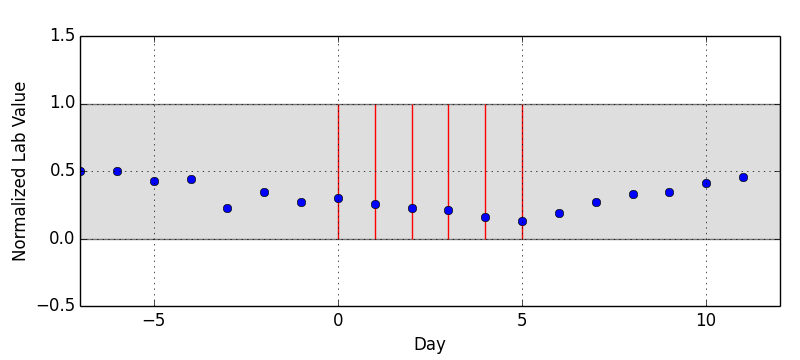

Supplement: S2 File — The “Curve Assessment Tool” (CAT) software application. This archive also contains the plots of all curves in Portable Network Graphics (PNG) format. (ZIP) [file pone.0136131.s002.zip › data/092.png]

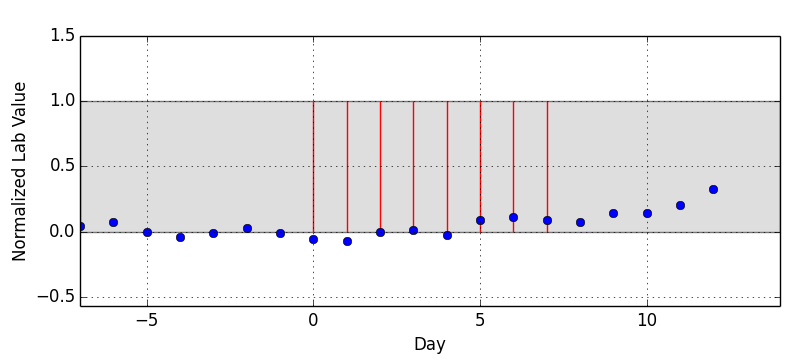

Supplement: S2 File — The “Curve Assessment Tool” (CAT) software application. This archive also contains the plots of all curves in Portable Network Graphics (PNG) format. (ZIP) [file pone.0136131.s002.zip › data/093.png]

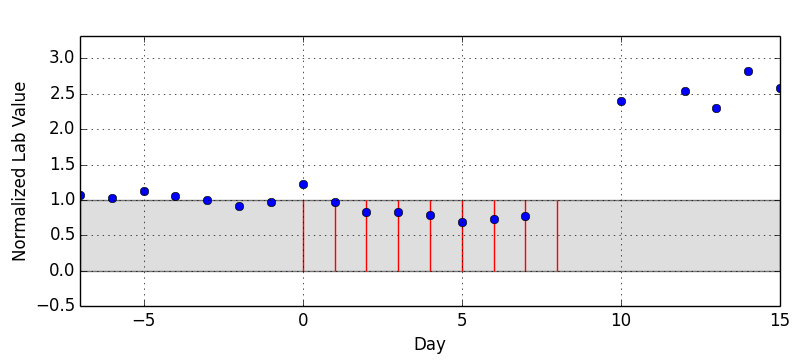

Supplement: S2 File — The “Curve Assessment Tool” (CAT) software application. This archive also contains the plots of all curves in Portable Network Graphics (PNG) format. (ZIP) [file pone.0136131.s002.zip › data/094.png]

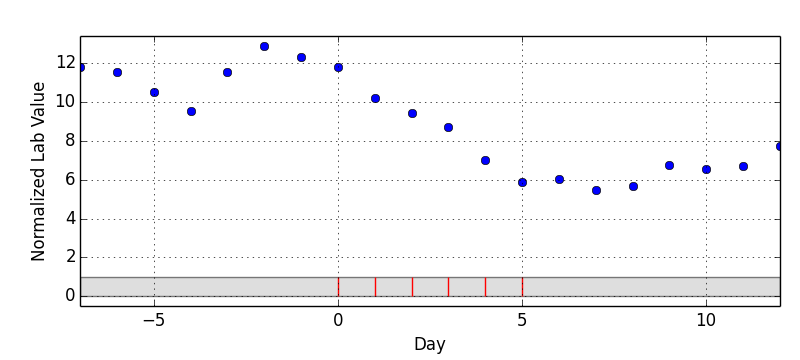

Supplement: S2 File — The “Curve Assessment Tool” (CAT) software application. This archive also contains the plots of all curves in Portable Network Graphics (PNG) format. (ZIP) [file pone.0136131.s002.zip › data/095.png]

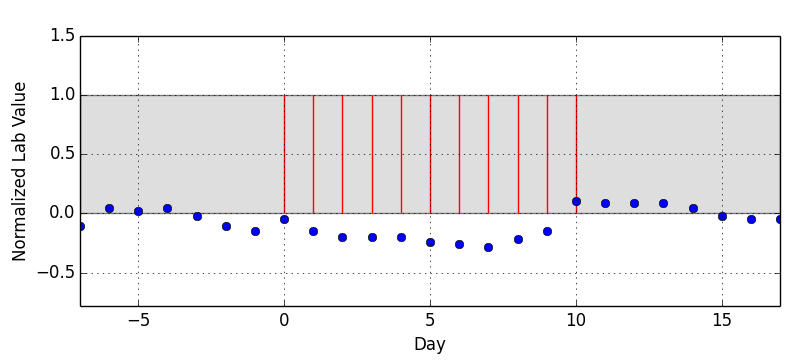

Supplement: S2 File — The “Curve Assessment Tool” (CAT) software application. This archive also contains the plots of all curves in Portable Network Graphics (PNG) format. (ZIP) [file pone.0136131.s002.zip › data/096.png]

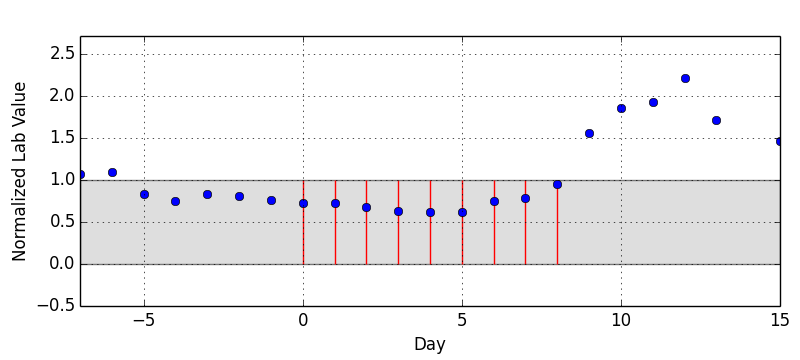

Supplement: S2 File — The “Curve Assessment Tool” (CAT) software application. This archive also contains the plots of all curves in Portable Network Graphics (PNG) format. (ZIP) [file pone.0136131.s002.zip › data/097.png]

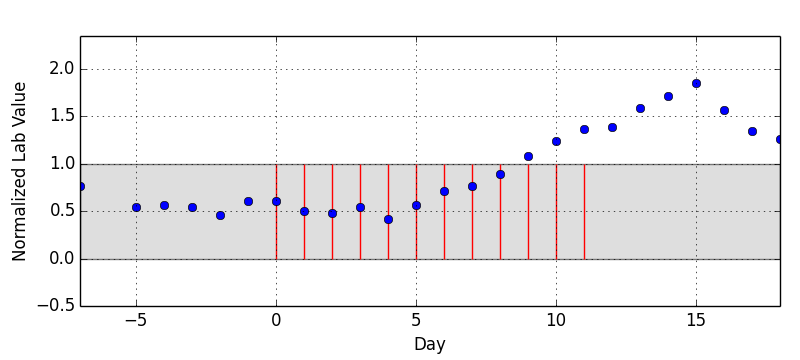

Supplement: S2 File — The “Curve Assessment Tool” (CAT) software application. This archive also contains the plots of all curves in Portable Network Graphics (PNG) format. (ZIP) [file pone.0136131.s002.zip › data/098.png]

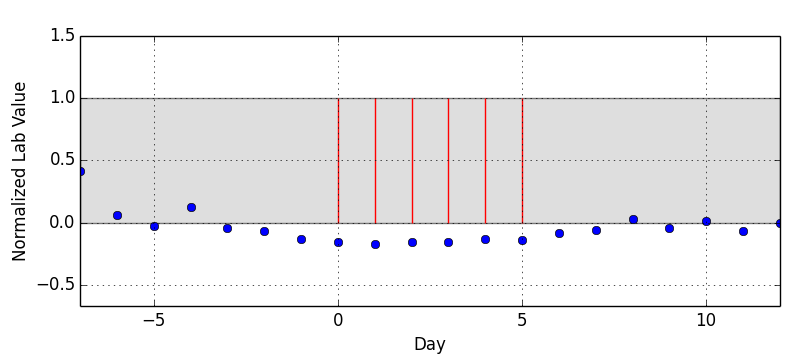

Supplement: S2 File — The “Curve Assessment Tool” (CAT) software application. This archive also contains the plots of all curves in Portable Network Graphics (PNG) format. (ZIP) [file pone.0136131.s002.zip › data/099.png]
